# Intermolecular packing effects on the two-photon absorption of the H_4_TCPE linker

**DOI:** 10.3389/fchem.2026.1805869

**Published:** 2026-05-21

**Authors:** Helmy Pacheco Hernandez, Mariana Kozlowska

**Affiliations:** Institute of Nanotechnology (INT) Karlsruhe Institute of Technology (KIT) Kaiserstraße 12, Karlsruhe, Germany

**Keywords:** metal-organic frameworks, nonlinear response, structure-property relationship, time-dependent density functional theory, two-photon absorption

## Abstract

Materials exhibiting efficient nonlinear optical properties, such as two-photon absorption (2PA), are of increasing interest for applications including biomedical imaging, optical data storage, and long-range communications. Metal-organic frameworks (MOFs) provide a versatile platform for enhancing such 2PA responses by allowing specific ordered arrangements of chromophores. In particular, organic molecules exhibiting aggregation-induced emission (AIE) have shown a significant increase in 2PA cross sections when incorporated into MOFs as linkers. This is possible not only because of the periodic ordered arrangement of photoactive molecules, but also due to subtle changes to the electronic properties of AIE-linkers and hindering their structural flexibility. The latter has been reported for tetrakis [4-(4-carboxyphenyl)phenyl]ethylene, known as H_4_TCPE, a linker used for MOFs with strong and tunable 2PA response. It is known that confinement effects in MOFs and the restriction of intramolecular motion of H_4_TCPE are key to its enhanced 2PA. However, the influence of specific intermolecular packing arrangements of H_4_TCPE on the 2PA response remains insufficiently understood. In this work, we study computationally the 2PA response of H_4_TCPE as a function of intra- and intermolecular packing. Using finite (non-periodic) monomeric and dimeric models, we first investigate how intramolecular flexibility affects the absorption spectra and 2PA cross sections and later analyze dimer configurations by varying the relative orientations to assess how intermolecular packing modulates the 2PA response. Finally, we compare the most favorable computed arrangements with experimentally reported H_4_TCPE-based MOF structures and propose optimal conformations for an efficient response.

## 1 Introduction

Nonlinear optical properties (NLO) describe the interactions between light and matter in which the material response depends nonlinearly on the intensity of the incident light (Boyd, 2020). Such phenomena typically occur under intense laser irradiation and enable a wide range of effects, including multiphoton absorption (MPA) (Weishäupl et al., 2022a), multiphoton-excited fluorescence (MPEF) (Huo et al., 2025), frequency upconversion processes such as second- and third-harmonic generation, among others (Zaręba et al., 2021).

MPA is a process in which two or more photons of the same or different wavelengths are absorbed to reach an excited state of a material, which may subsequently relax by emitting light (Benninger and Piston, 2013) or via nonradiative decay pathways (Shustova et al., 2012). This mechanism can access electronic transitions that are one-photon forbidden or weakly allowed, enabling access to high-energy excited states without high-energy photons (Medishetty et al., 2017a). Although multiphoton absorption can occur at any wavelength, it is most commonly realized using long-wavelength radiation to suppress linear absorption and enable the high peak intensities required for nonlinear excitation. Thus, it permits deeper light penetration inside the material, reduction of scattering effects, more precise control of excitation volume, as well as reduced out-of-focus photodamage (Benninger and Piston, 2013; Zaręba et al., 2021). As a result, materials with MPA functionality are attractive for applications in biomedical imaging (Benninger and Piston, 2013; Zipfel et al., 2003). Moreover, they can be used for 3D optical data storage, long-range telecommunications, and advanced anticounterfeit measures, among others (He et al., 2008; Pawlicki et al., 2009). In the 2PA process, a system makes a transition from its ground state to an excited state by the absorption of two photons. Because 2PA is a nonlinear optical process mediated by a virtual intermediate state, its probability scales quadratically with the light intensity and it can be used to populate states that are weak or inaccessible under one-photon excitation (Boyd, 2020). The absorption cross section σ_2_ and see Equations 1,2 describes the strength of this process and is usually measured in Göppert-Mayer units $\left(\text{GM}=10^{-50}{cm}^{4}\cdot s\cdot {molecule}^{-1}\cdot {photon}^{-1}\right).$ (Boyd, 2020).

In organic molecules, larger 2PA cross section values are generally associated with chromophores that combine extended π-conjugation length (Lee et al., 2005), and the presence of intramolecular charge transfer (Cesaretti et al., 2020; Medishetty et al., 2017b; Weishäupl et al., 2022a; Zhang et al., 2017). The extended π-electron delocalization permits electrons to move more easily over the molecule, enhancing the molecular polarizability, which reflects the capability of the electron cloud of a molecule to be distorted by an external electric field. It contributes to an increase in transition dipole moments between the electronic states, supporting strong nonlinear optical response (Medishetty et al., 2017b; Terenziani et al., 2008). Furthermore, the presence of intramolecular charge transfer (CT) character of the molecule increases the change in polarizability upon excitation and strengthens dipole coupling through low-lying charge-transfer intermediate states, additionally enhancing 2PA. Thus, many efficient 2PA chromophores are frequently built from aromatic or heteroaromatic cores connected by conjugated bridges, enabling π-electron delocalization and large transition dipole moments (Albota et al., 1998; He et al., 2008). Over the past decades, reported molecular 2PA cross sections for organic dyes have increased from roughly 1–10^2^ GM to values exceeding 10^3^ GM (He et al., 2008). The response of some chromophores may be additionally enhanced by their aggregation, permitting, for example, aggregation-induced emission. It is particularly applicable for structurally flexible chromophores characterized by nonradiative decays in solution: due to the restriction of their intramolecular motions upon aggregate formation (Haldar et al., 2020), efficient harvesting of 2PA-excited states (Mei et al., 2015) is possible. Moreover, aggregation may also enable extended conjugation between chromophores, increasing their polarizability and charge distribution between the molecules, thus, stronger dipole coupling and often intramolecular and intermolecular CT (Beerepoot et al., 2014; Valandro et al., 2020), enhancing 2PA. However, in some cases, due to the π- π stacking of chromophores in their aggregates, 2PA can also be quenched (Zeman et al., 2022), thus, ways to increase the concentration of 2PA active moieties embedded in tunable and controlled structures, hindering quenching events, are an actively developing field of research. It is known that the incorporation of 2PA chromophores as photoactive linkers into MOFs, can result in cross sections even of 10^7^ GM (Liu et al., 2022), as the framework immobilizes them in an ordered lattice, enabling high chromophore density and controlled alignment and spacing (Liu et al., 2022). This effect is particularly relevant for AIE-active linkers such as H_4_TCPE, where rigidification and ordered aggregation can suppress intramolecular vibrational and rotational motions, thereby reducing nonradiative decay and increasing the quantum yield (Hong et al., 2011; Shustova et al., 2012).

MOFs, often referred to as a subclass of coordination polymers, are porous crystalline materials that have been extensively investigated over the last decades (Yaghi et al., 1995) due to their versatility, programmability, and adaptability. They are characterized by a modular architecture comprising organic linkers and metal (or metal-oxo) nodes, which assemble into crystalline networks with diverse topologies and extend in one, two or three dimensions (Li et al., 2024). This modularity enables rational tuning of pore size and shape, surface area, and framework connectivity, allowing MOFs to be designed for specific target applications (Li et al., 2024; Pacheco Hernande et al., 2025), including 2PA (Deger et al., 2025). Several studies have incorporated 2PA-active chromophores, such as pyrene-based (H_4_TBAPy) (Deger et al., 2025) or carbazole-based (H_4_sbcd) (Deger et al., 2024; Weishäupl et al., 2022b), into MOFs to understand the structure-property relationships toward increasing of the 2PA response (Deger et al., 2025; Mayer et al., 2020; Medishetty et al., 2017a; Medishetty et al., 2017c). However, H_4_TCPE linker has been one of the most actively studied among others. For instance, Medishetty et al. (2017c) studied the incorporation of H_4_TCPE in In- and Zn-MOFs resulting in a 2PA action cross section ( $\left.\eta \sigma_{2}\right)$ of 3072 GM for In-MOF and 1053 GM for Zn-MOF. Later, Medishetty et al. (2017a) also investigated the same chromophore in a series of rigid Zr- and Hf-based MOFs with cubic or Kagome topology. They reported 2PA (ησ_2_) for six MOFs, with the parent H_4_TCPE chromophore showing ησ_2_ = 55 GM (per molecule), which increases to 3582 GM of the Zr/TFA/Kagome MOF upon linker incorporation into the framework. Furthermore, they investigated the chromophore’s flexibility and showed that by varying the intramolecular carbon-carbon distance (d_C-C_) between the H_4_TCPE arms, the 2PA response is maximized at a separation of about 16 Å. This was attributed to increased charge polarization of the linker, which also showed a maximum at 16 Å, and was quantified by the Hirshfeld-charge difference between the inner and outer phenyl rings. In the same line, Liu et al. (2022) synthesized five Zn-based MOFs employing the same H_4_TCPE linker, but the reported ησ_2_ varied substantially across the MOF series. In the isostructural pillared-layer MOFs, where different pillar linkers regulated the position and separation between the H_4_TCPE layer inkers, ησ_2_ reached ∼0.3–307 GM. In contrast, MOF with the most densely packed framework composed only of Zn(NO_3_)_2_ and H_4_TCPE, exhibited the highest ησ_2_ of 1.2 × 10^6^–7.4 × 10^7^ GM. Interestingly, Medishetty et al. (2017a) confirmed ησ_2_ of a structurally similar MOF to be 4300 GM. The observations described demonstrate a decisive role of molecular packing in enhancing the 2PA response. He et al. (2025) studied six Zr-based MOFs incorporating H_4_TCPE and reported a cross section (σ_2_) of 8801 GM (importantly, ησ_2_ ≠ σ_2_. The ησ_2_ is the product of quantum yield and the absolute σ_2_. Consequently, 2PA results are not directly comparable). They showed that the MOF response is strongly framework-dependent. Moreover, post-synthetic installation of secondary ligands changes connectivity and rigidity modulating packing density and linker conformation, while thermal activation further contracts the framework which all together impacts the 2PA response. Despite these advances, it remains unclear how aggregation in MOFs and specific intermolecular configurations modulate the 2PA, and which packing motifs enable the highest responses. Trial-and-error synthesis is cumbersome and time-consuming.

In this work, we use *in silico* screening combining density functional theory (DFT) and time-dependent DFT calculations to map how the 2PA response of H_4_TCPE varies with intra- and intermolecular geometry. We provide a systematic analysis of subtle electronic changes in diverse aggregation patterns of H_4​_TCPE, modulating the 2PA response, and explain observations that cannot be accounted for using macroscopic treatments of MOF materials. This allows us to move beyond the general observation that closer packing enhances the 2PA response and to identify which specific geometrical changes are favorable, as well as why they have this effect. To the best of our knowledge, a systematic study of how the relative packing arrangements of the H_4​_TCPE linker influence the 2PA response of its aggregates has not yet been carried out in a controlled manner. Thus, the present work not only rationalizes previous experimental observations but also provides an in-depth explanation of the phenomena. First, we assess how intramolecular flexibility (monomer arm conformations) affects the absorption spectra and the 2PA cross-sections ( $\left.\sigma_{2}\right)$ . Next, we evaluate dimer models by sampling packing arrangements (e.g., stacking distance and relative orientation) to quantify how these parameters regulate the 2PA response. Finally, we compare the most favorable motifs with experimentally reported H_4_TCPE-based structures and propose design opportunities that could be explored to enhance the 2PA response.

## 2 Methods and computational setup

### 2.1 Starting structures

**FIGURE 1:**
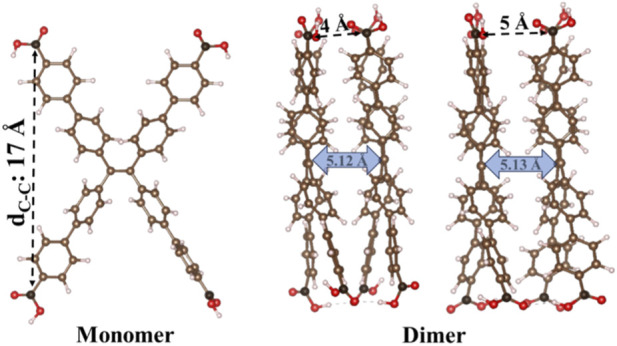
Schematic representation of the monomeric and dimeric H_4_TCPE models. In the monomer, the carbon atoms in black were fixed during geometry optimization to preserve specific intramolecular distances (d_C-C_), which were scanned from 13 to 19 Å. For the dimeric models, selected carbon atoms (in black) were fixed to preserve initial intermolecular stacking distances of 4 and 5 Å. After geometry optimization, the converged stacking distances resulted in intermolecular separation of 5.12 and 5.13 Å as indicated with blue arrows.

We started performing distance screening of the linker arms to generate initial structures of H_4_TCPE monomers by varying the distance d_C-C_ (see Figure 1) from 13 to 19 Å. The structures were further geometry optimized using the density functional theory approach as described in Section 2.2. For that, the ending carbon atoms were kept fixed while allowing the optimization of all remaining atomic positions. This screening permitted us to identify the most relevant structures that were used later for building the dimeric models. Dimers were built using d_C-C_ = 16–18 Å, which provides a more realistic representation since most of the experimentally reported d_C-C_ distances for this linker within MOF structures lay within this range of distances.

**FIGURE 2:**
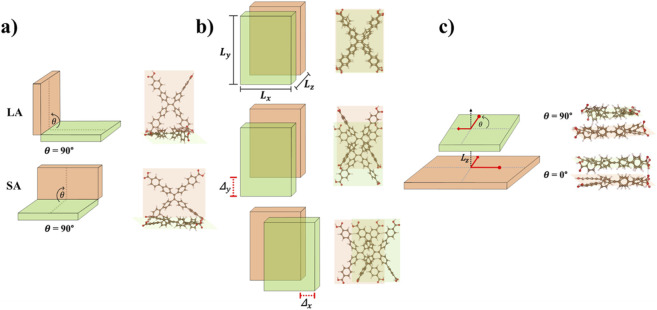
Schematic representation of the H_4_TCPE sampled dimeric models. **(a)** Book-like opening along the longer (LA) and shorter (SA) axes, showing the variation of the opening angle θ. **(b)** Stacking-plane displacement along the *x* and *y* directions. **(c)** Parallel-stacked dimers subjected to in-plane rotational scans, with the rotation angle θ.

Using the optimized monomer geometries as starting points, we generated dimer structures from monomers with d_C-C_ in the 16–18 Å range (see Figure 2) by systematically sampling key intermolecular degrees of freedom.

1. Book-opening angle: Dimer geometries were generated by first locating them on a 4 Å separation distance (see Figure 1) and then varying the opening angle between the two planes (see Figure 2a). Two possibilities were considered: an angle between the longer axis (LA) of the molecules, as well as the angle between the shorter axis of the molecule (SA). The book-opening angle was changed from 0° (parallel stacking at 4 Å) to 180° in increments of 45° (see Figure 2a).
2. Stacking plane displacement: Two initial intermolecular distances were considered (4 and 5 Å, as marked in Figure 1), represented by L_z_ in Figures 2b,c. For each separation, lateral displacements were applied by shifting one monomer relative to the other along the x (left-right) direction, $\Delta_{x}=\pm 5,\pm 10\;Å$ , and along the y (up-down) direction, $\Delta_{y}=\pm 5,\pm 10\;Å$ .
3. Plane rotation angle:As in the previous case, the two monomers were positioned at initial separations of L_z_ = 4 or 5 Å. One monomer was rotated relative to the other from 0° to 90° in 30° increments.

In all cases, only the terminal carbon atoms were fixed (marked in black in Figure 1) and all other atomic positions were fully relaxed.

### 2.2 Ground state calculations

Considering the π-conjugated nature of the tetraphenylethylene-type linker and the expected charge-transfer contributions to its electronic structure, all gas-phase geometry optimizations were performed through DFT using the long-range corrected hybrid CAM-B3LYP (Yanai et al., 2004) functional with def2-SVP (split-valence polarized) basis set (Weigend and Ahlrichs, 2005) and D3 dispersion correction with Becke-Johnson damping (D3BJ) (Grimme et al., 2011) in Gaussian16 (Frisch et al., 2016) (RevC.01). The energy convergence threshold for the geometry optimization of monomers and generated dimers was set to 1 × 10^−8^ Hartree. A custom Python script was used to systematically impose arm-distance constraints and generate the initial dimer arrangements. Vibrational analysis was performed to confirm that optimized structures correspond to true minima.

### 2.3 Excited stated calculations

One-photon absorption (1PA) spectra were computed using TD-DFT in Gaussian16 with the same computational setup as mentioned above. Ten singlet vertical excitations were considered. The 2PA cross-sections were calculated using Turbomole (V7.7) (Balasubramani et al., 2020) with CAM-B3LYP/def2-SVP/D3(BJ) (Grimme et al., 2011; Weigend and Ahlrichs, 2005; Yanai et al., 2004) using identical structures as previously discussed. Benchmark studies have shown that CAM-B3LYP is among the best performing range-separated functionals for 2PA studies (Chołuj et al., 2022). Although absolute 2PA strengths are found to be 1.5 to 3 times smaller than coupled-cluster (Beerepoot et al., 2015), CAM-B3LYP provides a suitable compromise between computational cost and reliable qualitative trend analysis, especially in large systems as the ones studied here. The resolution of the identity (RI) approximation was used for all calculations in Turbomole. Ten singlet-excitations were considered for the 2PA calculation using the quadratic response theory (Parker et al., 2018). The single point calculation and energy convergence threshold was set to 1 × 10^−7^ Hartree. For further analysis of the electronic properties of the studied systems, the electron density difference (EDD) was analyzed together with the set of frontier and transition orbitals. The generated isosurfaces were visualized with Visual Molecular Dynamics (VMD) applying 0.02 a.u. isovalue for individual orbitals and 0.0005 a.u. for EDDs. The excitonic coupling for the lowest vertical excitation in Δy = 0 systems was evaluated using the excitation energy transfer (EET) formalism based on TD-DFT calculations, as implemented in Gaussian16.

### 2.4 Two-photon cross-section calculation

The 2PA cross-sections were obtained using the quadratic-response theory as implemented in Turbomole (Parker et al., 2018). Rotationally averaged transition strengths were evaluated for the parallel linearly polarized light. The base cross section ( $\sigma_{0}$ ) was obtained according to Equation 1:

$$\sigma_{0}=\frac{4\pi^{3}\alpha a_{0}^{5}}{c}\left(\omega_{1}\cdot \;\omega_{2}\cdot S\right),$$ (1)

where α is the fine-structure constant, $a_{0}$ the Bohr radius, c the speed of light, ​ $\omega_{1}\;and\;\omega_{2}$ are the photon frequencies, while S is the transition strength (in atomic units). Complete cross section was obtained by multiplying the base cross section $\sigma_{0}$ by the line-shape, according to Equation 2:

$$\sigma \left(\omega \right)=\sigma_{0}g\left(2\omega \right),$$ (2)

where $g\left(2\omega \right)$ is a normalized line-shape function used to broaden the discrete transitions into a continuous spectrum. The 2PA spectra (as well as 1PA) were generated by applying Lorentzian broadening with a full width at half maximum (FWHM) of 0.1 eV. Further details are provided in Section 1 of the SI.

## 3 Results

### 3.1 Monomer calculations

**FIGURE 3:**
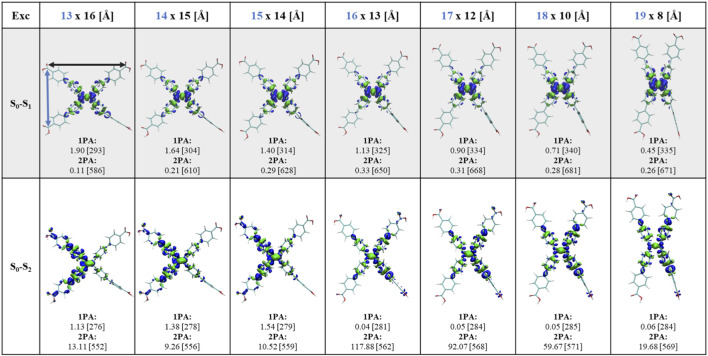
Visualization of electron density difference (EDD) in the two lowest singlet states in comparison to the ground state. 1PA and 2PA results of the excitations across the sampled distances d_C-C_ shown on the top in blue. Each monomer reports the 1PA oscillator strength in a.u. And the excitation wavelength (nm) in brackets. 2PA cross sections are reported in GM and the corresponding excitation wavelength (nm) is given in brackets. Electron density in blue denotes electron-deficient (donating) regions after excitation, meaning places from where electron density moved out, while electron density in green indicates electron-rich (accepting) regions after excitation, i.e., where it got accumulated.

After optimizing the monomer geometries across the sampled intramolecular d_C-C_, and the calculation of the relative energies between the optimized structures, we found an energy minimum at 17 Å (see Supplementary Table S1), consistent with previously reported studies (Medishetty et al., 2017a; Liu et al., 2022; Mayer et al., 2020; He et al., 2025; Gua et al., 2022; Yuan et al., 2024). 1PA and 2PA spectra were further calculated for all structures considered. Experimentally, the solid-state H_4_TCPE chromophore exhibits an UV-vis absorption maximum around 390–400 nm (Yuan et al., 2024; Mao et al., 2022) at d_C-C_ = ∼16.5 Å (Mao et al., 2022) and 18 Å (Yuan et al., 2024). In the same range (d_C-C_ = 16–18 Å), our TD-DFT calculations, applying CAM-B3LYP functional, result in a more blue-shifted first intense absorption band at 325–340 nm (see 1PA in Figure 3). Such an offset is consistent with the known tendency of CAM-B3LYP to overestimate vertical excitation energies on the order of tens of nanometers (e.g., ∼50 nm) relative to coupled-cluster CC2 benchmarks, which typically show improved agreement with experiment (Beerepoot et al., 2014; List et al., 2012). This also occurs for 2PA (Nayyar et al., 2013; Salem and Brown, 2014). Considering the approximately 60 nm blue-shift of the 1PA band, a two-fold increase in the 2PA wavelengths is expected. Experimentally, reported solid-state 2PA absorption maxima of H_4_TCPE span ∼670 nm (Mayer et al., 2020) to ∼780 nm (Chen et al., 2019). In the present study, we focus on the lowest energy regions of the spectra (i.e., first and second excitations) due to the higher relevance in experiments.

1PA and 2PA spectra calculations of the monomers reveal a progressive red-shift as d_C-C_ increases. For the first excitation, the 1PA band red-shifts by ∼10 nm, while the corresponding 2PA red-shifts by ∼20 nm (see 1PA and 2PA data in Figure 3 and spectra in Supplementary Figures S9, S10). To rationalize this trend, we examined the core phenyl torsions considering the C=C bond as depicted in Supplementary Figures S1. At d_C-C_ = 17 Å, the phenyl rings present the least twisted (i.e., the most planar) conformation among the sampled monomers. Increased planarity is generally associated with enhanced π-orbital overlap in conjugated systems with limited charge separation, which stabilizes the frontier orbitals and narrows the gap between the highest-occupied molecular orbital (HOMO) and the lowest-unoccupied molecular orbital (LUMO) gap, thereby reducing the vertical excitation energy (Phenomena, 2014; Salem et al., 2016; Juma Al-Busaidi et al., 2021). While across all the studied conformations (d_C-C_ = 13–19 Å) the first excitation (S_0_→S_1_) is predominantly occurring between HOMO and LUMO (see Supplementary Tables S1-S8), less planar geometries show a mixture of additional orbital transitions reflected in a reduced HOMO→LUMO contribution (e.g., 75.3% at 13 Å), whereas more planar conformations are more clearly described as a frontier-orbital transition (e.g., 85.8% at 17 Å). Thus, planarity modulates the spectral red-shift mostly *via* the change in transition orbitals and their contributions.

The EDD analysis shown in Figure 3 demonstrates additionally that the change in the intramolecular geometry and the compaction of the molecule modulate the excited-state electron-density redistribution and, consequently, affect the 1PA and 2PA responses in different ways. For S_0_→S_1_, increasing d_C-C_ leads to a gradual localization of the EDD on the inner phenyl rings, indicating a more core-centered excitation at larger separations. Consistently, the transition remains 1PA-bright, although the oscillator strength slightly decreases at larger d_C-C_, going from 1.90 (13 Å) to 0.45 at (19 Å). In contrast, the corresponding 2PA response is negligible, remaining nearly forbidden across the scanned conformations with only a small maximum of σ_2_ = 0.33 GM at d_C-C_ = 16 Å. For S_0_→S_2_, the changes in the EDD are more pronounced, the excitation evolves from one side of the molecule at 13 Å toward a more core-localized density distribution at larger d_C-C_. This evolution is accompanied by a strong suppression of 1PA especially for d_C-C_ = 16–19 Å, while 2PA is strongly activated, reaching its maximum value of σ_2_ = 117.88 GM at 16 Å. In this way, we observed that in the H_4_TCPE monomer 2PA predominantly populates the S_2_ state. This behavior is consistent with the selection rules discussed by Amar et al. (2021) for quadrupolar chromophores, where 2PA predominantly accesses the S_2_ state, which is 1PA forbidden or only weakly allowed under slight symmetry distortions.

To understand the change in the electronic properties of H_4_TCPE as a function of systematic compression of its arms in more detail and characterize the nature of the S_0_→S_1_ and S_0_→S_2_ excitations, we quantitatively analyzed the hole-electron distribution (Liu et al., 2020) using Multiwfn (Lu, 2024; Lu and Chen, 2012). For that, the Le Bahers et al. (2011) parameters for analysis of electron excitations based on EDD have been considered. The *Sr*, *D*, *H*_*CT*_, *H* and *t* indices are listed in Table 1. The *Sr* index indicates the degree of overlap of the hole and the electron (respectively, blue and green regions depicted in Figure 3), therefore values close to one indicate high overlap, thus a locally excited (LE) character of such a transition. Oppositely, values closer to 0 indicate high separation between hole and electron, thus a higher contribution of the charge transfer (CT). Going in line with *Sr*, the *D* index is the distance between the calculated centers of hole and electron, thus larger distances (e.g., >2 Å) indicate a more pronounced CT character. *H* index is the average degree of spatial extension of the hole and electron in the x/y/z and *H*_*CT*_ refers to the charge transfer direction. Finally, *t* index measures the separation degree of hole-electron in the CT direction, so if *t* index is < 0, this indicates LE, while positive values indicate CT. For the S_0_→S_1_ excitation, the hole-electron overlap remains moderately high across the scanned geometries (*Sr* = ∼0.75–0.79), indicating a predominantly LE character. The CT contribution is weak, as reflected by the small distance between the hole and electron centroids (*D* = 0.50–0.67 Å) and the consistently negative *t* index. Consistently, the dipole-moment change upon excitation is modest (*Δμ* = 0.89–1.17 a.u.; 2.27–2.98 D) and the spatial extension (*H*) decreases with increasing d_C-C_, consistent with the more core-localized redistribution observed in the EDD plots (see upper panel in Figure 3). Overall, S_1_ presents almost negligible changes in the CT parameters when the d_C-C_ increases, remaining mostly LE. The S_0_→S_2_ case reveals a more geometry-dependent and partially mixed character. At shorter d_C-C_ = 13–15 Å, the orbital overlap (*Sr* = 0.64–0.67) is lower than S_1_ case. This is accompanied by a stronger *Δμ* (peaks at 2.20 a. u [5.59 D] at 15 Å) and a larger spatial extension (*H* = 5.81–5.95 Å) than in S_1_ as visible in the EDD in bottom panel of Figure 3. At larger separations (d_C-C_ = 16–19 Å) the behavior changes. The excitations become more LE-like, the orbital overlap increases *Sr* = 0.72–0.74, *D* = 0.50–0.67 Å, with a less strong change in *Δμ*. The spatial extension is slightly reduced but still larger than for S_1_ (the largest *H* of 5.71 Å at 16 Å). These observations indicate different character of electronic transitions with a stronger influence of structural changes reflected in a S_0_→S_2_ transition. Partially CT-mixed states occur at shorter d_C-C_ with a shift to predominantly LE-dominated excitations at larger distances, however, in both cases the electron-hole pair remains delocalized over several conjugated segments, preserving a moderate exciton size despite the increased locally excited character.

**TABLE 1:** CT parameters for the S_0_→S_1_ and S_0_→S_2_ excitations of H_4_TCPE at different d_C-C_ distances. The dihedral angle is reported in degrees (°), values closer to 180° indicate a more planar structure. *Sr* is dimensionless, *D*, *H*_*CT*_, *H*, and *t* are given in angstroms. The variation of dipole moment with respect to ground state (*Δμ*) is in atomic units (a.u.) and in Debye [D].

d_C–C_ (Å)	ω (°)	Exc	Sr	D (Å)	Δμ (a.u.) [D]	H_CT_ (Å)	H (Å)	t (Å)
13	125.86	S_0_→S_1_	0.77	0.59	1.06 [2.69]	3.18	5.06	−2.59
		S_0_→S_2_	0.67	1.03	1.71 [4.36]	3.42	5.81	−2.38
14	128.86	S_0_→S_1_	0.79	0.53	0.96 [2.45]	3.18	4.83	−2.65
		S_0_→S_2_	0.65	0.89	1.45 [3.69]	3.60	5.95	−2.71
15	130.56	S_0_→S_1_	0.75	0.54	0.95 [2.42]	3.14	4.56	−2.59
		S_0_→S_2_	0.64	1.35	2.20 [5.59]	3.33	5.82	−1.97
16	131.84	S_0_→S_1_	0.77	0.51	0.90 [2.30]	3.22	4.47	−2.71
		S_0_→S_2_	0.72	0.67	1.16 [2.94]	2.98	5.71	−2.30
17	132.37	S_0_→S_1_	0.77	0.50	0.89 [2.27]	3.30	4.38	−2.80
		S_0_→S_2_	0.74	0.60	1.04 [2.66]	2.57	5.56	−1.97
18	131.57	S_0_→S_1_	0.76	0.54	0.94 [2.39]	3.30	4.25	−2.76
		S_0_→S_2_	0.74	0.51	0.89 [2.28]	2.42	5.53	−1.91
19	125.83	S_0_→S_1_	0.75	0.67	1.17 [2.98]	3.44	4.20	−2.76
		S_0_→S_2_	0.72	0.50	0.84 [2.14]	2.13	5.60	−1.63

In this scan, we systematically varied d_C-C_ to understand how intramolecular arm distances tune the electronic properties of the chromophore. However, the optimized structures at the extremes of the scan are substantially higher in relative energy (see Supplementary Table S1), suggesting that such conformations are unlikely to be form under realistic conditions. Therefore, for constructing the intermolecular packing models, we focus on the energetically accessible range d_C-C_ = 16–18 Å.

### 3.2 Dimer calculations

**FIGURE 4:**
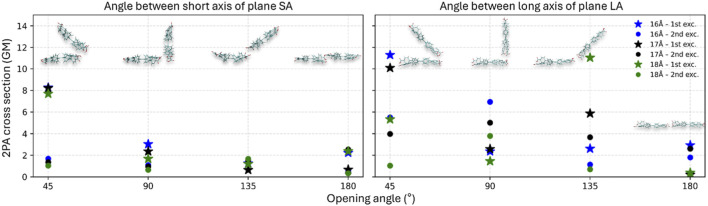
2PA cross sections in GM at different opening angle. The SA opening scan is shown in the left panel and the LA opening scan in the right panel. Results for the first excitation are marked with stars, and those for the second excitation with dots. Colors indicate the intramolecular d_C-C_ = 16 Å (blue), 17 Å (black), and 18 Å (green) of monomers in respective dimers. Representative geometries for selected configurations are shown as insets.

To understand the role of a dimer formation representing a fragment of molecular packing present in coordinated networks like MOFs, we constructed H_4_TCPE dimer models from the optimized monomers with d_C-C_ of 16–18 Å. Each monomer was duplicated, initially stacking and fixing the terminal carbon atoms, which preserves not only the intramolecular d_C-C_ (16–18 Å) but also defines the intermolecular separation. Due to the fact that intermolecular distance can strongly influence the electronic and optical properties of organic chromophores (Deger et al., 2025; Mostaghimi et al., 2023), two initial stacking distances were considered (4 and 5 Å). Variations in intermolecular separation were applied only for models ii) stacking plane displacement and iii) plane rotation, whereas for i) the book-opening model the initial stacking distance was fixed at 4 Å and only the opening angle was modified. All systems were further optimized as described in Section 2.2. The terminal carbon atoms remained fixed; however, all the other atoms were relaxed, and the optimized dimers converged to a final stacking distance of ∼5 Å, see blue arrow in Figure 1. The complete 1PA and 2PA results (10 excitations) for the three categories of dimeric systems are available in SI (see Supplementary Tables S1-S8; Supplementary Table S10-S21). In Figures 4, 6 we plotted and compared the 2PA cross sections of the first and second excitations, represented with a star and a dot, respectively.

#### 3.2.1 Book-opening angle

Since H_4_TCPE is anisotropic (Meng et al., 2023), short and long axis were identified (see Figure 2A). We investigated opening angles along both directions by varying the angle between the two molecular planes. SA is defined as the change in *θ* measured along the short axis of the planes, shown as insets on the left panel Figure 4. Analogously, LA refers to *θ* measured along the long axis (see right panel Figure 4).

**FIGURE 5:**
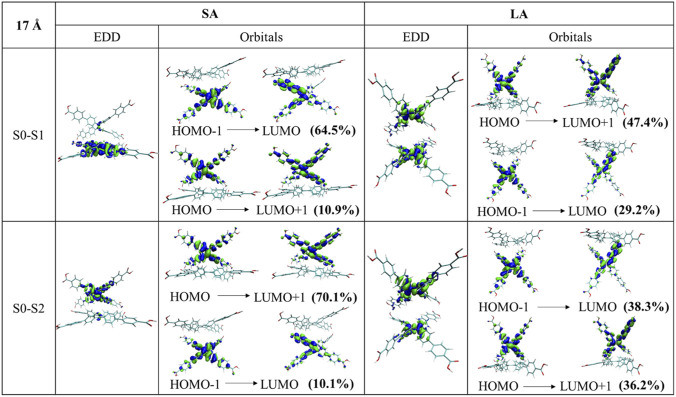
Book-opening angle: EDD and pair of the main orbital transitions for first and second excitation. Comparison of results for short (left) and long (right) axes angle opening for d_C-C_ = 17 Å, θ = 45°. d_C-C_ = 16 and 18 Å conformations are available in Supplementary Figure S13. Electron density in blue denotes electron-deficient (donating), while electron density in green indicates electron-rich (accepting) regions after excitation.

The EDD plots in Figure 5, show the hole (blue) and electron (green) distribution upon the two first excitations which provides direct information of the excitation character. For both SA and LA, the EDD and dominant orbital contributions vary only minimally across the d_C-C_ = 16–18 Å, consistent with these conformers lying near the energy minimum (after geometry optimization calculations) shown in Supplementary Table S1, therefore exhibiting only small changes. Accordingly, we show the d_C-C_ = 17 Å as a representative case, while the complete set of EDD plots is provided in Supplementary Figure S13.

2PA σ_2_ were plotted for all the sampled opening angles in the SA and LA direction as shown in Figure 4. Since d_C-C_ is defined along the long molecular axis, the LA scan intrinsically modulates the geometry along the same direction that governs arm separation. Accordingly, the LA series displays a stronger dependence of σ_2_ as a function of d_C-C_ and θ compared with the SA series. In subsections below, we discuss representative cases of SA and LA dimers, explaining the reason for differences in their 2PA response. We have to note that the aggregate formation significantly changes electronic transitions, showing a split of the lowest transition and several orders of magnitude increase of 2PA cross sections of the monomeric S_0_→S_1_ transition (see Supplementary Tables S2-S9). Moreover, both lowest transitions in dimer models occur at approximately 650–700 nm (depending on a dimer type) and are 100 nm distant from higher excitations, which appear to be, generally, less impacted.

##### 3.2.1.1 SA geometry

At θ = 45°, the S_0_→S_1_ yields consistently large σ_2_ values across d_C-C_ = 16–18 Å (see left panel Figure 4): 8.29 GM at 653 nm (16 Å), 8.25 GM at 664 nm (17 Å), and 7.72 GM at 671 nm (18 Å). These values vary only minimally between 16 and 17 Å and decrease more at 18 Å. In contrast, the second excitation is ∼6 times smaller in comparison with S_1_ and remains lower for all three conformations: 1.70 GM at 640 nm (16 Å), 1.36 GM at 651 nm (17 Å), and 1.04 GM at 658 nm (18 Å). This marked separation between S_0_→S_1_ and S_0_→S_2_ indicates that, in the θ = 45° conformation, the lowest excited state is more 2PA allowed. Comparing these values to the corresponding monomers, the S_0_→S_1_ σ_2_ values are ∼26 times higher, consistent with a cooperative enhancement in the dimer (Collini, 2012). Therefore, we further analyze the electronic characteristics of this enhancement *via* EDD and frontier-orbital contributions (Figure 5 for 17 Å; Supplementary Figure S13 for 16 and 18 Å). As depicted in Figure 4, increasing the opening angle reduces the response. At θ = 90°, σ_2_ for the S_1_ drops to ∼1.7–3.0 GM and the S_2_ remains small (∼0.6–1.0 GM). This decrease is consistent with reduced effective π-π interaction and a less favorable alignment, which weakens constructive contributions between the chromophores. At θ = 135°, both excitations remain small, with S_1_ ∼0.6–1.6 GM. At θ = 180°, the σ_2_ values recover modestly, showing ∼0.6–2.4 GM for the S_1_ and ∼0.3–2.5 GM for the S_2_. Overall, the SA scan suggests strong coupling at θ = 45° when the chromophores are in the closest proximity, followed by a progressive loss of coupling as the dimer opens where at large opening angles, the dimers start to behave more like a monomer and less like a dimer, resulting in low *σ*_2_.

A more in detail study of the electronic characterization was made specially for θ = 45° (see SA in Figure 5). We observed that for S_0_→S_1_, the EDD is largely localized in one of the monomers. However, when looking closer in the dominant orbital transition for S_0_→S_1_ (HOMO-1→LUMO: 64.5%), it presents a charge redistribution of density within that monomer, i.e., intramolecular charge-transfer (IntraCT). In contrast, such IntraCT is much less pronounced for S_0_→S_2_, where the dominant HOMO→LUMO+1: 70.1% transition, shows that density is more delocalized across one monomer, with only minor charge redistribution. In line with this, the Multiwfn analysis (see Supplementary Table S10) across the d_C-C_ = 16–18 Å shows a systematically larger hole-electron separation for S_0_→S_1_ (*D* = ∼1.36–1.50 Å), supporting a stronger IntraCT than for S_0_→S_2_ (*D* = 0.2–0.73 Å).

##### 3.2.1.2 LA geometry

In the LA series, σ_2_ values are overall higher than in the SA-based structures (see right panel in Figure 4), reflecting that the opening is performed along the same long-axis direction that defines d_C-C_ and therefore more strongly modulates the intermolecular arrangement. In this case, the highest response is again observed at θ = 45°. For S_0_→S_1,_ the *σ*_2_ across the three scanned distances are: 11.30 GM at 654 nm (16 Å), 10.11 GM at 677 nm (17 Å), and 5.34 GM at 683 nm (18 Å). For S_0_→S_2_, the *σ*_2_ decreases to 5.52 GM at 644 nm (16 Å), 3.98 GM at 667 nm (17 Å), and 1.04 GM at 675 nm (18 Å) (complete results for the first ten excitations are provided in Supplementary Table S6). Here, as observed in the SA scan, *σ*_2_ decreases at larger opening angles as the intermolecular coupling is reduced (Zeman et al., 2022). At θ = 90°, the S_1_ is strongly suppressed (approximately a 4.5-fold decrease relative to θ = 45°), whereas the S_2_ decreases more moderately (∼1.5-fold), indicating that at θ = 90° the orientation strongly diminishes the 2PA strength of S_0_→S_1_, while S_0_→S_2_ keeps comparatively stronger 2PA character. At θ = 180°, both excitations remain weak (∼0.2–2.9 GM for S_0_→S_1_ and ∼0.1–2.0 GM for S_0_→S_2_), consistent with the weakest coupling in this more open geometry. The θ = 135° case is notable. Unlike the other angles, where *σ*_2_ values among the three d_C-C_ conformations typically differ by only ∼1–2 GM, the LA (135°) structures show a marked difference in the 2PA response depending on the d_C-C_ conformation. For example, while the S_0_→S_2_ remains comparatively low (∼0.7–3.6 GM), the S_0_→S_1_ increases markedly: ∼2.6 GM (16 Å), ∼5.8 GM (17 Å), and ∼11 GM (18 Å), suggesting that at θ = 135° in the LA scan, the 18 Å conformation can be favorable for enhancing *σ*_2_.

A study of the electronic character was carried out for the most strongly coupled conformation (θ = 45°) to compare the density redistribution with the SA case (see Figure 5). For the S_0_→S_1_ excitation, the main pair of orbital transitions suggest important contributions from different orbitals, for instance, HOMO to LUMO+1 (47.4%) and HOMO-1 to LUMO (29.2%), where the orbitals are mostly localized on different monomers. Thus, the EDD for S_0_→S_1_ excitation appear to look completely different than in the SA case: it shows a coherently delocalized excitation on the two monomers instead of dominant single molecule electron density changes. We therefore conclude that this excitation is a Frenkel-type exciton, which is locally excited on each monomer but coherently coupled in the dimer (Schröter et al., 2015), allowing the 2PA response to increase in this dimer by 10% in comparison to the SA-based structures. The changes described appear to be of a similar nature also for the S_0_→S_2_ excitation. The analysis of CT parameters of the lowest excitation S_0_→S_1_ for θ = 45° SA and LA conformations (see Supplementary Table S10) confirms the visual inspection (Figure 5), showing a distance between the hole-electron for LA (45°) of *D* = 0.50 Å (d_C-C_ = 17 Å) instead of *D* = 1.40 Å for SA (45°) and slightly higher spatial extension of the charge over the system for LA (45°): *H*_*CT*_ = 4 Å, instead of *H*_*CT*_ = 3.35 Å for SA. Furthermore, trends of the 2PA σ_2_ correlate with the changes in the isotropic polarizability as depicted in Supplementary Figure S14.

#### 3.2.2 Stacking plane displacement

**FIGURE 6:**
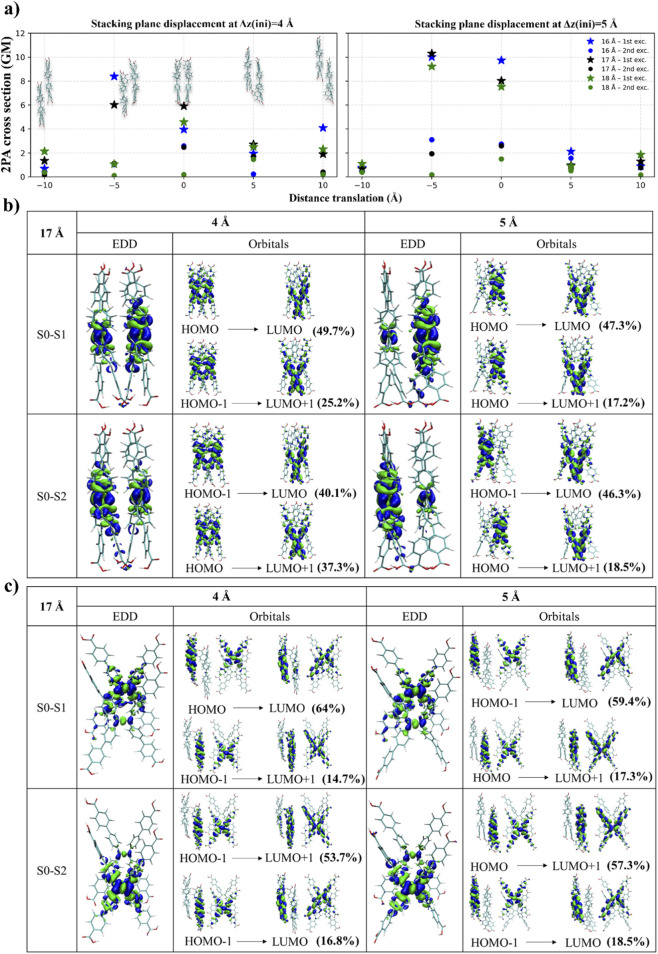
**(a)** Effect of stacking-plane displacement on the 2PA of H_4_TCPE dimers. The σ_2_ as a function of in-plane displacement distance (±5 and 10 Å) at initial stacking distances Δz (ini) = 4 Å (left) and 5 Å (right). Stars and circles denote the first and second excited-state transitions, respectively. Colors indicate the intramolecular d_C-C_: 16 Å (blue), 17 Å (black), and 18 Å (green). Representative geometries for selected configurations are shown as insets. EDD and pair of the main orbital transitions for first and second excitation. Comparison of results for **(b)** Δy = 0 Å and **(c)** Δy = −5 Å for d_C-C_ = 17 Å, Δz (ini) = 4 Å and 5 Å d_C-C_ = 16 and 18 Å conformations are available in Supplementary Figure S16-S19. Electron density in blue denotes electron-deficient (donating), while electron density in green indicates electron-rich (accepting) regions after excitation.

Since the formation of J- or H-aggregates impacts the 2PA σ_2_ (Zeman et al., 2022), here we explored somewhat similar linker orientation by modeling different displacements between the monomers in a dimer, as well as the displacement direction starting from a fully parallel stacked reference geometry (see Figure 2b), and applied lateral displacements within the stacking plane. Left-right displacements were defined as Δx = ±5 and ±10 Å, considering negative values going on the left direction and positive indicating right direction. Up-down displacements are denoted as Δy = ±5 and ±10 Å (positive = up, negative = down). The σ_2_ remained consistently low (∼0.1–3 GM) for the Δx displacements (see Supplementary Tables S15-S18). This likely arises because Δx displacements occur along the short axis of the chromophore, which rapidly reduces the π-π overlap area compared with Δy translations, which shift the dimer along the long axis. The resulting reduced π-contact is expected to weaken intermolecular electronic coupling, and therefore leads to smaller σ_2_ values. For this reason, the discussion below focuses primarily on the Δy series (±5 and ±10 Å), shown in the upper panel of Figure 6. The full dataset is provided in the Supporting Information (Supplementary Tables S11-S18).

It should be noted that the H_4_TCPE linker is not a typical chromophore aligning to traditional definition of J- and H-aggregates, which characterize with well-defined transition dipole moments and non-random relative orientation of a quadrupolar scaffold (Hestand et al., 2018; Hestand and Spano, 2017; Hochstrasser and Kasha, 1964; Kasha, 1963; Kasha et al., 1965; Spano and Silva, 2014). Since its aromatic units are connected by rotatable bonds and only a partial π-delocalization is present (see, e.g., orbitals in Supplementary Figures S2-S8), dipolar orientation of the molecule should be locally defined. Upon a detailed inspection, we saw that the linker contains multiple aromatic units with different spatial orientations, and even if the same displacement is applied, aggregation patterns are very different and complex. Thus, the aggregate effect cannot be unambiguously resolved into a single J- or H-type motif and the overall 2PA response reflects a superposition of contributions from differently coupled subunits. For example, displacements of Δy = ±5 in the upper panel of Figure 6 look to be more H-like-aggregate for Δy = −5 Å, while more like J-like for Δy = +5 Å, but the change in 2PA cross-section does not follow typical trends (Zeman et al., 2022). Similar observation was reported by Sanyal et al. (Sanyal et al., 2016) who demonstrated in detail that for multibranched or quadrupolar chromophores different parts of the molecule contribute transition densities oriented along different axes and aggregation does not produce a single, well-defined J- or H-aggregation motif. Furthermore, for boron-difluoride-curcuminoids and squaraines, they observed 2PA response to be a superposition of multiple excitonic couplings. Therefore, in the following, we focus mostly on the electron density changes induced by a different displacement packing.

Among the sampled displacement distances, Δy = 0 and Δy = 5 Å yields the largest 2PA cross sections. In this region, dimers initialized with a larger interplanar separation (Δz = 5 Å) consistently give higher σ_2_ values than those started at Δz = 4 Å. Overall, this displacement shows lower dependence on the d_C-C_ distance in comparison to the book opening dimers, e.g., LA at 45° or 135° (see Figure 4). Since displacement of Δy = −5 Å indicates larger differences between Δz = 4 and 5 Å, we describe the systems in more detail below. Finally, it should be mentioned that dimers with the highest displacement of Δy = ±10 Å, shifting towards head-to-tail-like assemblies, results in a significant decrease in σ_2_ with values below 4 GM (or often below 2 GM).

##### 3.2.2.1 Displacement ▵y = 0 Å (or θ = 0°)

For the S_0_→S_1_ transition, σ_2_ values are around 3.95–5.90 GM when initial stacking distance is Δz = 4 Å, and increases to 7.53–9.73 GM at Δz = 5 Å, across the sampled distances. For S_0_→S_2_, σ_2_ remains smaller, ranging from 0.2–2.5 GM (Δz = 4 Å) and 1.5–2.7 GM (Δz = 5 Å). As previously described, after optimization, both sets converge to a very similar interplanar distance (∼5.1 Å). Therefore, the remaining structural differences are mainly localized at the linker termini, rather than in the stacking region (Figure 1). Consistent with these trends, the EDD plots and the dominant orbital contribution (see Figure 6) show that for the Δz = 4 Å, the S_0_→S_1_ excitation involves pronounced density redistribution within the dimer, going from the inner region of both monomers (see the HOMO orbital in Figure 6b) toward the extremities arm endings that occupy the LUMO orbital with a contribution of 49.7%. However, the LUMO orbital is not delocalized in the same manner as HOMO and a stronger localization in both monomers occurs. Even if this transition comprises other transition orbitals with much smaller contributions, for example, of 25.2% (HOMO-1→LUMO), the EDD shows a strong exciton delocalization between both monomers. Delocalized EDD is visible in the previously discussed LA-dimer case in Figure 5 (with θ = 45°). However, there the excitation occurred on each of the monomer separately, while here it is clearly delocalized within the dimer on the HOMO orbitals and more localized on the LUMO orbital. The excitonic coupling in Δy = 0 Å, Δz = 4 Å is +54 meV, while +26 meV for the LA (45°) case, additionally confirming stronger excitation delocalization as explained from the EDD. Furthermore, the resulting asymmetry in electron delocalization induces partial dipolar character: the oscillator strength for Δy = 0 Å, Δz = 4 Å, d_C-C_ = 17 Å is 0.102 (see Supplementary Table S19), while it is 0.034 for LA (45°), d_C-C_ = 17 Å as shown in Supplementary Table S6. Moreover, stronger CT is clear from the data listed in Table 2, where *Sr* for Δz = 4 Å depicted in Figure 6 (d_C-C_ = 17 Å and Δy = 0) is 0.61, showing lower hole-electron overlap, but it is 0.64 for the LA case (see Supplementary Table S10), and the hole-electron separation centroid is *D* = 1.55 Å instead of *D* = 1.40 Å. Such observation indicates a mixed Frenkel-CT exciton rather than a purely symmetric Frenkel exciton. Since excitonic coupling can redistribute 2PA strength among excitonic states, such that increased dipolar mixing reduces the σ_2_ of the lowest excitonic transition while enhancing higher-lying excitonic states, we believe that this is the reason why we see a slight decrease of the 2PA σ_2_ of Δz = 4 Å (d_C-C_ = 17 Å and Δy = 0). It is lower than in the LA (45°) dimer, i.e., it is around 3.95–5.90 GM (see Supplementary Table S19) instead of 5.33–11.29 GM for the LA (45°) dimer (see Supplementary Table S6). Furthermore, the effect may be partially related to decreased effective molecular size (LA has a different orientation) and decreased transition polarizability, which is 1,446.77 a.u. for LA (d_C-C_ = 17 Å, θ = 45°), see Supplementary Figure S14, while 1,387.27 a.u. for (d_C-C_ = 17 Å and θ = 0°), see Supplementary Figure S21.

**TABLE 2:** CT parameters for the S_0_→S_1_ excitations of H_4_TCPE dimer at d_C-C_ = 16–18 Å, with Δy = 0 and −5 Å. *Sr* is dimensionless, *D*, *H*_*CT*_, *H*, and *t* are given in Angstroms. The variation of dipole moment with respect to ground state (*Δμ*) in atomic units (a.u.) and in Debye [D].

Δz (Å)	d_C–C_ (Å)	Sr	D (Å)	Δμ (a.u.) [D]	H_CT_ (Å)	H (Å)	t (Å)
Δy = 0							
4	16	0.65	0.57	0.91 [2.32]	3.72	5.54	−3.15
	17	0.61	1.55	2.38 [6.05]	3.85	5.53	−2.29
	18	0.65	1.71	2.74 [6.96]	3.80	5.09	−2.09
5	16	0.60	1.43	2.17 [5.51]	3.50	5.40	−2.06
	17	0.62	1.13	1.74 [4.43]	3.58	5.31	−2.44
	18	0.65	0.58	0.92 [2.36]	3.59	5.58	−3.00
Δy = -5							
4	16	0.64	1.66	2.66 [6.76]	3.19	4.99	−1.53
	17	0.69	1.33	2.21 [5.62]	3.81	5.19	−2.47
	18	0.72	1.09	1.85 [4.70]	3.72	5.02	−2.63
5	16	0.60	1.64	2.53 [6.44]	3.41	5.51	−1.76
	17	0.61	1.51	2.32 [5.91]	3.71	5.35	−2.2
	18	0.65	1.57	2.50 [6.36]	3.28	5.09	−1.70

More relaxed positioning of arm endings in Δz = 5 Å system (see right panel in Figure 6a; note that stacking distance in both cases are similar, i.e. 5.12 and 5.13 Å, see Figure 1) results in significant change in charge delocalization. At Δz = 4 Å, the density is more evenly distributed over the dimer, whereas at Δz = 5 Å it becomes predominantly localized on a single monomer. The exciton coupling decreases to +47.5 meV at Δz = 5 Å, however, it mostly indicates that the pure Frenkel exciton contribution slightly decreases. For the S_0_→S_1_ transition, the excitation is mainly described by HOMO→LUMO (47.3%) and HOMO→LUMO+1 (17.2%) transitions where a pronounced charge redistribution is visible (Figure 6b). Here, the electron density shifts from largely delocalized in one of the monomers (HOMO) to more localized regions near the arm ends on both monomers (LUMO and LUMO+1). This permits the 2PA increase for S_0_→S_1_ from ∼3.95–5.90 GM to ∼7.53–9.73 GM (see Figure 6a). A similar redistribution of electron density is observed for S_0_→S_2_ (see Figure 6b). Since both excitations are close in energy (see Supplementary Table S19, the first excitation is at 706 and 694 nm for Δz = 4 and 5 Å systems, respectively, while their second excitation is at 681 and 670 nm), both excitations couple and contribute in the final 2PA response. These two cases demonstrate the impact of packing-induced asymmetry and changes in the coherence of multi-arm contributions on the cooperative enhancement of 2PA in H_4_TCPE. Molecular distortions arising from shorter intermolecular distances, maintained in our calculations by fixing the terminal C atoms to mimic denser packing, lead to a reduction in 2PA.

##### 3.2.2.2 Displacement ▵y = −5 Å

For S_0_→S_1_ at Δz = 4 Å, the main σ_2_ values are 8.40 GM at 671 nm (d_C-C_ = 16 Å), 6.03 GM at 692 nm (17 Å), and 1.09 GM at 725 nm (18 Å) as shown in Figure 6A. Increasing the initial separation to Δz = 5 Å changes σ_2_ to 10.02 GM at 668 nm (16 Å), 10.31 GM at 678 nm (17 Å), and 9.24 GM at 679 nm (18 Å). The qualitative EDD for Δz = 4 and 5 Å show only minor differences (see Δz = 4 vs. 5 Å in Figure 6C). For S_0_→S_1_, the main orbital contribution for Δz = 4 Å (64%) shows a small change from delocalization on the central part of one of the monomers (HOMO) to a more localized in the inferior part of the same monomer (LUMO). Although for Δz = 5 Å the main transition is described by HOMO-1→LUMO (59.4%), the electron density shows essentially the same pattern.

Due to the minimal changes in the qualitative analysis, we further analyze the excitation character with Multiwfn, summarized for the first excitation in Table 2. In the case of initial Δz = 4 Å, the sharp decrease in σ_2_ with increasing d_C-C_ correlates with a reduction between the hole and the electron distances. In this way, *D* decreases from 1.663 Å (16 Å) to 1.338 Å (17 Å) and 1.092 Å (18 Å) along with a decrease in the *Δμ*. Together, these trends indicate a progressively less CT-like behavior for S_0_→S_1_, thus lowering of 2PA response. At Δz = 5 Å, by contrast, *D* and *Δμ* remain comparatively stable with minimum difference of 0.07–0.14 Å across d_C-C_. This correlates with the comparatively stable σ_2_ results of 9.24–10.02 GM across the d_C-C_ systems.

**FIGURE 7:**
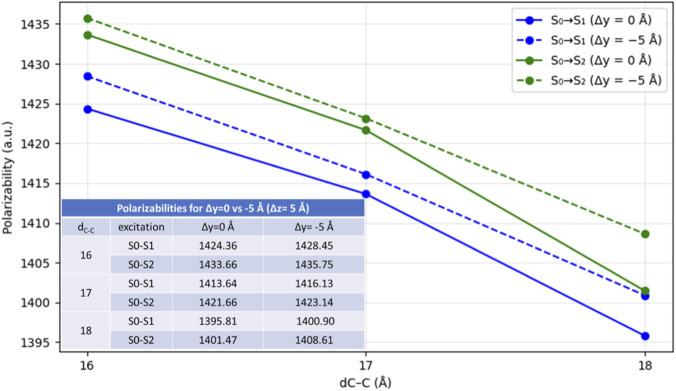
Comparison of the isotropic polarizability for Δy = 0 vs. −5 Å, at Δz (ini) = 5Å, across the sampled distances for the first (blue) and second (green) excitations. Solid line is representing 0 Å displacement (stacking) and dashed line represents −5 Å displacement.

Overall, the qualitative EDD and the orbital analysis does not provide an unambiguous explanation for the enhanced σ_2_ (Δy = −5 Å, Δz = 5 Å), motivating the additional Multiwfn analysis. Interestingly, when we compare Δy = 0 and Δy = −5 Å at Δz = 5 Å, results show that σ_2_ values do not increase with larger hole-electron separation (*D*) (i.e., higher CT-like behavior). In the Δy = −5 Å case, σ_2_ values are higher (9.24–10.31 GM) despite the smaller *D* than in Δy = 0 Å case, for which σ_2_ values are slightly lower (7.53–9.73 GM) even though *D* is larger. These results that might seem contradictory, indicating that the 2PA enhancement cannot be attributed in this packing primarily to increased CT-like character. Instead, for these conformations, the σ_2_ correlates more closely with the transition polarizability, where the Δy = −5 Å conformation is slightly better polarizable than Δy = 0 Å (see Figure 7). Displaced dimers (e.g., Δy = −5 Å) are generally more stable and, in the context of π-π stacking, typically exhibit stronger dispersion interactions than perfect parallel, or called eclipsed (Δy = 0 Å) dimers (Wheeler, 2025), implying a higher effective polarizability. The displacement reduces repulsion, changes how the chromophore overlaps and enhances polarization of electron density between the aromatic parts (Hunter and Sanders, 1990).

#### 3.2.3 Plane rotation angle

Finally, we investigated stacking plane rotations of θ = 0°, 30°, 60°, and 90° to assess their impact on the 2PA response (Supplementary Tables S19-S22; Supplementary Figure S22). Note that θ = 0° corresponds to the Δy = 0 Å case discussed in the previous section. Overall, the rotated geometries (30°, 60°, and 90°) show very weak 2PA, with only a modest recovery at θ = 90°. At θ = 30° (both Δz = 4 and 5 Å), the 2PA response is essentially quenched for all sampled d_C-C_ and for both excitations (σ_2_ < 0.5 GM). Interestingly, the S_0_→S_2_ state showed relatively large 1PA oscillator strengths (∼1.1–1.5) for d_C-C_ = 16–17 Å, whereas d_C-C_ = 18 Å presented reduced oscillator strengths of ∼0.6 (see Supplementary Table S20), indicating that these conformations are more 1PA active than 2PA in the excitation energies analyzed for all dimers. In the θ = 60°, only d_C-C_ = 17 Å, Δz = 5 Å presented a noticeable increase in σ_2_ (∼4.05 GM), but still low in comparison with previously analyzed systems (plane displacement and opening angle), while the d_C-C_ = 16 and 18 Å remain low (∼0.1–0.5 GM) and exhibit comparatively small oscillator strengths (Supplementary Table S21). Finally, 2PA at the θ = 90° case increases modestly than at 30° and 60°, with the most relevant 2PA σ_2_ values obtained for d_C-C_ = 16 Å (Δz = 4Å) of 1.7 and 5.5 GM for the S_0_→S_1_ and S_0_→S_2_ respectively, whereas d_C-C_ = 16 Å (Δz = 5Å) yields 1.9 GM (S_0_→S_1_) and 0.6 GM (S_0_→S_2_) (Supplementary Table S22). Because these rotated conformations generally exhibit low 2PA responses and are highly unlikely to be formed in MOFs with such packing conformations, we do not discuss their electronic structure in further detail.

## 4 Discussion

The aim of this study was to perform an *in silico* screening, combining density functional theory and time-dependent DFT, to systematically map how the two-photon absorption response of H_4_TCPE depends on different structural degrees of freedom. We first examined intramolecular flexibility by varying the d_C-C_ distance, in order to determine how this modulates the electronic structure, the nature of the low-lying excited states, and the resulting 2PA response. Within the monomeric series, we found that the linker exhibits a pronounced quadrupolar character, which leads to complementary one- and two-photon selection rules for the lowest singlet excitations: the S_0_→S_1_ transition is 1PA-allowed but 2PA-forbidden in d_C-C_ = 16–18 Å range, whereas the S_0_→S_2_ is 1PA-forbidden and 2PA-allowed. By EDD and main orbital contribution analysis, we observed that varying d_C-C_ primarily tunes the electron density of the lowest excited states in the H_4_TCPE monomer. In particular, the S_0_→S_1_ excitation remains largely locally excited across the scanned geometries, becoming progressively more core-centered as d_C-C_ increases. In contrast, the S_0_→S_2_ excitation is more sensitive to the intramolecular geometry changes. In this state, the EDD shows a more marked charge redistribution from one side of the monomer to the core rings in the experimentally relevant range (d_C-C_ ∼ 16–19 Å) consistent with the quantitative analysis performed in Multiwfn. Overall, these trends indicate that 2PA in the monomer predominantly populates the S_2_ state, consistent with the behavior expected for quadrupolar chromophores.

**FIGURE 8:**
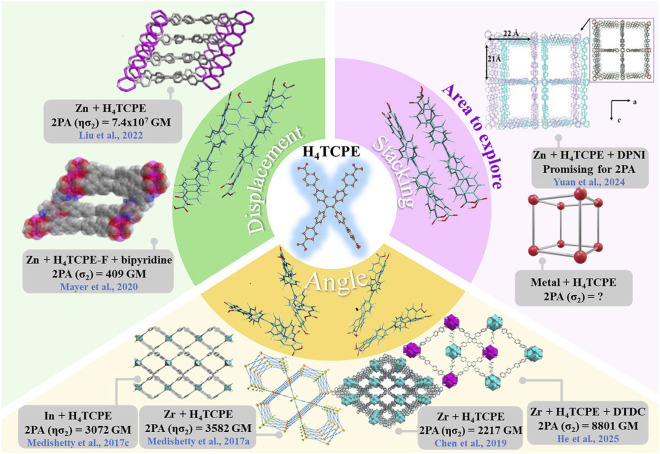
Overview of the reported 2PA performance in H_4_TCPE-based MOF and their associated topologies. Representative systems are divided into three different arrangements: i) displacement, ii) stacking, and iii) angle. Metal node and linkers are specified in each case. The reported 2PA values are shown in GM, including both σ_2_ and ησ_2_, as given in the original references. The pink section highlights promising but less explored design directions. Figure adapted from Ref (Chen et al., 2019; He et al., 2025; Yuan et al., 2024; Liu et al., 2022; Medishetty et al., 2017a; Medishetty et al., 2017c). with permissions from Jhon Wiley and Sons (© 2017, 2017, 2019, 2022, 2024, 2025), and Ref (Mayer et al., 2020). American Chemical Society, © 2020.

Secondly, monomers in the d_C-C_ = 16–18 Å window were used as building blocks to model different packing conformations. Three packing degrees of freedom were examined: i) book-like opening along the short and long molecular axes by varying the opening angle θ, ii) stacking-plane translations along Δx and Δy, and iii) in-plane rotations of parallel-stacked dimers by varying the rotation angle θ. In the book-opening scan, the SA (45°) dimers exhibit an enhanced 2PA response for S_0_→S_1_, consistent with a more pronounced intramolecular charge redistribution relative to S_0_→S_2_, as supported by the larger hole-electron separation (D = ∼1.36–1.50 Å for S_1_ vs. 0.2–0.73 Å for S_2_). In the LA (45°) case, the enhancement is instead associated with stronger intermolecular coupling and the formation of a coherently coupled Frenkel-type exciton delocalized over both monomers, giving ∼10% higher σ_2_ than SA. In the stacking-plane displacement scan, translations along Δx yield negligible σ_2_, indicating an unfavorable conformation for electronic coupling. In contrast, translations along Δy reveal high 2PA for both Δy = 0 and Δy = −5 Å. For Δy = 0, the EDD/orbital analysis indicates a mixed Frenkel/CT-like character that supports significant 2PA σ_2_. For Δy = −5 Å, σ_2_ becomes higher (particularly at Δz = 5 Å), and the trend correlates more closely with the polarizability than with charge-separation or CT-like behavior, suggesting that the displacement of the chromophores enhances the polarization response of the dimer in comparison with the parallel stacked conformation. Finally, in-plane rotational scans generally quenched 2PA at 30° and most 60° geometries, while 90° yields only a modest 2PA σ_2_. Regarding the interplanar separation Δz, applied only for cases ii) and iii), it impacted in different ways depending on the studied conformation. Although close chromophore proximity is often expected to strengthen coupling (Mostaghimi et al., 2023; Terenziani et al., 2008), our results indicate that enforcing an overly short initial separation (e.g., Δz = 4 Å) may impose geometric constraints, packing-induced asymmetry or generate less realistic arrangements, which can lead to a reduced 2PA response. Some of the local arrangements explored in this work are also found in experimentally reported H_4_TCPE-based MOFs and are summarized in Figure 8. However, a direct geometry-to-geometry comparison is not straightforward because complete structural information (e.g., accessible CIF files) is often unavailable, preventing extraction of the exact relative orientations and stacking modes between neighboring chromophores. Consequently, our comparison is mostly qualitative and focus more on the observed trends. For example, opening angle structures studied here resemble linker arrangements as reported in In-H_4_TCPE MOF (Medishetty et al., 2017c) and Zr-H_4_TCPE (Chen et al., 2019), which reported 2PA ησ_2_ of 3072 GM and 2217 GM, respectively.

Overall, our trends are consistent with prior observations, and in the cases where MOFs represent closer chromophore packing and, in particular, frameworks constructed exclusively from H_4_TCPE linkers, often display high 2PA responses. A notable example is the Zn-H_4_TCPE MOF, which is built only from H_4_TCPE linkers and where chromophores are slightly displaced but keeping a close proximity, and for which an exceptionally large 2PA ησ_2_ of ∼7.4 × 10^7^ GM (Liu et al., 2022) has been reported (see Figure 8). Importantly, a similar Zn-H_4_TCPE MOF with a closely packed linker arrangement was synthesized by Medishetty et al. (Medishetty et al., 2017a), but the reported ησ_2_ was 4300 GM. The linker stacking in those cases resembles the plane displacement systems presented in Section 3.2.2.2: close proximity between the linkers favors intermolecular coupling resulting in higher cross sections. By contrast, when packing changes to the opening-angle systems (see the angle-dependent MOFs in Figure 8), the interactions between neighboring molecules become less favorable, thus, reducing the 2PA response, similar as seen for the reported MOFs in Figure 8.

Finally, we did not identify any experimental MOF structures corresponding to a fully parallel stacking motif (Δy = 0 or θ = 0°) nor for plane rotation case (Section 3.2.3). Our screening suggests that near-parallel packing at moderately high chromophore density remains an attractive design opportunity. However, 2PA response could be less favorable than for the plane displacement case. In this arrangement, (Yuan et al., 2024) reported an interpenetrated pillared MOF (Zn-TCPE-DPNI) and proposed it as a promising platform for 2PA and even higher-order multiphoton processes (e.g., 3PA). However, the 2PA cross sections have not yet been reported.

In the end, we have to point out some limitations regarding our study. We emphasize that this work is a qualitative and comparative study aimed at identifying how relative arrangements of H_4_TCPE chromophores (packings that could occur within a MOF) are modulating the 2PA response. Our calculations rely on finite (non-periodic) models and therefore do not capture the long-range interactions that could be present in real periodic MOFs. For this reason, the results should not be interpreted as a direct comparison of absolute σ_2_ values with experiment, but rather as a qualitative comparison of how relative orientations influence the 2PA response. To the best of our knowledge, the calculation of MPA properties in periodic metal-organic systems with a large number of atoms per unit cell is currently computationally prohibitive. However, if the linker behaves nearly independently, their individual contributions to the 2PA response can be considered additive (Collini, 2012; Deger et al., 2025). Following this assumption, other references have estimated the 2PA response per monomer by calculating the 2PA tensor of H_4_TCPE, summing these tensors for all linkers in the unit cell, and applying orientational averaging to mimic randomly oriented MOF. Using this approach, effective 2PA cross sections per chromophore as high as 35 GM for cubic MOFs and 163 GM for Kagome MOFs have been reported (Medishetty et al., 2017a). In this way, while the relative trends associated with different molecular arrangements can be qualitatively compared, the absolute 2PA cross sections obtained in this work should be carefully compared.

Beyond the limitations of finite vs. periodic models, there are additional factors complicating a direct comparison to experiment. First, although experimental σ_2_ values are strongly influenced by the surrounding medium (e.g., solvent, crystal packing, interactions metal-ligand, or confinement within a MOF), our calculations were performed without explicit solvent and without considering the metal nodes. Metal nodes can modulate the 2PA response through polarization effects (Deger et al., 2025; Weishäupl et al., 2022b) and thereby alter the CT character of excitations. However, the metal node contribution depends on the type of metal node, the type of linker and the topology of the MOF, which specifically modulate subtle variations in CT characteristics and electronic density delocalization, driven by changes in linker orientation and intermolecular coupling. Still, linker length, its electronic character and molecular flexibility also influence character of metal contributions and, thus, the 2PA response. Since H_4_TCPE is rather large and its optoelectronic response arises dominantly from the central part of the linker, we assume that the 2PA response in H_4_TCPE-based MOFs should originate mainly from the linker and its packing, rather than from the metal node. Secondly, experimental 2PA cross sections are often reported relative to reference dyes (for example, rhodamine or fluorescein standards) and therefore depend on the chosen calibration procedure, which we do not emulate computationally. Importantly, our results report *σ*_2_ rather than *ησ*_2_ meaning that we exclusively address the absorption process. The emission step, and therefore the fluorescence quantum yield, was not considered in this study. Third, the level of theory, DFT and TD-DFT as used here, as well as DFT functionals might impact the 2PA results (Beerepoot et al., 2015; Chołuj et al., 2022; Friese et al., 2011). In addition, our simulations treat individual optimized structures and rely on purely electronic vertical excitations, neglecting nuclear motion. In experiments, the measured response reflects an ensemble average over conformations and microenvironments and can be enhanced by vibronic and conformational contributions. In particular, we do not include Herzberg-Teller effects or vibronic coupling, which can activate otherwise weak transitions and modify intensities. Consequently, our absolute σ_2_ values are expected to be lower than experimentally measured cross sections in many cases. Next, the computed spectra are obtained using a Lorentzian broadening scheme (homogeneous broadening), which is a simplified line-shape model. Experimental spectra typically include additional broadening mechanisms, such as vibrational structure, solvent/temperature effects, and inhomogeneous broadening, and can vary substantially with the experimental setup (solution vs. solid state vs. MOF). Finally, for MOF-based measurements, uncertainties in light propagation within the material (e.g., penetration depth, scattering, and local field effects) further complicate the comparison between calculated molecular σ_2_ values and macroscopic experimental observables. Therefore, the agreement with experiment should be interpreted primarily in terms of relative trends rather than absolute magnitudes.

## 5 Conclusion

We performed quantum mechanical calculations of the H_4_TCPE linker to understand how intramolecular linker flexibility (d_C-C_) and intermolecular packing modulate its 2PA response. By combining qualitative EDD/orbital plots with quantitative analysis of CT parameters, we characterized the electronic character of the lowest 1PA and 2PA excitations and the most 2PA-active geometries of selected aggregate moieties. The initial monomer scan provided a reliable basis for selecting the optimal d_C-C_ distances for further packing studies, complementing experimental observations. Our results show that linker flexibility plays a significant role in modulating the excitation wavelength, with increased conjugation (greater planarity) leading to a red-shift in the absorption spectra. In addition, the two lowest singlet excitations exhibit complementary selection rules characteristic of a quadrupolar chromophore where the first excitation was 1PA allowed and 2PA forbidden, and the second excitation was 1PA forbidden but 2PA allowed. Extending the analysis to the dimeric conformations, we found that the 2PA response is maximized for arrangements that promote favorable relative orientation of the π-cores, where the enhanced 2PA response is the result of different effects such as i) charge redistribution (CT-like), ii) coherently coupled Frenkel-type excitons iii) and the isotropic polarizability. Notably, the dimeric systems with the most pronounced 2PA σ_2_ were the i) for opening angles not larger than 45°, ii) fully parallel stacking and iii) plane displacements not larger than −5 Å along the longer axis of the molecule. Indeed, some of those systems were experimentally achieved for Zn-H_4_TCPE MOF (Liu et al., 2022), or Zr-H_4_TCPE MOFs (He et al., 2025; Medishetty et al., 2017a) resulting in high 2PA response. However, so far fully parallel structures were not reported, opening further new design possibilities and enhanced NLO. Overall, this work demonstrates a structure-property relationship through a systematic computational scan of controlled H_4_TCPE-H_4_TCPE packing arrangements. In addition, both intramolecular flexibility and intermolecular packing were analyzed through EDD, orbital visualization and charge transfer analysis to understand how the geometrical changes modify the electronic structure and, consequently, the 2PA response.

## Data Availability

The raw data supporting the conclusions of this study are available in the NOMAD repository under https://doi.org/10.17172/NOMAD/2026.04.16-2.
