# Supplementary material for: Intermolecular packing effects on the two-photon absorption of the H4TCPE linker
Source: Front Chem. 2026 May 21;14:1805869. doi: 10.3389/fchem.2026.1805869 (PMC13233717; doi:10.3389/fchem.2026.1805869)
Supplement: Supplementary file 1 [file DataSheet1.pdf]

Supplementary Information

Intermolecular Packing Effects on the Two-Photon Absorption of the H_4TCPE linker

Helmy Pacheco Hernandez[1] and Mariana Kozlowska[1*]

[1]Institute of Nanotechnology (INT) Karlsruhe Institute of Technology (KIT) Kaiserstraße 12, 76131 Karlsruhe, Germany

Table of Contents

1 2PA calculations 2

2 Dihedral angle (monomer analysis) 3

3 Geometry optimization 4

4 Excitations of the H_4TCPE monomer for $d_{C\text{-}C}$= 13 - 19 Å. 5

5 1PA and 2PA spectra of the H_4TCPE monomer for $d_{C\text{-}C}$= 13 - 19 Å. 12

6 H_4TCPE dimer systems for $d_{C\text{-}C}$= 16 - 18 Å. 14

6.1 Book-opening angle 14

6.1.1 Short-Axis 14

6.1.2 Long-Axis 17

6.1.3 EDD and main orbital transitions: comparison of SA and LA at θ = 45° 20

6.1.4 Multiwfn analysis: comparison of SA and LA at θ = 45° 21

6.1.5 Polarizability results: SA and LA at θ = 45° 22

6.2 Stacking plane displacement: Δy 23

6.2.1 Displacement up: Δy= +5 Å 24

6.2.2 Displacement down: Δy= -5 Å 25

6.2.3 Displacement up: Δy= +10 Å 26

6.2.4 Displacement down: Δy= -10 Å 27

6.2.5 EDD and main orbital transitions: comparison of 4 Å vs 5 Å at Δy= -5 Å 28

6.2.6 Polarizability results: plane displacement Δy = 0 vs -5 Å displacement. 29

6.3 Stacking plane displacement: Δx 29

6.3.1 Displacement right: Δx= +5 Å 30

6.3.2 Displacement left: Δx= -5 Å 31
6.3.3 Displacement right: Δx= +10 Å 32
6.3.4 Displacement left: Δx= -10 Å 33
6.4 Plane rotation 34
6.4.1 Rotation angle: θ= 0° corresponds also to Δy=0 35
6.4.2 Rotation angle: θ= 30° 36
6.4.3 Rotation angle: θ= 60° 37
6.4.4 Rotation angle: θ= 90° 38
6.4.5 EDD and main orbital transitions: comparison of 4 Å vs 5 Å at θ= 0°. 39
6.4.6 Polarizability results: θ= 0° (or named Δy = 0) 40
7 References 41

1 2PA calculations

2PA transition tensors S_{ab} were computed using the quadratic-response implemented in TURBOMOLE[1] V7.7 (module escf). The invariants are defined from the 2PA transition tensor components S_{ab} (Cartesian components a, b = x, y, z) as:

$$dF = \frac{1}{30}\sum_{a,b} S_{aa}S_{bb}$$

$$dG = \frac{1}{30}\sum_{a,b} S_{ab}S_{ab}$$

$$dH = \frac{1}{30}\sum_{a,b} S_{ab}S_{ba}$$

S_{ab} are combined to yield rotationally averaged transition strengths for parallel, perpendicular or circular polarization as described in the following equations:

Parallel linearly polarized: $A_{\parallel} = 2dF + 2dG + 2dH$

Perpendicular linearly polarized: $A_{\perp} = -dF + 4dG - dH$

Circularly polarized: $A_{circ} = -2dF + 3dG + 3dH$

In our study, we considered parallel linearly polarized configuration to obtain the base cross section as described here:

Base cross section σ_0 is calculated as:

$$\sigma_0 = \frac{4\pi^3 \alpha a_0^5}{c} (\omega_1 \cdot \omega_2 \cdot S),$$

where:

α: is the fine-structure constant

a_0: Bohr radius

c: speed of light

ω_1 and ω_2: are the photon frequencies

S: transition strength in atomic units ($A_{\parallel}, A_{\perp}, or \ A_{circ}$)

Complete cross section is obtained by multiplying σ_0 with the line shape:

$$\sigma(\omega) = \sigma_0 \, g(2\omega)$$

Further details of the quadratic-response 2PA implementation and the definition of the printed tensor components S_{ab} are given in the TURBOMOLE 7.7 manual.

2 Dihedral angle (monomer analysis)

To understand the correlation between the observed shifts in the spectra (1PA and 2PA) with the structural modifications ($d_{C\text{-}C}$=13-20 Å), we analyzed the change in the phenyl-core twist of H_4TCPE across the monomer scan. We evaluated four equivalent torsion angles using VESTA[2], one for each inner phenyl ring directly bonded to the central C=C unit. For each ring, the dihedral angle (ω) was defined using an external ortho carbon on the phenyl ring as depicted in Figure S1, the corresponding ipso carbon, and the two alkene carbons of the central C=C core (C_{ortho}-C_{ipso}-C_{alkene}-C_{alkene}). The same external-ortho atom selection was applied consistently for all conformations (13-20 Å), the reported value corresponds to the average of the four torsions and it is shown in Table 1 in the main text.

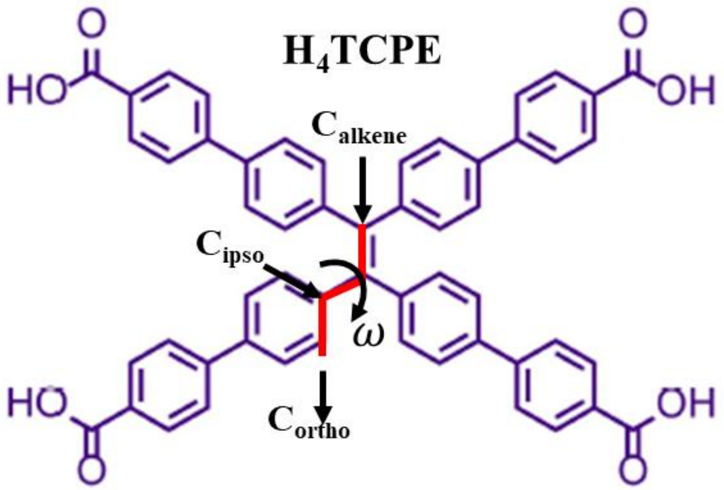

Figure S1. H_4TCPE linker. The four carbon atoms used to define the dihedral angle (ω) are highlighted in red.

3 Geometry optimization

Table S1. Comparison of final energies after geometry optimization. The relative energy between different structures was calculated as an energy difference with respect to the most stable structure characterized with the lowest absolute energy value.

d_{C-C} (Å)	GEO_OPT_Energy (Hartree)	GEO_OPT_Energy(kcal/mol)	$\Delta E_i = E_i - E_{min}$ (kcal/mol)
11	-2678.10118029	-1 680 508.49	28.26
12	-2678.11306060	-1 680 515.94	20.81
13	-2678.12369069	-1 680 522.61	14.14
14	-2678.13254771	-1 680 528.17	8.58
15	-2678.13917172	-1 680 532.33	4.42
16	-2678.14451787	-1 680 535.68	1.07
17	-2678.14622601	-1 680 536.75	0.00
18	-2678.14457408	-1 680 535.72	1.03
19	-2678.13700474	-1 680 530.97	5.78
20	-2678.11806517	-1 680 519.08	17.67

4 Excitations of the H_4TCPE monomer for d_{C-C}= 13 - 19 Å.

a)

13A					
Exc	1PA [nm]	2PA [nm]	Osc. Str.	2PA [GM]	Transition
1	293	586	1.908	0.117	HOMO->LUMO 75.3%
2	276	552	1.138	13.112	HOMO->LUMO+1 61.1%
3	274	549	0.190	84.226	HOMO->LUMO+2 59.1%
4	253	506	0.000	9.550	HOMO->LUMO+5 19%
5	251	503	0.002	151.439	HOMO->LUMO+3 30.6%
6	249	498	0.079	3.188	-
7	247	494	0.053	0.113	-
8	247	494	0.005	1.5917	-
9	246	492	0.088	4.993	-
10	246	492	0.045	0.142	-

b)

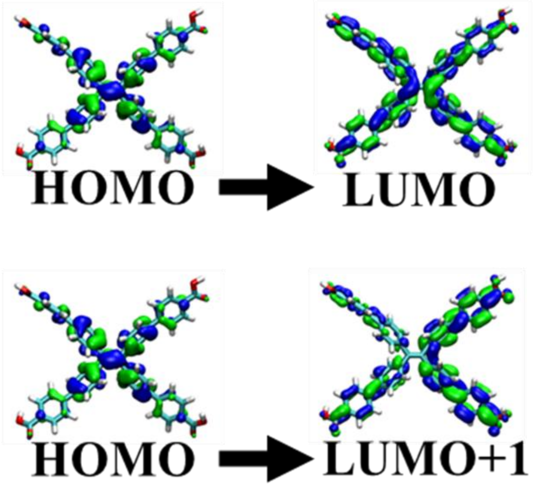

Figure S2. TD-DFT results for d_{C-C}= 13 Å. a) Computed 1PA and 2PA excitation wavelengths, oscillator strengths, and 2PA cross sections (σ_2, GM) for the ten lowest singlet excitations. b) Frontier molecular orbitals involved in the dominant transitions of the first and second excitations.

a)

14A					
**Exc**	**1PA [nm]**	**2PA [nm]**	**Osc. Str.**	**2PA [GM]**	**Transition**
1	304	609	1.640	0.217	HOMO->LUMO 80.1%
2	278	556	1.384	9.2671	HOMO->LUMO+1 63.8%
3	276	553	0.117	113.920	HOMO->LUMO+2 60.6%
4	256	513	0.000	9.280	HOMO->LUMO+5 38.7%
5	252	504	0.002	225.75	HOMO->LUMO+3 36.2%
6	249	499	0.101	2.234	-
7	248	496	0.012	0.783	-
8	247	494	0.010	1.687	-
9	246	493	0.078	9.053	-
10	246	492	0.034	0.904	-

b)

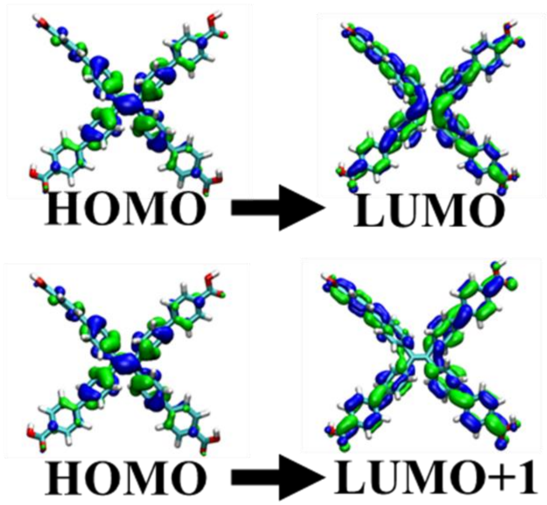

Figure S3. TD-DFT results for $d_{C\text{-}C}$= 14 Å. a) Computed 1PA and 2PA excitation wavelengths, oscillator strengths, and 2PA cross sections (σ_2, GM) for the lowest ten singlet states. b) Frontier molecular orbitals involved in the dominant transitions of the first and second excitations.

a)

15A					
Exc	1PA [nm]	2PA [nm]	Osc. Str.	2PA [GM]	Transition
1	314	628	1.406	0.298	HOMO-LUMO 82.5%
2	279	558	1.546	10.52	HOMO->LUMO+1 62.9%
3	278	557	0.132	121.3	HOMO->LUMO+2 58.4%
4	259	518	0.001	16.03	HOMO->LUMO+5 47.1%
5	253	506	0.002	291.0	HOMO->LUMO+3 39.8%
6	250	500	0.121	1.990	-
7	249	499	0.001	1.491	HOMO-4->LUMO 21.9%
8	247	494	0.052	12.56	-
9	247	494	0.054	13.90	-
10	246	492	0.018	1.193	-

b)

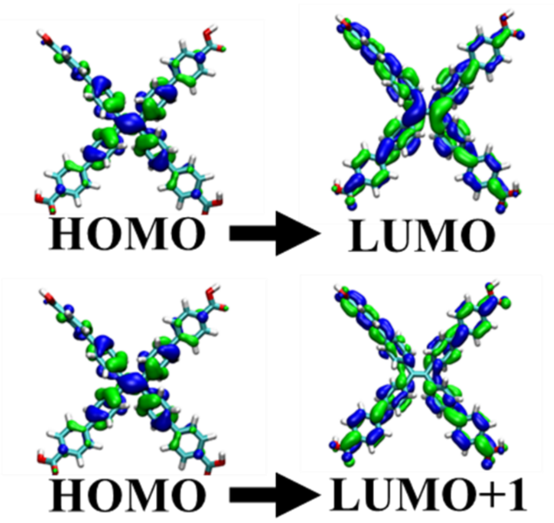

Figure S4. TD-DFT results for d_{C-C}= 15 Å. a) Computed 1PA and 2PA excitation wavelengths, oscillator strengths, and 2PA cross sections (σ_2, GM) for the lowest ten singlet states. b) Frontier molecular orbitals involved in the dominant transitions of the first and second excitations.

a)

16A					
**Exc**	**1PA [nm]**	**2PA [nm]**	**Osc. Str.**	**2PA [GM]**	**Transition**
1	325	650	1.136	0.331	HOMO->LUMO 84.5%
2	281	562	0.048	117.884	HOMO->LUMO+2 55.9%
3	280	561	1.850	3.818	HOMO->LUMO+1 63.1%
4	261	522	0.002	26.800	HOMO->LUMO+5 51.3%
5	254	508	0.002	369.926	HOMO->LUMO+3 44.2%
6	251	503	0.001	1.497	HOMO-4->LUMO 24.9%
7	251	502	0.176	2.681	-
8	247	495	0.050	40.064	-
9	247	495	0.064	44.168	-
10	246	492	0.011	3.680	-

b)

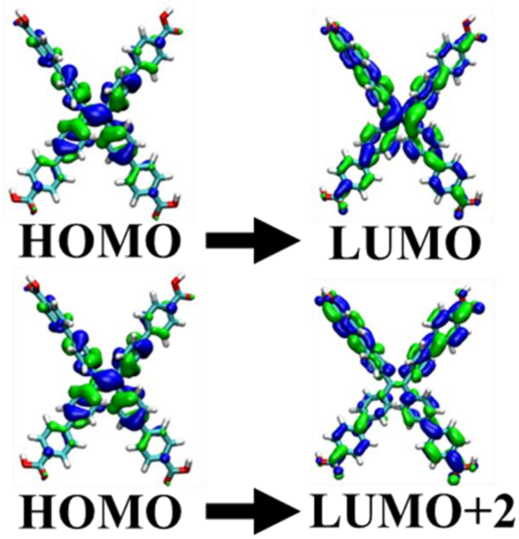

Figure S5. TD-DFT results for $d_{C\text{-}C}$= 16 Å. a) Computed 1PA and 2PA excitation wavelengths, oscillator strengths, and 2PA cross sections (σ_2, GM) for the lowest ten singlet states. b) Frontier molecular orbitals involved in the dominant transitions of the first and second excitations.

a)

17A					
Exc	1PA [nm]	2PA [nm]	Osc. Str.	2PA [GM]	Transition
1	334	668	0.908	0.310	HOMO->LUMO 85.8%
2	284	568	0.053	92.071	HOMO->LUMO+2 47.4%
3	282	564	2.027	3.510	HOMO->LUMO+1 57.5%
4	261	523	0.003	33.695	HOMO->LUMO+5 50.3%
5	254	509	0.004	431.06	HOMO->LUMO+3 48.1%
6	253	507	0.001	1.150	HOMO+4->LUMO 27.2%
7	252	504	0.286	1.953	-
8	248	497	0.062	406.483	HOMO-1->LUMO 28.8%
9	248	497	0.007	83.534	HOMO-7->LUMO 20.3%
10	247	494	0.080	240.13	-

b)

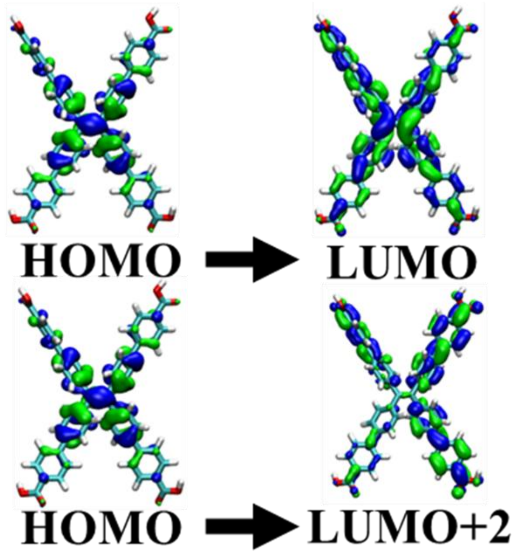

Figure S6. TD-DFT results for d_{C-C}= 17 Å. a) Computed 1PA and 2PA excitation wavelengths, oscillator strengths, and 2PA cross sections (σ_2, GM) for the lowest ten singlet states. b) Frontier molecular orbitals involved in the dominant transitions of the first and second excitations.

a)

18A					
Exc	1PA [nm]	2PA [nm]	Osc. Str.	2PA [GM]	Transition
1	340	681	0.713	0.281	HOMO->LUMO 86.5%
2	285	571	0.056	59.675	HOMO->LUMO+2 39.1%
3	282	564	2.158	2.809	HOMO->LUMO+1 52.2%
4	260	521	0.002	38.280	HOMO->LUMO+5 43.3%
5	255	510	0.009	437.94	HOMO->LUMO+3 49.6%
6	254	509	0.000	7.052	HOMO-4->LUMO 27.3%
7	253	506	0.445	2.363	HOMO-2->LUMO 20%
8	251	502	0.021	648.086	HOMO-1->LUMO 49.5%
9	249	498	0.001	59.036	HOMO-7->LUMO 24.4%
10	248	496	0.183	3.451	HOMO-2->LUMO 21.6%

b)

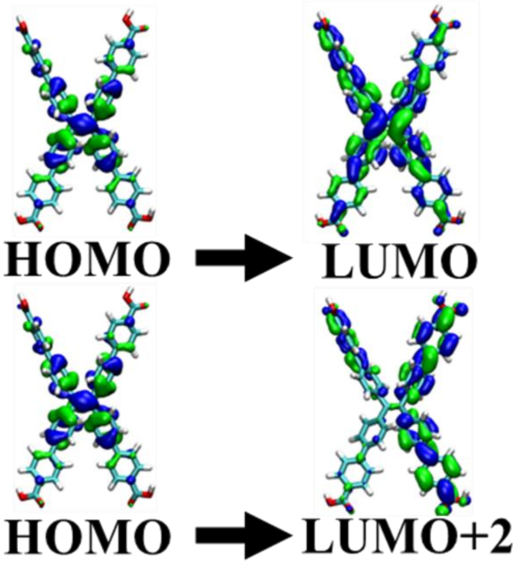

Figure S7. TD-DFT results for $d_{C\text{-}C}$= 18 Å. a) Computed 1PA and 2PA excitation wavelengths, oscillator strengths, and 2PA cross sections (σ_2, GM) for the lowest ten singlet states. b) Frontier molecular orbitals involved in the dominant transitions of the first and second excitations.

a)

19A					
Exc	1PA [nm]	2PA [nm]	Osc. Str.	2PA [GM]	Transition
1	335	671	0.451	0.267	HOMO->LUMO 85.3%
2	284	569	0.063	19.684	HOMO-1->LUMO 33.8% HOMO->LUMO+2 23.8%
3	278	557	2.222	1.400	HOMO-1->LUMO+1 38.9% HOMO->LUMO+2 26.7%
4	255	511	0.004	98.980	-
5	254	508	0.043	265.57	HOMO->LUMO+3 39.6%
6	253	507	0.546	10.047	-
7	252	504	0.000	7.026	HOMO-4->LUMO 20.4%
8	250	501	0.015	73.282	-
9	249	499	0.021	359.10	HOMO-1->LUMO 35.7%
10	247	495	0.083	6.605	-

b)

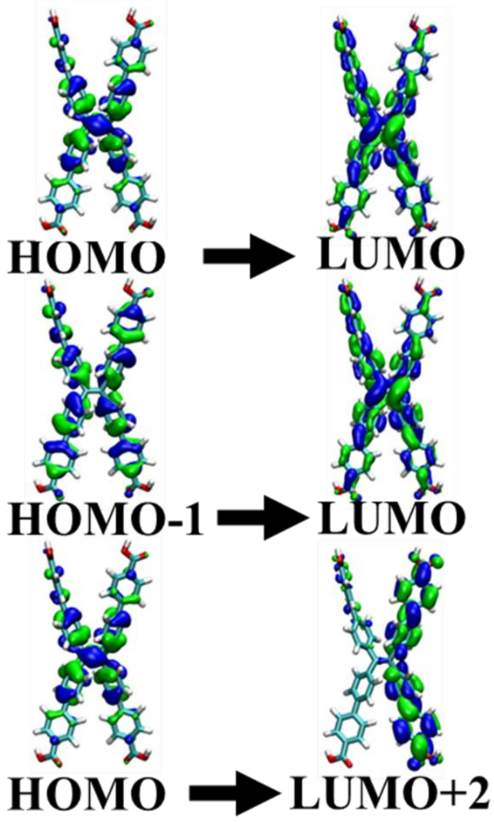

Figure S8. TD-DFT results for $d_{C\text{-}C}$= 19 Å. a) Computed 1PA and 2PA excitation wavelengths, oscillator strengths, and 2PA cross sections (σ_2, GM) for the lowest ten singlet states. b) Frontier molecular orbitals involved in the dominant transitions of the first and second excitations.

5 1PA and 2PA spectra of the H_4TCPE monomer for d_{C-C}= 13 - 19 Å.

1PA and 2PA spectra were plotted using half width at half maximum (HWHM) of 0.1 eV and Lorentzian type broadening function.

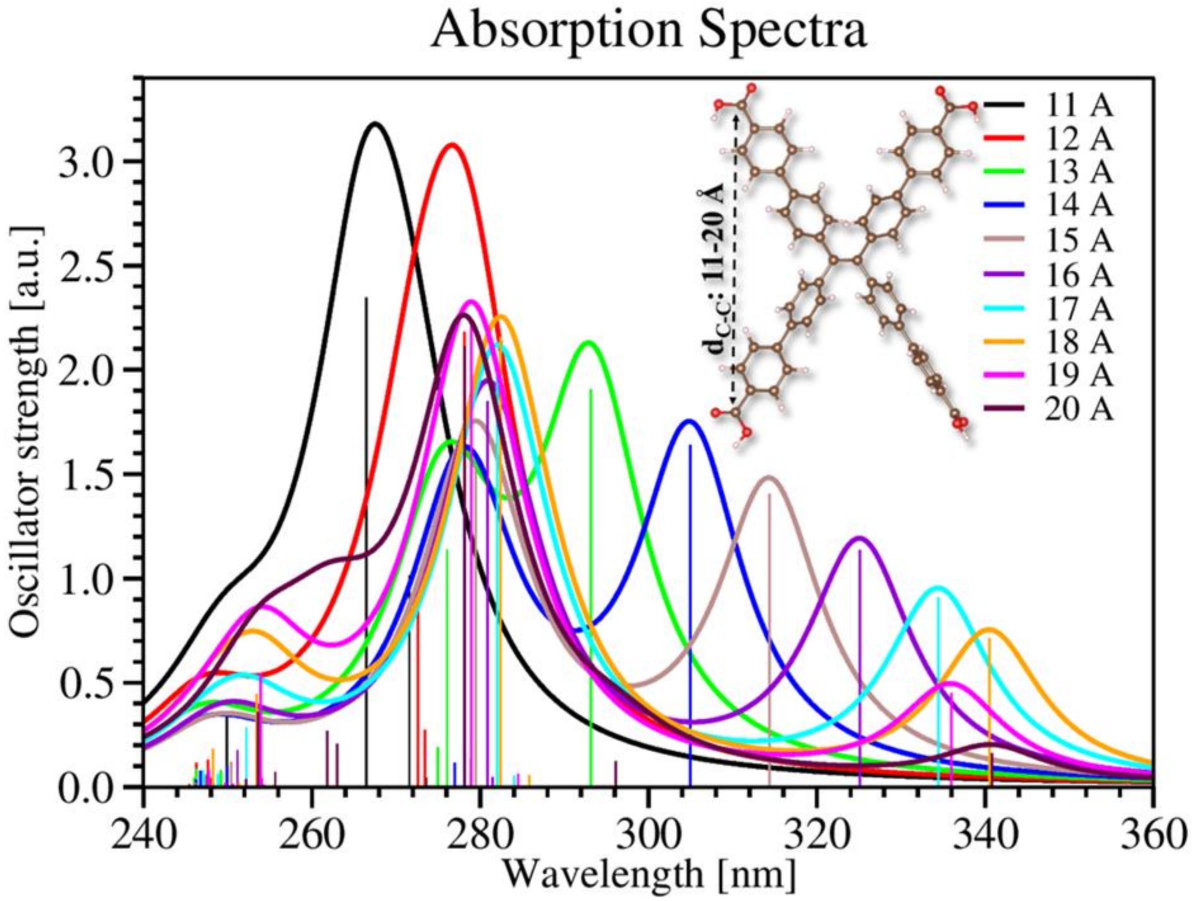

Figure S9. Absorption spectra for the H_4TCPE monomer with C-C distance scan (see Figure 1, 3). Although spectra are shown for d_{C-C}= 11-20 Å, subsequent analysis focuses on d_{C-C}=13-19 Å, since the 11, 12, and 20 Å structures correspond to overcompressed conformations.

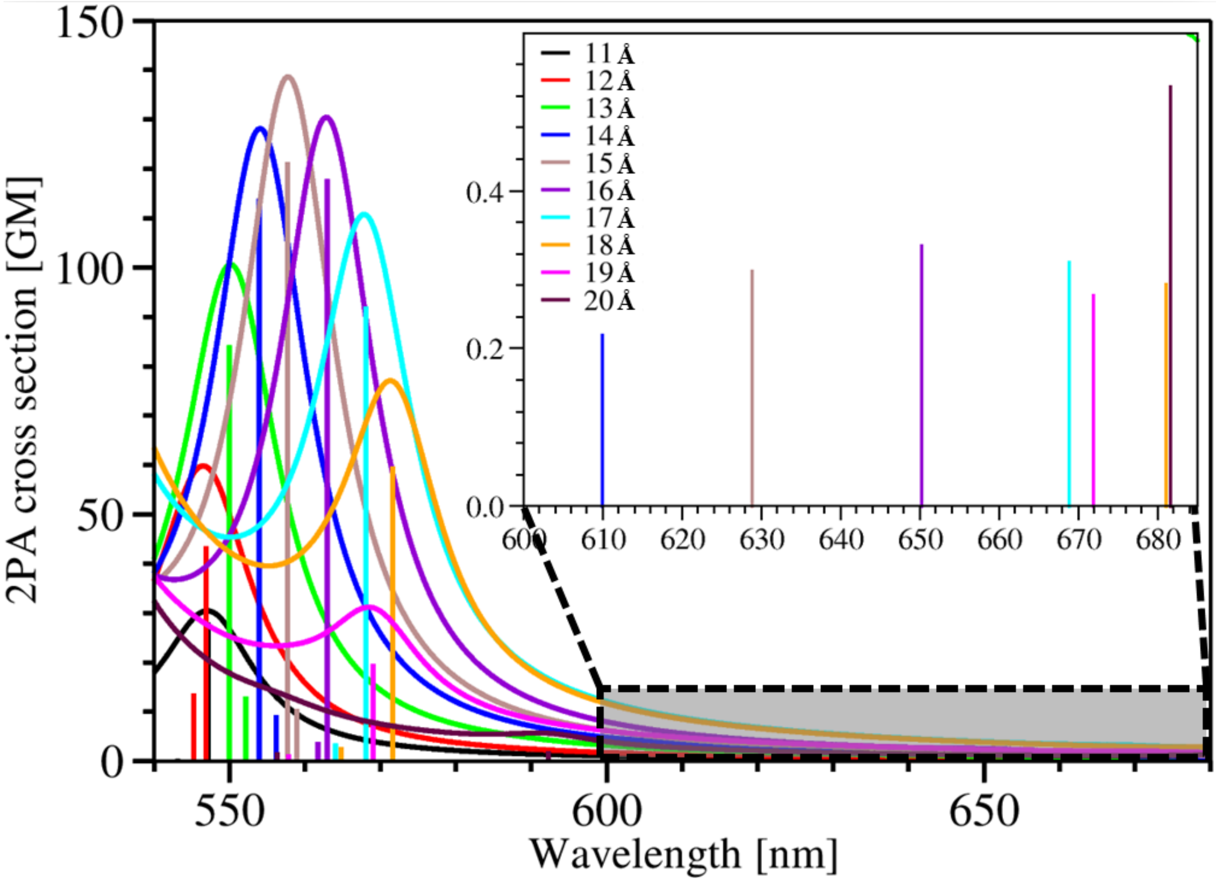

Figure S10. 2PA spectra for the H_4TCPE monomer distance scan (d_{C-C} = 11-20 Å) . The main panel shows σ_2 over 540-680 nm, while the inset provides a zoom of the low-intensity region from 600-690 nm. Analysis focuses on d_{C-C}=13-19 Å, since the 11, 12, and 20 Å structures correspond to overcompressed conformations.

6 H_4TCPE dimer systems for $d_{C\text{-}C}$= 16 - 18 Å.

6.1 Book-opening angle

6.1.1 Short-Axis

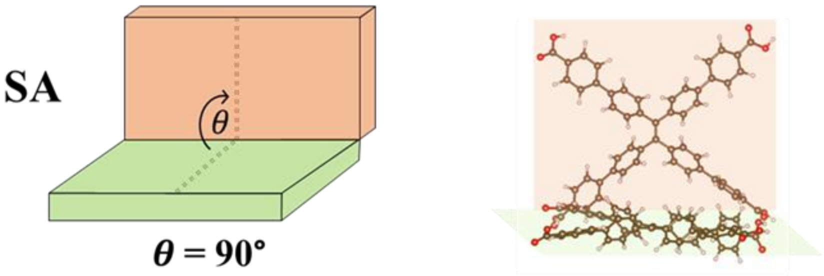

Figure S11. Schematic representation of the opening angle between short axis of H_4TCPE.

Table S2. TD-DFT results for the H_4TCPE dimer at SA ϑ = 45° (stacking distance = 4 Å) for d_{C-C}=16-18 Å, including 1PA/2PA wavelengths, oscillator strengths, 2PA cross sections (σ_2) and dominant orbital transitions.

SA θ = 45° (stacking 4 Å)															
	16A					17A					18A				
Exc	1PA [nm]	2PA [nm]	Osc. Str.	2PA [GM]	Transition	1PA [nm]	2PA [nm]	Osc. Str.	2PA [GM]	Transition	1PA [nm]	2PA [nm]	Osc. Str.	2PA [GM]	Transition
1	326	653	0.683	8.288	HOMO-1->LUMO (62.5%) HOMO->LUMO+1 (11.3%)	332	664	0.589	8.245	HOMO-1->LUMO (64.5%) HOMO->LUMO+1 (10.9%)	335	671	0.507	7.720	HOMO-1->LUMO (66.5%) HOMO->LUMO+1 (9.2%)
2	320	640	1.508	1.685	HOMO->LUMO+1 (70.1%) HOMO-1->LUMO (10.5%)	325	651	1.181	1.360	HOMO->LUMO+1 (72.7%) HOMO-1->LUMO (10.1%)	329	658	0.880	1.038	HOMO->LUMO+1 (75.2%) HOMO-1->LUMO (8.4%)
3	283	566	0.087	57.988	HOMO->LUMO+3 (24.5%)	284	568	0.051	66.087	HOMO-1->LUMO+2 (28.7%)	284	569	0.046	61.774	HOMO-1->LUMO+2 (29.5%)
4	280	560	0.017	23.056	HOMO->LUMO+4 (16.3%)	280	561	0.048	28.901	HOMO->LUMO+3 (25.9%)	281	562	0.095	46.593	HOMO->LUMO+3 (23.5%)
5	279	558	0.943	107.983	HOMO-1->LUMO+4 (19.8%)	280	560	0.442	75.211	HOMO-1->LUMO+4 (28.9%)	280	561	0.144	21.696	HOMO-1->LUMO+4 (34.3%)
6	276	553	2.303	66.912	HOMO-1->LUMO+2 (30.6%)	277	554	3.051	35.138	HOMO->LUMO+3 (25.1%)	277	554	3.421	22.781	HOMO->LUMO+3 (26.8%)
7	260	521	0.007	35.405	HOMO->LUMO+11 (39%)	264	528	0.001	3.373	HOMO->LUMO (86.7%)	267	535	0.004	3.355	HOMO->LUMO (20.4%)
8	259	518	0.003	23.945	HOMO->LUMO+12 (45.8%)	260	520	0.016	39.522	HOMO-1->LUMO+11 (29%)	259	518	0.041	35.907	HOMO-1->LUMO+11 (29%)
9	257	514	0.000	5.614	HOMO->LUMO (84%)	259	518	0.005	27.234	HOMO->LUMO+12 (42.4%)	258	517	0.008	25.501	HOMO->LUMO+12 (31.8%)
10	255	510	0.020	59.255	HOMO->LUMO+6 (17.6%)	255	511	0.024	34.982	HOMO->LUMO+6 (14.5%)	256	512	0.027	24.844	HOMO->LUMO+6 (11.2%)

Table S3. TD-DFT results for the H_4TCPE dimer at SA ϑ = 90° (stacking distance = 4 Å) for d_{C-C}=16-18 Å, including 1PA/2PA wavelengths, oscillator strengths, 2PA cross sections (σ_2), and dominant orbital transitions.

SA θ = 90° (stacking 4 Å)															
	16A					17A					18A				
Exc	1PA [nm]	2PA [nm]	Osc. Str.	2PA [GM]	Transition	1PA [nm]	2PA [nm]	Osc. Str.	2PA [GM]	Transition	1PA [nm]	2PA [nm]	Osc. Str.	2PA [GM]	Transition
1	330	660	1.222	3.056	HOMO->LUMO+1 (66.8%)	339	679	0.979	2.376	HOMO->LUMO+1 (67%)	345	691	0.775	1.688	HOMO->LUMO (81%)
2	320	640	1.156	1.094	HOMO-1->LUMO (69.9%)	327	654	0.926	0.951	HOMO-1->LUMO (68.4%)	329	659	0.725	0.649	HOMO-1->LUMO+1 (80.8%)
3	287	575	0.026	72.095	HOMO-1->LUMO+2 (28%)	289	579	0.023	61.259	HOMO->LUMO+3 (27.4%)	290	581	0.047	46.757	HOMO->LUMO+3 (34%)
4	282	564	0.408	90.798	HOMO-1->LUMO+2 (27.4%)	284	569	0.235	73.168	HOMO-1->LUMO+2 (27.7%)	285	570	0.027	39.625	HOMO-1->LUMO+2 (30%)
5	281	562	1.699	35.910	HOMO->LUMO+5 (42.1%)	282	565	1.635	28.773	HOMO->LUMO+5 (37.5%)	282	565	1.537	22.808	HOMO->LUMO+5 (28%)
6	278	557	1.355	41.540	HOMO-1->LUMO+4 (59.4%)	279	558	1.875	22.630	HOMO-1->LUMO+4 (54.5%)	279	558	2.339	10.088	HOMO-1->LUMO+4 (48.1%)
7	262	524	0.002	32.122	HOMO->LUMO+11 (36%)	262	525	0.005	36.027	HOMO->LUMO+11 (32.9%)	261	523	0.007	33.890	HOMO->LUMO+12 (27.9%)
8	259	519	0.012	21.406	HOMO-1->LUMO+10 (12.1%)	260	520	0.029	29.429	HOMO-1->LUMO+12 (10.8%)	258	517	0.047	37.466	HOMO-1->LUMO+13 (19.2%)
9	256	512	0.015	87.811	HOMO-1->LUMO+6 (19%)	256	513	0.034	110.28	HOMO->LUMO+7 (22.3%)	257	515	0.063	171.389	HOMO->LUMO+7 (28.2%)
10	254	509	0.014	491.95	HOMO-1->LUMO+6 (17.2%)	255	511	0.010	190.43	-	256	512	0.047	40.108	-

Table S4. TD-DFT results for the H_4TCPE dimer at SA ϑ = 135° (stacking distance = 4 Å) for d_{C-C}=16-18 Å, including 1PA/2PA wavelengths, oscillator strengths, 2PA cross sections (σ_2), and dominant orbital transitions.

SA θ = 135° (stacking 4 Å)																	
**Exc**		**1PA [nm]**	**2PA [nm]**	**Osc. Str.**	**2PA [GM]**	**Transition**		**1PA [nm]**	**2PA [nm]**	**Osc. Str.**	**2PA [GM]**	**Transition**		**1PA [nm]**	**2PA [nm]**	**Osc. Str.**	**2PA [GM]**	**Transition**
1		330	661	1.461	1.261	HOMO->LUMO+1 (79.6%)		339	678	1.266	0.657	HOMO->LUMO+1 (82.2%)		338	677	0.830	1.207	HOMO->LUMO (78.9%)
2		319	639	0.966	1.558	HOMO-1->LUMO (75.6%)		332	665	0.637	1.493	HOMO-1->LUMO (79.6%)		315	631	0.711	1.662	HOMO-1->LUMO+1 (75.1%)
3		286	572	0.080	110.05	HOMO->LUMO+3 (23%)		289	579	0.036	102.03	HOMO-1->LUMO+2 (39.2%)		291	583	0.302	61.758	HOMO->LUMO+2 (45.9%)
4		281	563	0.587	52.804	HOMO-1->LUMO+5 (55.4%)		284	569	0.017	27.624	HOMO->LUMO+5 (37.8%)		282	565	0.947	25.079	HOMO->LUMO+4 (52.3%)
5	**16A**	279	558	0.181	85.767	HOMO-1->LUMO+2 (24.1%)	**17A**	282	565	0.199	51.103	HOMO->LUMO+4 (32.3%)	**18A**	277	554	0.457	39.045	HOMO-1->LUMO+5 (54.6%)
6		278	556	2.612	9.893	HOMO-1->LUMO+4 (30.1%)		281	562	3.478	2.426	HOMO-1->LUMO+3 (30.6%)		274	549	2.235	12.889	HOMO-1->LUMO+3 (47.3%)
7		263	526	0.003	23.531	HOMO->LUMO+11 (30.3%)		263	527	0.004	22.939	HOMO->LUMO+11 (31.3%)		261	523	0.006	20.125	HOMO->LUMO+11 (26.6%)
8		260	520	0.003	17.650	HOMO-1->LUMO+10 (51.9%)		261	522	0.003	27.477	HOMO-1->LUMO+10 (30.3%)		258	516	0.254	216.19	HOMO->LUMO+6 (29%)
9		255	511	0.030	205.877	HOMO->LUMO+7 (29.2%)		257	514	0.052	147.56	HOMO-1->LUMO+6 (36.1%)		256	513	0.003	38.641	HOMO-1->LUMO+12 (29.7%)
10		253	507	0.029	5.390	HOMO->LUMO+21 (15.2%)		255	511	0.016	292.11	HOMO->LUMO+7 (13%)		256	512	0.240	82.378	HOMO-7->LUMO (13.4%)

Table S5. TD-DFT results for the H_4TCPE dimer at SA ϑ = 180° (stacking distance = 4 Å) for d_{C-C}=16-18 Å, including 1PA/2PA wavelengths, oscillator strengths, 2PA cross sections (σ_2), and dominant orbital transitions.

SA θ = 180°(stacking 4 Å)																	
**Exc**		**1PA [nm]**	**2PA [nm]**	**Osc. Str.**	**2PA [GM]**	**Transition**		**1PA [nm]**	**2PA [nm]**	**Osc. Str.**	**2PA [GM]**	**Transition**		**1PA [nm]**	**2PA [nm]**	**Osc. Str.**	**2PA [GM]**	**Transition**
1		323	647	1.906	2.253	HOMO-1->LUMO (76.2%)		337	675	1.891	0.695	HOMO-1->LUMO (45.4%)		340	681	1.359	2.403	HOMO-1->LUMO (69.4%)
2		319	638	0.634	0.610	HOMO->LUMO+1 (76.8%)		334	669	0.057	2.542	HOMO->LUMO+1 (52.8%)		337	675	0.205	0.324	HOMO->LUMO+1 (72.2%)
3		283	567	0.111	79.574	HOMO->LUMO+6 (28.9%)		289	578	0.015	116.37	HOMO->LUMO+2 (15.9 %)		288	577	0.095	90.196	HOMO-1->LUMO+2 (40.8%)
4		279	559	0.589	67.043	HOMO-1->LUMO+3 (60.9%)		283	567	0.000	4.579	HOMO-1->LUMO+4 (15.2%)		284	568	0.010	11.860	HOMO->LUMO+5 (28.8%)
5	**16A**	278	557	0.425	44.329	HOMO->LUMO+4 (48.7%)	**17A**	282	564	0.026	60.061	HOMO->LUMO+5 (27.1%)	**18A**	282	565	0.081	28.806	HOMO-1->LUMO+4 (34.9%)
6		276	553	2.337	24.229	HOMO-1->LUMO+2 (43.8%)		280	560	3.710	2.520	HOMO->LUMO+5 (15.7%)		280	561	3.833	11.54	HOMO->LUMO+3 (36.3%)
7		260	520	0.004	27.703	HOMO->LUMO+11 (19.2%)		262	524	0.010	56.907	HOMO->LUMO+12 (18.4%)		260	521	0.012	55.078	HOMO-1->LUMO+12 (18.1%)
8		260	520	0.005	19.935	HOMO-1->LUMO+9 (46.4%)		262	524	0.002	1.597	HOMO-1->LUMO+11 (24.5%)		260	520	0.004	26.342	HOMO->LUMO+12 (15.2%)
9		255	511	0.037	67.897	HOMO->LUMO+7 (24.2%)		256	513	0.007	11.004	-		256	512	0.010	175.20	HOMO-1->LUMO+6 (39.8%)
10		253	507	0.088	15.446	-		255	510	0.005	735.89	-		254	509	0.016	650.20	HOMO->LUMO+7 (39.8%)

6.1.2 Long-Axis

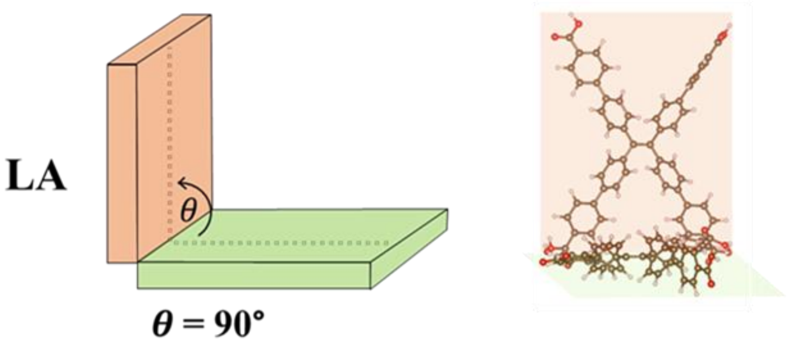

Figure S12. Schematic representation of the opening angle between long axis of H_4TCPE.

Table S6. TD-DFT results for the H_4TCPE dimer at LA ϑ = 45° (stacking distance = 4 Å) for d_{C-C}=16-18 Å, including 1PA/2PA wavelengths, oscillator strengths, 2PA cross sections (σ_2), and dominant orbital transitions.

LA θ = 45°(stacking 4 Å)																		
**Exc**		**1PA [nm]**	**2PA [nm]**	**Osc. Str.**	**2PA [GM]**	**Transition**		**1PA [nm]**	**2PA [nm]**	**Osc. Str.**	**2PA [GM]**	**Transition**		**1PA [nm]**	**2PA [nm]**	**Osc. Str.**	**2PA [GM]**	**Transition**
1		327	654	0.101	11.299	HOMO->LUMO+1 (50.3%) HOMO-1->LUMO (22.8%)		338	677	0.034	10.106	HOMO->LUMO+1 (47.4%) HOMO-1->LUMO (29.2%)		341	683	0.065	5.339	HOMO-1->LUMO (54.7%) HOMO->LUMO+1 (24.4%)
2		322	644	2.0025	5.521	HOMO-1->LUMO (37.4%) HOMO->LUMO+1 (30.7%)		333	667	1.676	3.981	HOMO-1->LUMO (38.3%) HOMO->LUMO+1 (36.2%)		337	675	1.273	1.040	HOMO->LUMO+1 (60.7%) HOMO-1->LUMO (22.1%)
3		289	578	0.511	109.68	HOMO-1->LUMO+2 (48.7%)		292	584	0.488	106.99	HOMO-1->LUMO+2 (47.1%)		290	581	0.354	65.568	HOMO-1->LUMO+2 (44.6%)
4	**16A**	280	561	0.126	81.701	HOMO->LUMO+3 (44.1%)	**17A**	287	575	0.443	21.844	HOMO->LUMO+3 (21.6%)	**18A**	286	573	0.347	39.290	HOMO->LUMO+4 (32.6%)
5		278	557	1.538	13.937	HOMO->LUMO+4 (41.0%)		284	568	0.469	71.731	HOMO-1->LUMO+4 (17.4%)		284	569	0.315	26.770	HOMO-1->LUMO+3 (31%)
6		272	545	1.408	38.522	HOMO-1->LUMO+5 (47.6%)		281	562	2.572	10.631	HOMO->LUMO+5 (36.5%)		281	563	3.221	8.890	HOMO->LUMO+4 (24.9%)
7		263	527	0.002	6.451	HOMO->LUMO (73.8%)		273	546	0.000	3.441	HOMO->LUMO (80.7%)		262	525	0.002	3.058	HOMO->LUMO (81%)
8		260	521	0.004	26.964	HOMO->LUMO+12 (41.8%)		261	523	0.007	32.437	HOMO->LUMO+12 (22.8%)		261	522	0.014	57.436	HOMO-1->LUMO+10 (28.7%)
9		260	520	0.044	62.824	HOMO-1->LUMO+13 (31%)		261	523	0.007	90.552	HOMO-1->LUMO+11 (16.7%)		260	521	0.002	46.92	HOMO->LUMO+12 (25.5%)
10		255	510	0.015	59.646	-		259	518	0.060	115.71	HOMO-1->LUMO+6 (27.7%)		258	516	0.057	137.97	HOMO-1->LUMO+6 (22.1%)

Table S7. TD-DFT results for the H_4TCPE dimer at LA ϑ = 90° (stacking distance = 4 Å) for d_{C-C}=16-18 Å, including 1PA/2PA wavelengths, oscillator strengths, 2PA cross sections (σ_2), and dominant orbital transitions.

LA θ = 90°(stacking 4 Å)																		
**Exc**		**1PA [nm]**	**2PA [nm]**	**Osc. Str.**	**2PA [GM]**	**Transition**		**1PA [nm]**	**2PA [nm]**	**Osc. Str.**	**2PA [GM]**	**Transition**		**1PA [nm]**	**2PA [nm]**	**Osc. Str.**	**2PA [GM]**	**Transition**
1		329	658	0.440	2.383	HOMO-1->LUMO (74.6%)		341	682	0.462	2.598	HOMO->LUMO+2 (79.2%)		346	693	0.505	1.470	HOMO->LUMO+2 (84.1%)
2		323	647	1.574	6.956	HOMO->LUMO+3 (72.6%)		334	668	1.189	5.021	HOMO-1->LUMO (73.9%)		334	668	0.838	3.811	HOMO-1->LUMO (77%)
3		285	570	1.063	53.896	HOMO->LUMO+5 (42.3%)		290	581	1.058	82.935	HOMO-1->LUMO+1 (33.4%)		288	577	0.495	62.818	HOMO-1->LUMO+3 (26.7%)
4		280	561	0.502	90.888	HOMO-1->LUMO+2 (31.5%)		287	575	0.790	61.395	HOMO->LUMO+5 (33.2%)		286	573	0.644	54.287	HOMO-1->LUMO+3 (24.8%)
5	**16A**	277	555	1.420	37.572	HOMO-1->LUMO+1 (29%)	**17A**	278	557	1.200	61.240	HOMO-1->LUMO+3 (34.7%)	**18A**	285	570	1.948	39.908	HOMO-1->LUMO+1 (43.5%)
6		273	547	0.737	80.446	HOMO->LUMO+6 (30.2%)		276	552	0.976	84.064	HOMO->LUMO+6 (30%)		278	557	1.358	42.769	HOMO->LUMO+5 (33.3%)
7		261	523	0.015	27.602	HOMO-1->LUMO+11 (27.8%)		269	538	0.001	3.060	HOMO->LUMO (87.9%)		263	526	0.000	3.425	HOMO->LUMO (88.2%)
8		260	521	0.039	33.963	HOMO->LUMO+13 (42.6%)		263	527	0.025	30.576	HOMO->LUMO+13 (33.8%)		262	524	0.01	14.643	HOMO->LUMO+14 (39%)
9		257	515	0.000	0.5799	HOMO->LUMO (86.8%)		261	523	0.029	39.358	HOMO-1->LUMO+8 (22.8%)		259	519	0.001	41.948	HOMO-1->LUMO+11 (24.6%)
10		253	507	0.064	44.306	-		256	512	0.030	285.19	HOMO-1->LUMO+4 (29.6%)		257	514	0.012	299.1	HOMO-1->LUMO+4 (38.4%)

Table S8. TD-DFT results for the H_4TCPE dimer at LA ϑ = 135° (stacking distance = 4 Å) for $d_{C\text{–}C}$=16-18 Å, including 1PA/2PA wavelengths, oscillator strengths, 2PA cross sections (σ_2), and dominant orbital transitions.

LA θ = 135°(stacking 4 Å)																				
**Exc**		**1PA [nm]**	**2PA [nm]**	**Osc. Str.**	**2PA [GM]**	**Transition**		**1PA [nm]**	**2PA [nm]**	**Osc. Str.**	**2PA [GM]**	**Transition**		**1PA [nm]**	**2PA [nm]**	**Osc. Str.**	**2PA [GM]**	**Transition**
1		326	653	0.360	2.639	HOMO->LUMO+1 (72.5%)		335	671	0.417	5.893	HOMO->LUMO+1 (77%)		341	682	0.021	11.051	HOMO->LUMO+1 (50.3%)
2		322	644	1.833	1.148	HOMO-1->LUMO (71.1%)		331	663	1.368	3.687	HOMO-1->LUMO (71.2%)		338	677	1.34	0.708	HOMO-1->LUMO (48.3%)
3		285	570	1.297	106.39	HOMO-1->LUMO+2 (56.6%)		287	575	0.184	177.02	HOMO-1->LUMO+2 (33.6%)		290	581	0.216	145.13	HOMO-1->LUMO+2 (37.5%)
4		284	568	1.389	76.973	HOMO->LUMO+3 (59%)		286	573	1.886	18.074	HOMO->LUMO+3 (42.8%)		289	578	1.626	15.374	HOMO->LUMO+3 (39.6%)
5	**16A**	280	560	0.894	23.861	HOMO->LUMO+5 (47.2%)	**17A**	283	567	1.989	14.826	HOMO-1->LUMO+4 (27.2%)	**18A**	284	568	2.263	8.243	HOMO->LUMO+5 (34.3%)
6		278	557	0.688	26.228	HOMO-1->LUMO+4 (52.2%)		281	563	0.636	4.315	HOMO-1->LUMO+4 (25.2%)		280	561	1.002	8.752	HOMO-1->LUMO+4 (41.4%)
7		261	522	0.009	26.839	HOMO->LUMO+11 (44.2%)		261	523	0.007	47.835	HOMO->LUMO+12 (27%)		261	522	0.017	135.81	HOMO-1->LUMO+12 (24.5%)
8		260	521	0.006	21.664	HOMO-1->LUMO+8 (25.5%)		261	522	0.002	51.774	HOMO-1->LUMO+10 (34.2%)		260	521	0.012	27.671	HOMO->LUMO+13 (21.6%)
9		255	510	0.012	49.365	HOMO-1->LUMO+6 (22.1%)		257	514	0.004	96.491	HOMO-1->LUMO+6 (22.4%)		258	516	0.055	198.48	HOMO->LUMO+5 (19.5%)
10		254	508	0.012	581.34	HOMO->LUMO+7 (24.7%)		256	512	0.054	624.16	-		256	513	0.013	632.04	HOMO-1->LUMO+6 (17.1%)

Table S9. TD-DFT results for the H_4TCPE dimer at LA ϑ = 180° (stacking distance = 4 Å) for $d_{C\text{–}C}$=16-18 Å, including 1PA/2PA wavelengths, oscillator strengths, 2PA cross sections (σ_2), and dominant orbital transitions.

LA θ = 180°(stacking 4 Å)																				
**Exc**		**1PA [nm]**	**2PA [nm]**	**Osc. Str.**	**2PA [GM]**	**Transition**		**1PA [nm]**	**2PA [nm]**	**Osc. Str.**	**2PA [GM]**	**Transition**		**1PA [nm]**	**2PA [nm]**	**Osc. Str.**	**2PA [GM]**	**Transition**
1		325	651	0.422	2.960	HOMO-1->LUMO (70%)		335	670	0.308	0.220	HOMO->LUMO+1 (72.7%)		339	679	0.009	0.401	HOMO-1->LUMO (44.6%)
2		320	641	1.750	1.811	HOMO->LUMO+1 (73.8%)		331	662	1.486	2.610	HOMO-1->LUMO (70.7%)		337	675	1.337	0.182	HOMO->LUMO+1 (43.3%)
3		284	568	1.032	183.38	HOMO-1->LUMO+2 (31.8%)		291	583	1.786	109.22	HOMO-1->LUMO+2 (50.1%)		285	571	0.084	108.51	HOMO-1->LUMO+4 (19.7%)
4		282	565	3.223	41.128	HOMO->LUMO+4 (33.4%)		286	573	0.109	42.304	HOMO->LUMO+3 (46%)		283	567	0.002	1.306	HOMO->LUMO+5 (18.2%)
5	**16A**	280	561	0.155	5.643	HOMO->LUMO+5 (30.5%)	**17A**	283	566	2.880	44.115	HOMO->LUMO+5 (40.2%)	**18A**	283	566	4.931	3.725	HOMO-1->LUMO+2 (24.9%)
6		279	558	0.028	3.094	HOMO-1->LUMO+3 (34.8%)		280	560	0.123	6.713	HOMO-1->LUMO+4 (42.7%)		280	560	0.010	16.294	HOMO-1->LUMO+2 (23.9%)
7		261	522	0.001	43.476	HOMO-1->LUMO+9 (49%)		261	523	0.018	55.082	HOMO-1->LUMO+10 (22.6%)		260	521	0.004	59.732	HOMO-1->LUMO+10 (22.4%)
8		260	520	0.006	15.474	HOMO->LUMO+12 (37.3%)		261	523	0.008	24.253	HOMO->LUMO+11 (28.2%)		260	521	0.001	1.652	HOMO-1->LUMO+10 (21.7%)
9		256	512	0.005	24.944	HOMO->LUMO+7 (19.7%)		258	516	0.066	58.917	HOMO-1->LUMO+6 (19.8%)		255	510	0.077	0.492	-
10		255	510	0.011	517.29	HOMO-1->LUMO+6 (27.7%)		256	512	0.036	15.349	-		254	509	0.002	253.46	-

6.1.3 EDD and main orbital transitions: comparison of SA and LA at θ = 45°

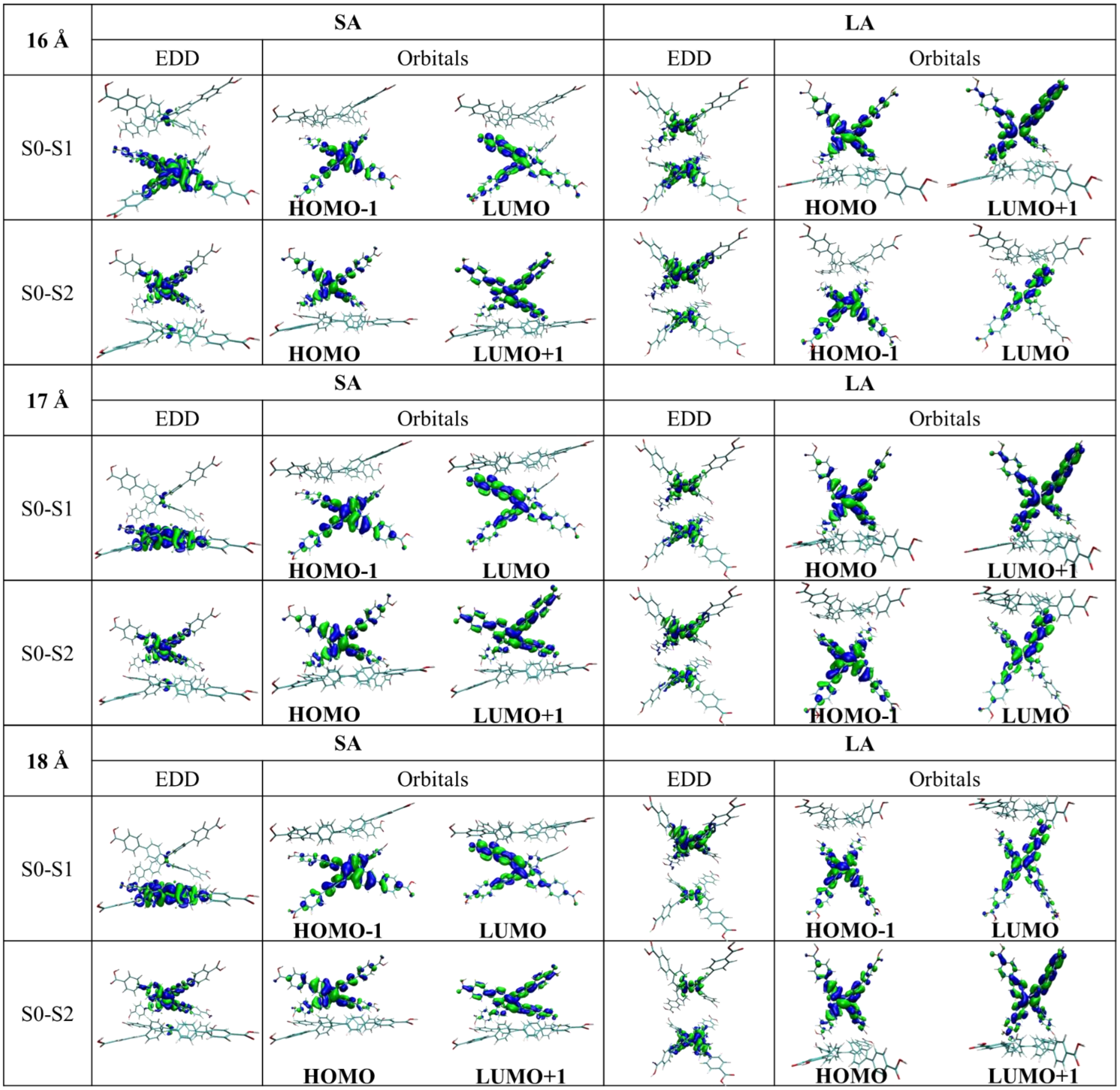

Figure S13. Comparison of short- and long-axis configurations for d_{C-C}= 16-18 Å. EDD plots and dominant orbital transitions for the first and second excitations.

6.1.4 Multiwfn analysis: comparison of SA and LA at θ = 45°

Table S10. Electronic character of the first and second excitations across the SA and LA configurations for $d_{C\text{-}C}$= 16-18 Å. The parameters calculated are described in detail in the main body (section 3.1).

Structure	$d_{C\text{-}C}$ (Å)	State	Sr (au)	D (Å)	Variation of dipole moment with respect to ground state (a.u.)	H_{CT} (Å)	H (Å)	t (Å)
SA	16	S0-S1	0.68	1.36	2.232	3.31	5.50	-1.95
		S0-S2	0.65	0.73	1.175	3.21	5.51	-2.47
	17	S0-S1	0.69	1.40	2.336	3.35	5.26	-1.94
		S0-S2	0.68	0.32	0.536	3.15	5.24	-2.83
	18	S0-S1	0.70	1.50	2.509	3.39	5.01	-1.89
		S0-S2	0.69	0.20	0.340	3.16	5.00	-2.95
LA	16	S0-S1	0.63	0.67	1.053	3.46	6.61	-2.78
		S0-S2	0.66	0.62	1.006	3.40	6.59	-2.78
	17	S0-S1	0.64	0.50	0.799	4.00	6.90	-3.50
		S0-S2	0.65	0.59	0.953	3.81	6.91	-3.21
	18	S0-S1	0.71	0.53	0.893	3.63	6.69	-3.10
		S0-S2	0.70	0.31	0.532	3.76	6.73	-3.44

6.1.5 Polarizability results: SA and LA at θ = 45°

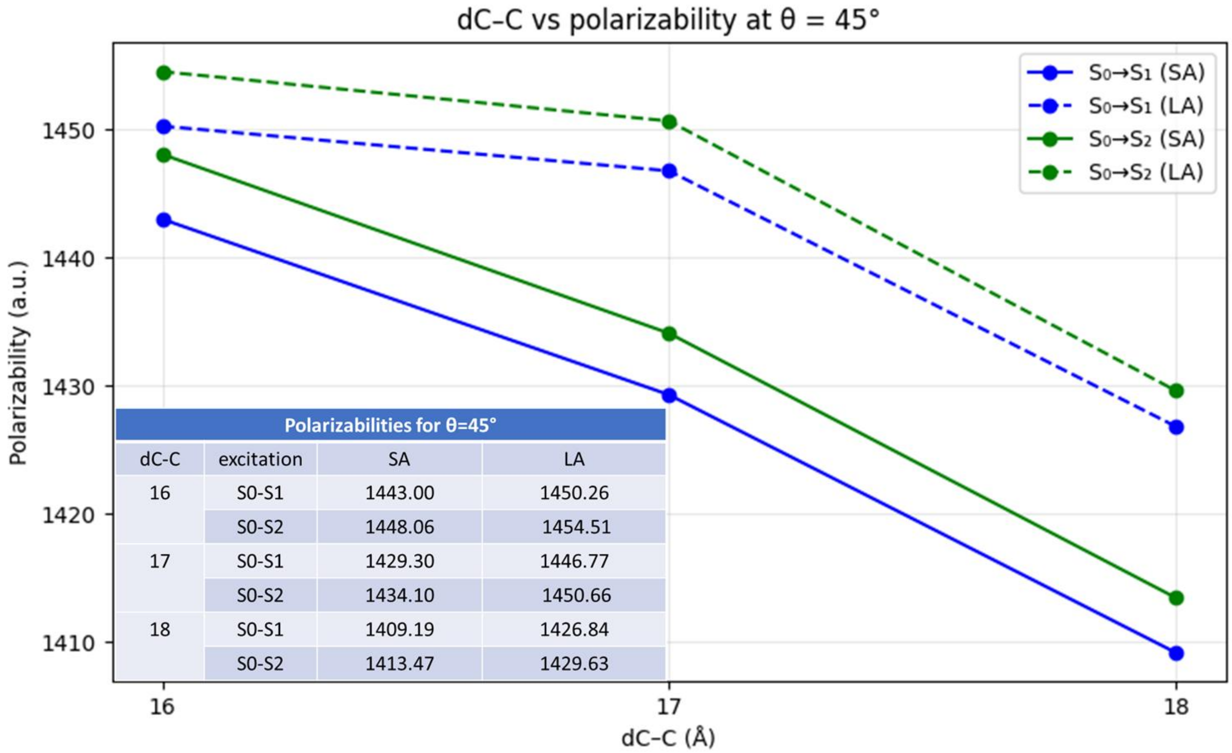

Polarizabilities for θ=45°			
dC-C	excitation	SA	LA
16	S0-S1	1443.00	1450.26
	S0-S2	1448.06	1454.51
17	S0-S1	1429.30	1446.77
	S0-S2	1434.10	1450.66
18	S0-S1	1409.19	1426.84
	S0-S2	1413.47	1429.63

Figure S14 Isotropic polarizability values across the sampled distances for the first and second excitations.

6.2 Stacking plane displacement: Δy

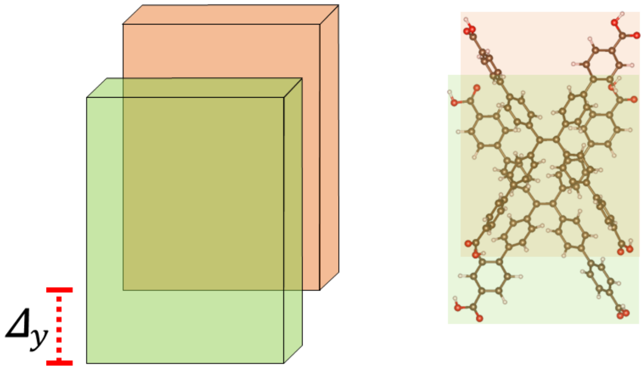

Figure S15. Schematic representation of the plane displacement distance (Δy) between two H_4TCPE linkers.

6.2.1 Displacement up: Δy= +5 Å

Table S11. TD-DFT excitation summary for the H_4TCPE dimer at lateral displacement Δy= +5 Å, comparing stacking distances of 4 and 5 Å for d_{C-C}= 16-18 Å. Listed are 1PA/2PA wavelengths, oscillator strengths, 2PA cross sections (σ_2), and dominant orbital transitions.

Δy = +5 Å (stacking 4 Å)																				
**Exc**		**1PA [nm]**	**2PA [nm]**	**Osc. Str.**	**2PA [GM]**	**Transition**		**1PA [nm]**	**2PA [nm]**	**Osc. Str.**	**2PA [GM]**	**Transition**		**1PA [nm]**	**2PA [nm]**	**Osc. Str.**	**2PA [GM]**	**Transition**
1	**16Å**	340	680	0.369	1.965	HOMO->LUMO (71.9%)	**17Å**	350	700	0.116	2.726	HOMO-1->LUMO (51.2%)	**18Å**	358	717	0.117	2.526	HOMO-1->LUMO (48.1%)
2		323	646	1.513	0.233	HOMO-1->LUMO+1 (67.6%)		338	677	1.259	1.717	HOMO->LUMO+1 (45.6%)		347	694	0.966	1.474	HOMO->LUMO+1 (48.8%)
3		284	568	0.292	14.499	HOMO->LUMO+4 (25.9%)		288	576	0.282	23.256	HOMO->LUMO+2 (35.4%)		291	583	0.266	22.238	HOMO->LUMO+2 (40.2%)
4		279	558	0.819	86.663	HOMO-1->LUMO+2 (43.7%)		283	567	0.109	71.363	HOMO-1->LUMO+2 (17%)		288	576	0.067	43.904	HOMO-1->LUMO+3 (22%)
5		276	553	0.429	5.788	HOMO->LUMO+3 (48.6%)		279	559	0.920	1.938	HOMO-1->LUMO+3 (29%)		282	564	0.405	3.245	HOMO-1->LUMO+3 (18.4%)
6		272	544	1.664	37.995	HOMO-1->LUMO+5 (32.2%)		276	553	0.087	4.029	HOMO->LUMO (49.7%)		280	560	0.122	3.229	HOMO->LUMO (38.5%)
7		269	538	0.049	3.506	HOMO-1->LUMO (70.8%)		274	549	1.365	10.051	HOMO->LUMO+5 (24.2%)		278	557	0.481	3.341	HOMO-1->LUMO+1 (45.6%)
8		266	532	0.005	17.907	HOMO->LUMO+10 (35%)		273	546	0.821	6.925	HOMO-1->LUMO+1 (45.9%)		277	555	2.466	7.981	HOMO->LUMO+5 (22.2%)
9		262	524	0.061	15.162	HOMO-1->LUMO+11 (21.5%)		267	534	0.015	41.074	-		267	535	0.013	27.465	-
10		261	523	0.003	5.430	HOMO->LUMO+1 (59.8%)		265	531	0.027	1.091	-		266	533	0.024	5.331	-
**Δy = +5 Å (stacking 5 Å)**																				
1	**16Å**	332	664	0.360	2.136	HOMO-1->LUMO (68.3%)	**17Å**	347	694	0.500	0.994	HOMO->LUMO (81.5%)	**18Å**	355	710	0.423	0.919	HOMO->LUMO (84%)
2		319	638	1.588	1.573	HOMO->LUMO+1 (59.4%)		322	645	1.068	0.872	HOMO-1->LUMO+1 (72.8%)		326	652	0.798	0.520	HOMO-1->LUMO+1 (75%)
3		282	565	0.127	12.206	HOMO->LUMO+2 (31.6%)		291	582	0.511	25.875	HOMO->LUMO+4 (37%)		291	583	0.373	21.447	HOMO-2->LUMO (27%)
4		279	559	0.850	89.390	HOMO->LUMO+2 (20.8%)		282	564	0.420	25.054	HOMO->LUMO+3 (37.7%)		283	566	0.538	8.307	HOMO->LUMO+3 (35.8%)
5		276	553	0.458	3.128	HOMO-1->LUMO+3 (25.8%)		279	558	1.174	42.758	HOMO-1->LUMO+2 (35.9%)		278	557	0.262	46.638	HOMO-1->LUMO+2 (22.1%)
6		273	547	2.045	53.489	HOMO->LUMO+5 (36.5%)		275	550	1.551	34.385	HOMO-1->LUMO+5 (42.5%)		276	553	2.672	8.630	HOMO->LUMO+5 (26.1%)
7		267	535	0.007	2.821	HOMO->LUMO (67.1%)		268	537	0.004	4.814	HOMO-1->LUMO (69.6%)		270	540	0.002	1.256	HOMO-1->LUMO (84.5%)
8		264	529	0.011	20.821	HOMO-1->LUMO+10 (18.6%)		266	533	0.008	13.339	HOMO->LUMO+8 (19.7%)		266	532	0.008	6.027	HOMO->LUMO+11 (22.9%)
9		260	520	0.008	16.926	HOMO->LUMO+12 (36.7%)		262	524	0.027	40.002	HOMO->LUMO+1 (75%)		263	527	0.057	95.314	HOMO->LUMO+1 (67.5%)
10		258	516	0.002	3.548	HOMO-1->LUMO+1 (74%)		259	519	0.005	48.174	HOMO-1->LUMO+12 (35.7%)		260	520	0.018	166.97	-

6.2.2 Displacement down: Δy= -5 Å

Table S12. TD-DFT excitation summary for the H_4TCPE dimer at lateral displacement Δy= -5 Å, comparing stacking distances of 4 and 5 Å for $d_{C\text{-}C}$= 16-18 Å. Listed are 1PA/2PA wavelengths, oscillator strengths, 2PA cross sections (σ_2), and dominant orbital transition.

Δy = -5 Å (stacking 4 Å)																		
Exc		1PA [nm]	2PA [nm]	Osc. Str.	2PA [GM]	Transition		1PA [nm]	2PA [nm]	Osc. Str.	2PA [GM]	Transition		1PA [nm]	2PA [nm]	Osc. Str.	2PA [GM]	Transition
1	16Å	335	671	0.232	8.395	HOMO-1->LUMO (64.5%) HOMO->LUMO+1 (13.7%)	17Å	346	692	0.176	6.033	HOMO->LUMO (64%) HOMO-1->LUMO+1 (14.7%)	18Å	362	725	0.138	1.087	HOMO->LUMO (58%) HOMO->LUMO+1 (15%)
2		323	646	1.644	1.023	HOMO->LUMO+1 (58.6%) HOMO-1->LUMO (15.3%)		333	667	1.228	1.131	HOMO-1->LUMO+1 (53.7%) HOMO-1->LUMO (16.8%)		350	701	0.955	0.115	HOMO-1->LUMO+1 (55.6%) HOMO-1->LUMO (17.7%)
3		281	563	0.140	18.899	-		286	573	0.336	40.136	HOMO->LUMO+2 (41%)		294	588	0.263	24.829	HOMO->LUMO+2 (41.3%)
4		277	555	0.626	97.070	HOMO-1->LUMO+4 (20.7%)		281	563	0.065	35.709	HOMO-1->LUMO+3 (46.2%)		287	575	0.190	35.372	HOMO-1->LUMO+3 (28.4%)
5		276	552	0.102	4.815	HOMO->LUMO+5 (21.9%)		277	555	0.018	19.952	HOMO->LUMO+5 (20.5%)		283	566	0.304	1.544	HOMO-1->LUMO+4 (28.5%)
6		275	550	0.012	2.679	HOMO->LUMO (75.2%)		277	554	0.271	6.914	HOMO-1->LUMO (65.5%)		278	557	2.379	2.788	HOMO->LUMO+5 (26.3%)
7		272	545	2.440	29.342	HOMO->LUMO+5 (31.7%)		274	549	2.965	3.560	HOMO->LUMO+5 (17.6%)		277	554	0.506	3.339	HOMO-1->LUMO (59.7%)
8		265	531	0.011	22.006	-		268	536	0.024	16.652	HOMO->LUMO+11 (23.4%)		273	546	0.140	5.402	HOMO->LUMO+1 (67.8%)
9		262	524	0.037	11.438	HOMO->LUMO+12 (34.9%)		263	527	0.003	3.325	HOMO->LUMO+1 (71.1%)		266	533	0.025	6.270	HOMO->LUMO+11 (22.4%)
10		257	515	0.009	8.573	HOMO-1->LUMO+1 (81.2%)		262	525	0.023	19.030	HOMO-1->LUMO+12 (21.2%)		265	530	0.011	14.162	HOMO-1->LUMO+12 (18.3%)
Δy = -5 Å (stacking 5 Å)																		
1	16Å	334	668	0.079	10.019	HOMO-1->LUMO (54%) HOMO->LUMO+1 (22.8%)	17Å	339	678	0.127	10.308	HOMO-1->LUMO (59.4%) HOMO->LUMO+1 (17.3%)	18Å	339	679	0.215	9.238	HOMO-1->LUMO (67.1%) HOMO->LUMO+1 (9.8%)
2		324	649	1.874	3.109	HOMO->LUMO+1 (51.7%) HOMO-1->LUMO (24.4%)		328	657	1.444	1.946	HOMO->LUMO+1 (57.3%) HOMO-1->LUMO (18.5%)		328	656	1.064	0.185	HOMO->LUMO+1 (67.7%) HOMO-1->LUMO (9.8%)
3		286	573	0.251	18.104	HOMO->LUMO+4 (26%)		286	572	0.096	24.935	HOMO-1->LUMO+2 (25.4%)		287	575	0.411	38.782	HOMO-1->LUMO+3 (26.9%)
4		281	563	1.047	84.958	HOMO-1->LUMO+2 (25.9%)		283	567	0.137	6.789	HOMO->LUMO (75.3%)		285	570	0.602	22.151	HOMO-1->LUMO+2 (31.5%)
5		280	561	0.051	6.048	HOMO->LUMO (79.4%)		281	563	0.952	65.323	-		278	557	0.144	36.414	HOMO->LUMO+5 (31.3%)
6		277	555	0.109	11.301	HOMO->LUMO+5 (18.6%)		278	557	0.019	17.384	HOMO->LUMO+5 (21.8%)		277	554	0.975	5.600	HOMO->LUMO (53.4%)
7		274	548	2.003	52.660	HOMO->LUMO+5 (23.7%)		275	550	2.548	22.358	-		275	550	1.803	1.485	HOMO->LUMO (28.4%)
8		265	531	0.025	19.959	HOMO-1->LUMO+10 (19.7%)		266	532	0.040	21.712	HOMO-1->LUMO+10 (19.7%)		264	528	0.016	31.923	-
9		263	526	0.009	10.414	HOMO->LUMO+13 (35.6%)		262	525	0.010	13.576	HOMO->LUMO+13 (36.8%)		260	520	0.044	14.164	HOMO->LUMO+12 (28.8%)
10		257	515	0.047	35.961	HOMO-1->LUMO+6 (18.1%)		258	516	0.079	79.726	HOMO-1->LUMO+6 (18.2%)		259	518	0.084	119.54	HOMO-1->LUMO+6 (21.9%)

6.2.3 Displacement up: Δy= +10 Å

Table S13. TD-DFT excitation summary for the H_4TCPE dimer at lateral displacement Δy= +10 Å, comparing stacking distances of 4 and 5 Å for $d_{C\text{-}C}$= 16-18 Å. Listed are 1PA/2PA wavelengths, oscillator strengths, 2PA cross sections (σ_2), and dominant orbital transition.

Δy = +10 Å (stacking 4 Å)																			
Exc		1PA [nm]	2PA [nm]	Osc. Str.	2PA [GM]	Transition		1PA [nm]	2PA [nm]	Osc. Str.	2PA [GM]	Transition		1PA [nm]	2PA [nm]	Osc. Str.	2PA [GM]	Transition
1	16Å	336	672	0.264	4.097	HOMO->LUMO (53.3%)	17Å	342	684	0.069	1.918	HOMO-1->LUMO+1 (36.3%)	18Å	348	696	0.062	2.330	HOMO->LUMO+1 (40.8%)
2		326	653	1.830	0.316	HOMO-1->LUMO+1 (45.9%)		334	668	1.508	0.406	HOMO-1->LUMO (37.1%)		340	680	1.15745	0.205	HOMO-1->LUMO (42.6%)
3		287	574	0.758	56.149	HOMO->LUMO+3 (42.4%)		287	574	0.182	88.297	HOMO->LUMO+3 (25.3%)		289	578	0.16257	62.277	HOMO->LUMO+4 (32.4%)
4		282	565	0.906	107.91	HOMO-1->LUMO+4 (17.7%)		284	569	0.052	61.809	HOMO-1->LUMO+2 (17.3%)		285	570	0.05513	45.173	HOMO-1->LUMO+3 (24.1%)
5		282	564	0.325	16.193	HOMO->LUMO+4 (28.1%)		284	568	1.836	12.809	-		284	569	1.5606	17.664	-
6		280	561	1.654	23.914	HOMO-1->LUMO+5 (37.4%)		281	562	1.853	14.409	HOMO-1->LUMO+3 (26.8%)		281	562	2.30681	6.6877	HOMO-1->LUMO+2 (27.9%)
7		266	532	0.008	13.195	HOMO->LUMO+8 (31.7%)		265	530	0.016	17.318	-		269	539	0.04058	5.438	HOMO->LUMO (54.6%)
8		263	526	0.011	21.215	HOMO-1->LUMO+11 (36%)		264	528	0.002	15.361	HOMO->LUMO+12 (29.1%)		265	531	0.011	30.988	HOMO-1->LUMO+10 (21%)
9		260	521	0.014	7.458	HOMO-1->LUMO (54.6%)		263	527	0.002	23.795	HOMO->LUMO (30.6%)		264	528	0.00143	46.639	HOMO->LUMO+13 (25.7%)
10		257	515	0.031	233.85	HOMO->LUMO+1 (26.4%)		258	517	0.000	111.96	HOMO-1->LUMO+1 (30.2%)		260	520	0.00178	70.226	HOMO-1->LUMO+1 (46.2%)
Δy = +10 Å (stacking 5 Å)																			
1	16Å	327	655	0.024	0.901	HOMO->LUMO+1 (39.6%)	17Å	336	672	0.048	1.309	HOMO-1->LUMO (48.6%)	18Å	346	692	0.410	1.872	HOMO->LUMO (81.8%)
2		321	643	2.026	0.767	HOMO-1->LUMO (38.9%)		329	659	1.603	0.770	HOMO->LUMO+1 (48.9%)		329	658	0.886	0.174	HOMO-1->LUMO+1 (80.3%)
3		283	566	0.785	57.430	HOMO->LUMO+3 (30.8%)		285	571	0.118	38.630	HOMO-1->LUMO+2 (39.7%)		288	576	0.147	55.022	HOMO->LUMO+3 (34%)
4		281	563	0.422	97.638	HOMO-1->LUMO+2 (52.1%)		285	570	0.889	105.92	HOMO->LUMO+3 (33.7%)		284	568	1.729	19.636	HOMO->LUMO+2 (43.7%)
5		280	561	1.746	37.024	HOMO->LUMO+5 (32.2%)		281	563	2.351	28.494	HOMO-1->LUMO+4 (19.8%)		280	561	0.242	49.110	HOMO-1->LUMO+5 (45.2%)
6		278	557	0.787	25.173	HOMO-1->LUMO+4 (20.4%)		281	562	0.684	17.261	HOMO-1->LUMO+5 (36.2%)		279	558	2.147	7.660	HOMO-1->LUMO+4 (55.5%)
7		261	522	0.003	16.829	HOMO-1->LUMO+9 (34.9%)		262	525	0.004	27.969	HOMO-1->LUMO+10 (28.2%)		264	528	0.004	26.32	HOMO->LUMO+10 (40.7%)
8		261	522	0.003	26.688	HOMO->LUMO+11 (37.6%)		261	523	0.004	28.044	HOMO->LUMO+11 (30.6%)		259	518	0.001	31.865	HOMO-1->LUMO+11 (32.9%)
9		254	509	0.024	396.91	HOMO->LUMO+7 (34.2%)		256	512	0.004	703.13	-		257	515	0.013	3.863	HOMO-1->LUMO (64.8%)
10		253	507	0.051	232.59	HOMO-1->LUMO+6 (29.5%)		255	511	0.105	2.959	-		257	514	0.051	284.77	-

6.2.4 Displacement down: Δy= -10 Å

Table S14. TD-DFT excitation summary for the H_4TCPE dimer at lateral displacement Δy= -10 Å, comparing stacking distances of 4 and 5 Å for $d_{C\text{-}C}$= 16-18 Å. Listed are 1PA/2PA wavelengths, oscillator strengths, 2PA cross sections (σ_2), and dominant orbital transition.

Exc		1PA [nm]	2PA [nm]	Osc. Str.	2PA [GM]	Transition		1PA [nm]	2PA [nm]	Osc. Str.	2PA [GM]	Transition		1PA [nm]	2PA [nm]	Osc. Str.	2PA [GM]	Transition
**Δy = -10 Å (stacking 4 Å)**																		
1	16Å	332	664	0.083	0.705	HOMO-1->LUMO (44.4%)	17Å	342	684	0.060	1.365	HOMO->LUMO+1 (36.6%)	18Å	342	684	0.108	2.146	HOMO->LUMO+1 (59.7%)
2		324	648	1.946	0.384	HOMO->LUMO+1 (32.2%)		334	668	1.498	0.169	HOMO-1->LUMO (37.9%)		334	669	1.103	0.390	HOMO-1->LUMO+2 (27.2%)
3		283	567	0.466	184.242	HOMO->LUMO+5 (22.8%)		287	574	0.157	94.082	HOMO->LUMO+4 (18.3%)		287	575	0.243	86.584	HOMO->LUMO+3 (30.8%)
4		282	565	0.074	7.091	HOMO-1->LUMO+2 (20.8%)		284	569	0.012	64.913	HOMO-1->LUMO+3 (20.6%)		285	570	0.341	41.691	HOMO->LUMO+3 (23.9%)
5		282	564	2.184	14.460	HOMO->LUMO+3 (46.7%)		283	567	1.709	9.230	HOMO->LUMO+2 (19.4%)		282	565	1.597	13.190	HOMO-1->LUMO+2 (25.3%)
6		280	560	0.939	20.730	HOMO-1->LUMO+4 (48.5%)		281	563	2.063	6.353	HOMO-1->LUMO+2 (25.2%)		280	561	1.943	5.841	HOMO->LUMO+5 (30.1%)
7		263	526	0.005	33.665	-		264	529	0.017	23.921	HOMO-1->LUMO+10 (21.5%)		266	533	0.069	4.747	HOMO->LUMO (59.1%)
8		262	525	0.006	8.565	-		264	528	0.003	11.851	HOMO->LUMO+6 (24.9%)		262	525	0.014	65.565	HOMO-1->LUMO+12 (28.7%)
9		257	514	0.094	37.160	-		263	526	0.001	23.866	HOMO->LUMO (38%)		261	522	0.007	33.796	HOMO->LUMO+13 (37.1%)
10		256	512	0.005	635.18	-		258	516	0.001	135.17	HOMO-1->LUMO+1 (28%)		258	516	0.075	369.37	HOMO-1->LUMO+6 (24.2%)
**Δy = -10 Å (stacking 5 Å)**																		
1	16Å	324	649	0.011	0.777	HOMO-1->LUMO (38.2%)	17Å	332	664	0.179	0.678	HOMO-1->LUMO (60.6%)	18Å	339	678	0.229	1.111	HOMO-1->LUMO (69.3%)
2		318	637	2.055	0.519	HOMO->LUMO+1 (38.7%)		324	649	1.462	0.751	HOMO->LUMO+1 (61.2%)		330	660	1.057	0.383	HOMO->LUMO+1 (70.1%)
3		282	564	1.047	111.01	HOMO->LUMO+4 (31.3%)		283	567	0.182	88.315	HOMO-1->LUMO+3 (40.5%)		286	572	0.017	75.498	HOMO-1->LUMO+3 (50.7%)
4		282	564	0.277	38.327	HOMO-1->LUMO+2 (25.4%)		282	565	0.470	71.916	HOMO->LUMO+4 (39.5%)		283	567	0.009	51.036	HOMO->LUMO+4 (36.4%)
5		279	559	2.313	76.065	HOMO-1->LUMO+3 (24.9%)		281	562	2.432	12.477	HOMO-1->LUMO+2 (42.7%)		283	566	1.936	15.272	HOMO-1->LUMO+2 (44.8%)
6		278	557	0.093	1.665	HOMO->LUMO+5 (36.5%)		278	557	0.941	20.925	HOMO->LUMO+5 (46%)		281	563	2.265	9.303	HOMO->LUMO+5 (32.7%)
7		260	521	0.001	22.162	HOMO-1->LUMO+6 (36.4%)		261	523	0.001	31.316	HOMO-1->LUMO+11 (27.2%)		262	525	0.024	4.351	HOMO->LUMO (54%)
8		260	520	0.013	22.219	HOMO->LUMO+7 (28.7%)		260	520	0.014	27.301	HOMO->LUMO+13 (36.6%)		261	523	0.003	54.353	HOMO-1->LUMO+12 (24.3%)
9		254	509	0.051	304.01	HOMO-1->LUMO+6 (36.3%)		256	513	0.011	138.52	HOMO->LUMO (45.7%)		260	520	0.000	33.698	HOMO->LUMO+13 (35%)
10		254	509	0.018	381.92	HOMO->LUMO+7 (34.4%)		255	511	0.070	455.56	HOMO-1->LUMO+6 (35.9%)		257	515	0.078	538.66	HOMO-1->LUMO+6 (33.5%)

6.2.5 EDD and main orbital transitions: comparison of 4 Å vs 5 Å at Δy= -5 Å

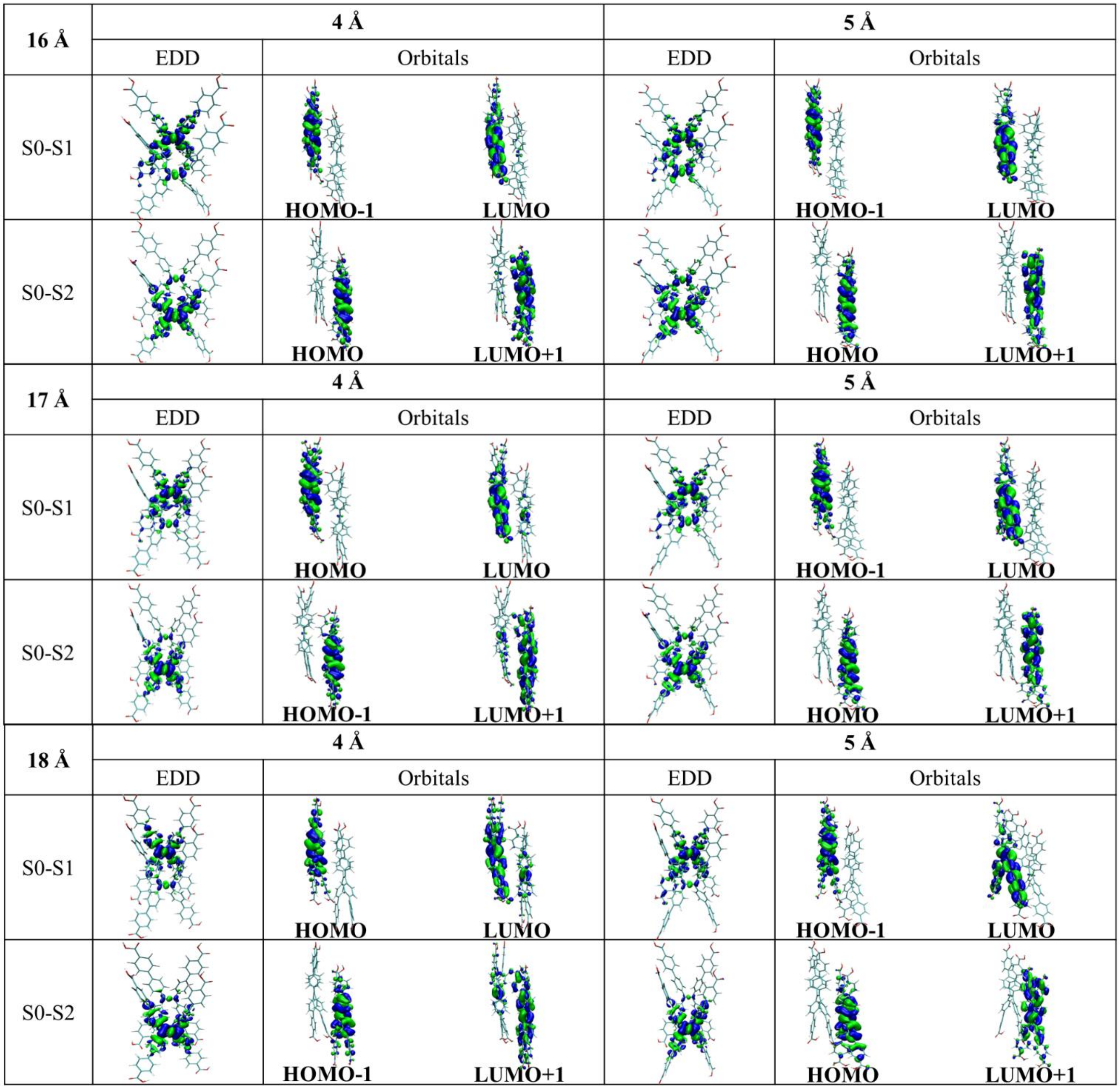

Figure S16. Comparison of stacking distances (4 Å and 5 Å) for d_{C-C}= 16-18 Å at Δy= -5 Å displacement. EDD plots and dominant orbital transitions for the first and second excitations.

6.2.6 Polarizability results: plane displacement Δy = 0 vs -5 Å displacement.

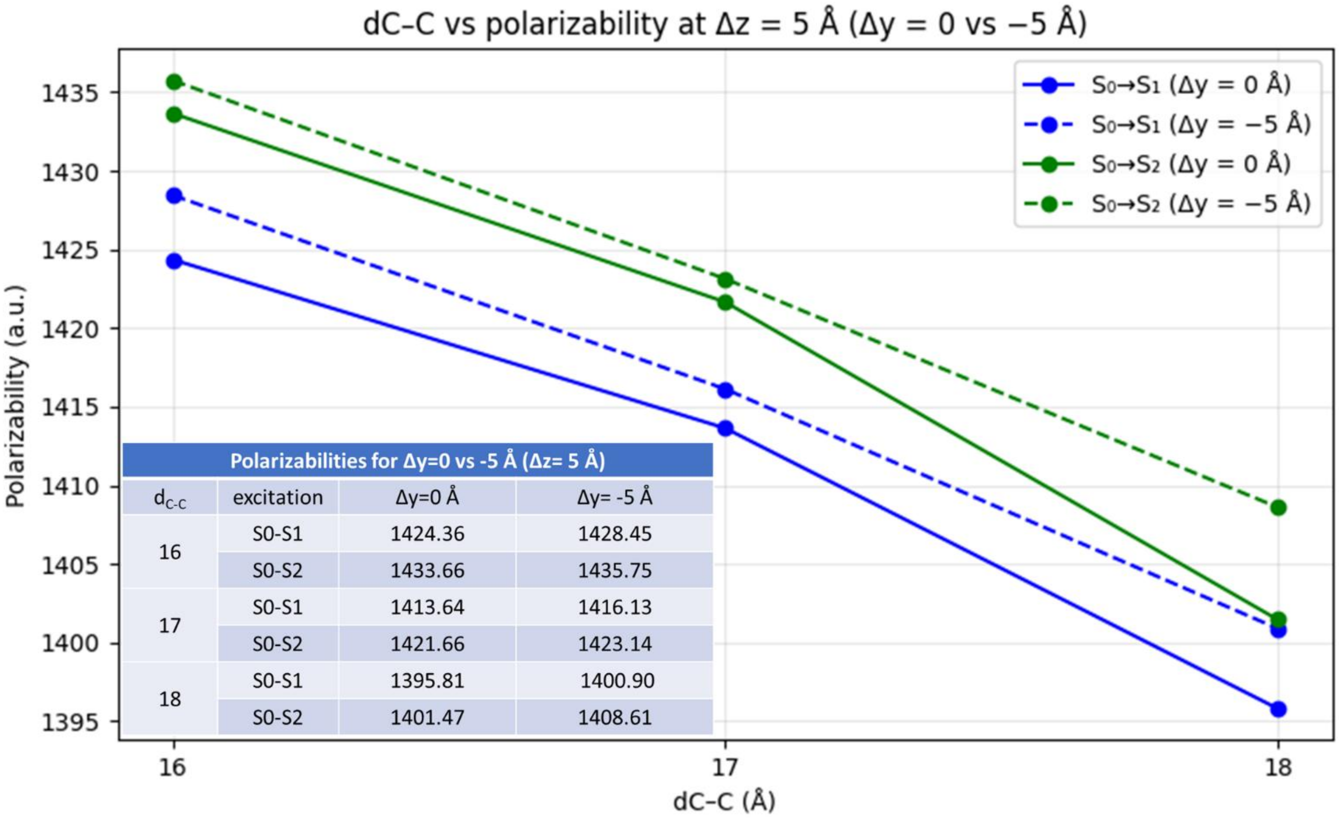

Polarizabilities for Δy=0 vs -5 Å (Δz= 5 Å)

d_{C-C}	excitation	Δy=0 Å	Δy= -5 Å
16	S0-S1	1424.36	1428.45
	S0-S2	1433.66	1435.75
17	S0-S1	1413.64	1416.13
	S0-S2	1421.66	1423.14
18	S0-S1	1395.81	1400.90
	S0-S2	1401.47	1408.61

Figure S17. Isotropic polarizability values across the sampled distances for the first (blue) and second (green) excitations. Solid line is representing 0 Å displacement (stacking) and dashed line represents -5 Å displacements.

6.3 Stacking plane displacement: Δx

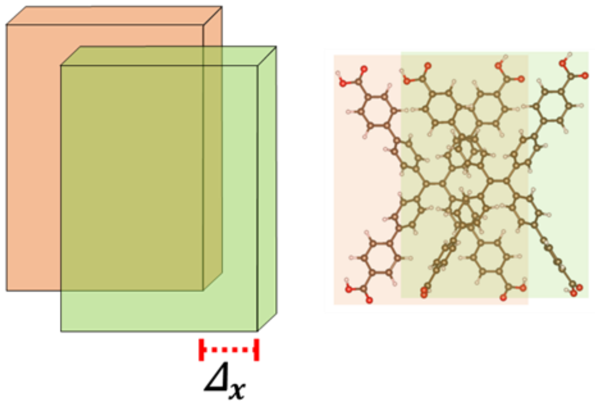

Figure S18. Schematic representation of the plane displacement distance (Δx) between two H_4TCPE linkers.

6.3.1 Displacement right: Δx= +5 Å

Table S15. TD-DFT excitation summary for the H_4TCPE dimer at lateral displacement Δx= +5 Å, comparing stacking distances of 4 and 5 Å for $d_{C\text{-}C}$= 16-18 Å. Listed are 1PA/2PA wavelengths, oscillator strengths, 2PA cross sections (σ_2), and dominant orbital transition.

Δx = +5 Å (stacking 4 Å)																		
**Exc**		**1PA [nm]**	**2PA [nm]**	**Osc. Str.**	**2PA [GM]**	**Transition**		**1PA [nm]**	**2PA [nm]**	**Osc. Str.**	**2PA [GM]**	**Transition**		**1PA [nm]**	**2PA [nm]**	**Osc. Str.**	**2PA [GM]**	**Transition**
1		341	683	0.459	0.885	HOMO->LUMO (65.1%)		344	689	0.015	1.057	HOMO-1->LUMO (49%)		351	702	0.041	0.102	HOMO-1->LUMO (52.4%)
2		332	665	1.439	1.115	HOMO-3->LUMO+1 (67.8%)		341	683	1.507	0.592	HOMO->LUMO+1 (28.1%)		349	698	1.148	0.770	HOMO->LUMO (28.9%)
3		288	577	0.089	32.053	HOMO-1->LUMO+3 (39.3%)		290	580	0.007	42.731	HOMO->LUMO+2 (24.8%)		290	581	0.087	0.349	HOMO-1->LUMO+3 (20.6%)
4		286	573	0.025	14.582	HOMO->LUMO+2 (28.3%)		289	578	0.000	0.404	-		289	579	0.024	38.187	HOMO->LUMO+2 (27.7%)
5	**16Å**	283	566	0.175	118.93	HOMO->LUMO+5 (35.1%)	**17Å**	286	572	0.030	77.644	HOMO->LUMO+5 (25.7%)	**18Å**	288	576	0.019	33.957	HOMO->LUMO+5 (21.4%)
6		280	561	2.842	14.488	HOMO-1->LUMO+4 (28.7%)		282	564	3.430	9.497	HOMO->LUMO+3 (19.1%)		282	564	3.460	11.476	HOMO-1->LUMO+2 (23.4%)
7		268	537	0.011	1.100	HOMO-1->LUMO (43.7%)		270	541	0.017	2.057	HOMO->LUMO (64.5%)		272	545	0.023	2.493	HOMO->LUMO (58.5%)
8		268	536	0.013	1.120	HOMO->LUMO+1 (47%)		268	536	0.007	1.927	HOMO-1->LUMO+1 (62.1%)		270	541	0.021	3.664	HOMO-1->LUMO+1 (53.7%)
9		266	533	0.005	24.010	HOMO->LUMO+8 (27.3%)		265	531	0.005	32.773	HOMO-1->LUMO+8 (18.1%)		265	530	0.003	33.278	-
10		264	529	0.003	12.620	HOMO-1->LUMO+8 (18.3%)		265	530	0.003	10.447	-		264	529	0.002	13.479	-
**Δx = +5 Å (stacking 5 Å)**																		
1		335	671	0.022	0.422	HOMO->LUMO+1 (38.5%)		345	691	0.033	0.205	HOMO->LUMO+1 (45.2%)		351	703	0.054	0.084	HOMO->LUMO+1 (48.7%)
2		331	663	1.896	1.210	HOMO-1->LUMO+1 (31.9%)		343	686	1.517	1.200	HOMO-1->LUMO+1 (30.7%)		350	700	1.181	1.156	HOMO-1->LUMO+1 (31.8%)
3		286	573	0.009	38.444	HOMO-1->LUMO+2 (21.6%)		290	580	0.016	47.883	HOMO-1->LUMO+2 (20.4%)		291	582	0.019	38.177	HOMO->LUMO+2 (19%)
4		285	570	0.175	0.881	HOMO->LUMO+5 (23.5%)		288	576	0.105	0.946	HOMO->LUMO+5 (25.4%)		289	579	0.009	3.837	HOMO->LUMO+5 (27.2%)
5	**16Å**	281	563	0.166	137.52	HOMO-1->LUMO+4 (19.7%)	**17Å**	284	569	0.042	91.948	HOMO->LUMO+4 (18.2%)	**18Å**	286	572	0.019	48.609	HOMO->LUMO+4 (27.1%)
6		280	560	2.876	16.434	HOMO->LUMO+3 (32%)		281	563	3.345	13.673	HOMO->LUMO+3 (26.7%)		282	565	3.628	12.841	HOMO->LUMO+3 (23.9%)
7		265	531	0.008	7.177	HOMO->LUMO (17.5%)		270	541	0.018	3.273	HOMO->LUMO (46.1%)		272	545	0.023	3.886	HOMO->LUMO (49.2%)
8		264	529	0.005	27.878	-		268	537	0.009	2.119	HOMO-1->LUMO+1 (52.2%)		271	542	0.013	3.586	HOMO-1->LUMO+1 (48%)
9		263	526	0.002	6.148	HOMO->LUMO (31%)		265	531	0.011	41.634	HOMO-1->LUMO+8 (23.5%)		265	531	0.007	41.721	HOMO->LUMO+9 (21.1%)
10		262	525	0.011	7.475	HOMO-1->LUMO (30.6%)		265	530	0.001	9.878	HOMO-1->LUMO+8 (19.1%)		264	529	0.000	12.962	HOMO-1->LUMO+9 (24.2%)

6.3.2 Displacement left: Δx= -5 Å

Table S16. TD-DFT excitation summary for the H_4TCPE dimer at lateral displacement Δx= -5 Å, comparing stacking distances of 4 and 5 Å for $d_{C\text{-}C}$= 16-18 Å. Listed are 1PA/2PA wavelengths, oscillator strengths, 2PA cross sections (σ_2), and dominant orbital transition.

Δx = -5 Å (stacking 4 Å)

Exc		1PA [nm]	2PA [nm]	Osc. Str.	2PA [GM]	Transition		1PA [nm]	2PA [nm]	Osc. Str.	2PA [GM]	Transition		1PA [nm]	2PA [nm]	Osc. Str.	2PA [GM]	Transition
1	16Å	343	686	0.508	1.419	HOMO-1->LUMO (76.1%)	17Å	352	704	0.474	0.699	HOMO-1->LUMO (46.8%)	18Å	357	715	0.432	0.129	HOMO-1->LUMO (52.6%)
2		333	667	1.346	1.476	HOMO->LUMO+1 (72.4%)		343	687	1.035	1.546	HOMO->LUMO+1 (40.2%)		350	700	0.754	0.639	HOMO->LUMO+1 (47.9%)
3		288	577	0.061	36.964	HOMO-1->LUMO+2 (45.4%)		291	583	0.021	66.894	HOMO->LUMO+2 (35.1%)		292	584	0.043	30.722	HOMO-3->LUMO (25.6%)
4		286	572	0.007	13.470	HOMO->LUMO+3 (34.5%)		290	581	0.039	9.950	HOMO-1->LUMO+2 (19.6%)		291	582	0.049	17.601	HOMO->LUMO+2 (23.9%)
5		283	566	0.255	100.79	HOMO->LUMO+5 (37.8%)		285	571	0.388	53.924	HOMO-1->LUMO+4 (18.7%)		288	576	0.113	27.655	HOMO->LUMO+5 (18.8%)
6		280	560	2.798	20.212	HOMO-1->LUMO+4 (24.7%)		281	563	3.009	7.418	HOMO->LUMO+5 (19.8%)		282	564	3.425	4.532	-
7		271	542	0.015	0.562	HOMO->LUMO (79.1%)		274	549	0.007	1.423	HOMO->LUMO (47.5%)		276	552	0.021	2.773	HOMO->LUMO (53.6%)
8		268	536	0.005	15.404	HOMO-1->LUMO+8 (35.8%)		271	542	0.016	4.091	HOMO-1->LUMO+1 (48.5%)		272	544	0.011	5.422	HOMO-1->LUMO+1 (54.6%)
9		265	530	0.004	11.322	HOMO-1->LUMO+1 (36.9%)		268	536	0.006	31.055	-		267	535	0.004	26.248	-
10		264	529	0.017	9.874	HOMO-1->LUMO+1 (44.3%)		266	532	0.005	18.406	-		265	530	0.005	18.266	HOMO->LUMO+12 (17.8%)

Δx = -5 Å (stacking 5 Å)

Exc		1PA [nm]	2PA [nm]	Osc. Str.	2PA [GM]	Transition		1PA [nm]	2PA [nm]	Osc. Str.	2PA [GM]	Transition		1PA [nm]	2PA [nm]	Osc. Str.	2PA [GM]	Transition
1	16Å	336	672	0.158	3.150	HOMO->LUMO+1 (51.5%)	17Å	346	692	0.065	1.366	HOMO-1->LUMO (43.2%)	18Å	351	702	0.013	0.642	HOMO-1->LUMO (43.5%)
2		331	663	1.827	0.619	HOMO-1->LUMO (37.8%)		343	686	1.517	0.538	HOMO->LUMO (44.2%)		349	698	1.232	0.225	HOMO->LUMO (45.9%)
3		289	578	0.043	68.976	HOMO-1->LUMO+2 (22.5%)		291	583	0.039	61.244	HOMO->LUMO+2 (35.9%)		292	584	0.036	37.452	HOMO->LUMO+2 (33.7%)
4		287	574	0.439	3.770	HOMO-1->LUMO+2 (23.9%)		289	579	0.187	2.360	HOMO-1->LUMO+2 (19.6%)		289	579	0.004	1.789	-
5		282	564	1.441	65.150	HOMO-1->LUMO+4 (49.6%)		284	569	0.514	67.215	HOMO->LUMO+4 (26.1%)		286	572	0.061	44.605	HOMO->LUMO+4 (26.6%)
6		276	553	1.229	42.396	HOMO->LUMO+5 (50.5%)		281	563	2.792	15.840	HOMO->LUMO+5 (19.3%)		282	565	3.622	5.210	HOMO->LUMO+3 (24.6%)
7		267	534	0.020	1.733	HOMO->LUMO (58.4%)		271	542	0.019	2.749	HOMO-1->LUMO (27.1%)		272	544	0.010	4.140	HOMO-1->LUMO+1 (27.3%)
8		265	531	0.001	3.735	HOMO-1->LUMO+1 (22.8%)		269	539	0.004	2.281	HOMO->LUMO (28.8%)		271	542	0.005	5.678	HOMO->LUMO+1 (29%)
9		264	529	0.010	35.223	HOMO->LUMO+8 (22.2%)		266	532	0.005	38.667	-		265	531	0.001	34.295	-
10		262	525	0.001	6.928	HOMO-1->LUMO+1 (42.2%)		265	531	0.002	16.966	-		264	529	0.006	16.203	HOMO->LUMO+12 (17.4%)

6.3.3 Displacement right: Δx= +10 Å

Table S17. TD-DFT excitation summary for the H_4TCPE dimer at lateral displacement Δx= +10 Å, comparing stacking distances of 4 and 5 Å for d_{C-C}= 16-18 Å. Listed are 1PA/2PA wavelengths, oscillator strengths, 2PA cross sections (σ_2), and dominant orbital transition.

Δx = +10 Å (stacking 4 Å)																			
Exc		1PA [nm]	2PA [nm]	Osc. Str.	2PA [GM]	Transition		1PA [nm]	2PA [nm]	Osc. Str.	2PA [GM]	Transition		1PA [nm]	2PA [nm]	Osc. Str.	2PA [GM]	Transition
1		328	656	1.798	0.364	HOMO->LUMO (57.2%)		335	670	1.359	0.486	HOMO->LUMO (80.9%)		341	682	1.520	0.714	HOMO->LUMO (32%)
2		323	646	0.531	0.0719	HOMO-1->LUMO+1 (48.9%)		329	659	0.530	1.083	HOMO-1->LUMO+1 (80.2%)		338	676	0.000	0.167	HOMO-1->LUMO (41.5%)
3		285	571	0.074	63.192	HOMO->LUMO+2 (33%)		287	575	0.143	59.151	HOMO->LUMO+2 (46.3%)		290	581	0.039	52.194	HOMO->LUMO+2 (35%)
4		281	563	0.438	82.170	HOMO->LUMO+3 (55.4%)		282	565	0.625	51.088	HOMO-1->LUMO+3 (46.1%)		285	570	0.408	13.247	HOMO-1->LUMO+3 (35.5%)
5	16Å	280	560	0.005	48.490	HOMO-1->LUMO+4 (23.3%)	17Å	282	564	0.281	48.810	HOMO->LUMO+4 (51.9%)	18Å	283	567	0.087	36.243	HOMO->LUMO+4 (33.5%)
6		278	556	2.733	20.642	-		277	555	2.642	19.109	HOMO-1->LUMO+5 (32.4%)		278	557	3.261	9.161	HOMO-1->LUMO+2 (24%)
7		262	525	0.004	13.354	HOMO->LUMO+10 (36.3%)		263	526	0.004	23.264	HOMO->LUMO+11 (27.4%)		262	525	0.022	7.856	-
8		261	523	0.000	9.881	HOMO->LUMO+6 -		261	522	0.003	23.897	HOMO-1->LUMO+10 (40.9%)		260	521	0.001	1.595	-
9		260	520	0.015	34.152	HOMO-1->LUMO+11 (24%)		256	513	0.044	141.07	HOMO->LUMO+6 (34.7%)		260	521	0.015	56.002	-
10		258	517	0.003	293.75	-		255	510	0.007	15.363	-		254	509	0.008	83.410	-
Δx = +10 Å (stacking 5 Å)																			
1		327	655	2.23	1.238	HOMO->LUMO+1 (59.1%)		332	664	1.208	1.929	HOMO-1->LUMO (74.6%)		348	696	0.971	0.748	HOMO->LUMO (85.2%)
2		325	650	0.134	1.0595	HOMO-1->LUMO (50.2%)		325	651	0.713	2.455	HOMO->LUMO+1 (75.7%)		338	677	0.554	0.844	HOMO-1->LUMO+1 (82.5%)
3		287	574	0.012	87.627	HOMO->LUMO+3 (29.5%)		285	570	0.124	69.888	HOMO-1->LUMO+2 (46.2%)		294	588	0.245	55.002	HOMO->LUMO+2 (45.2%)
4		282	564	0.335	7.772	HOMO-1->LUMO+2 (23.7%)		281	562	0.553	28.861	HOMO-1->LUMO+4 (51.3%)		286	572	0.006	10.832	HOMO-1->LUMO+3 (23%)
5	16Å	280	560	0.149	110.36	HOMO-1->LUMO+4 (37.3%)	17Å	279	559	1.165	53.406	HOMO->LUMO+5 (31.7%)	18Å	284	568	0.396	50.865	HOMO->LUMO+5 (36.7%)
6		279	558	2.874	10.294	HOMO->LUMO+5 (30.1%)		276	552	1.764	54.976	HOMO->LUMO+3 (29.2%)		281	563	3.287	12.263	HOMO-1->LUMO+4 (28.3%)
7		261	523	0.007	26.680	HOMO->LUMO+11 (39.3%)		261	522	0.007	32.614	HOMO-1->LUMO+12 (25.1%)		264	529	0.003	19.057	HOMO->LUMO+11 (26%)
8		261	522	0.006	22.983	HOMO-1->LUMO+12 (35.8%)		260	520	0.008	25.148	HOMO->LUMO+11 (34.8%)		260	521	0.009	42.396	HOMO-1->LUMO+12 (29.5%)
9		255	511	0.013	9.3049	-		254	509	0.023	224.43	HOMO-1->LUMO+6 (38.6%)		259	519	0.122	119.77	HOMO->LUMO+6 (25.5%)
10		254	509	0.064	492.56	HOMO-1->LUMO+7 (19.3%)		253	506	0.005	1.254	-		257	515	0.002	7.599	-

6.3.4 Displacement left: Δx= -10 Å

Table S18. TD-DFT excitation summary for the H_4TCPE dimer at lateral displacement Δx= -10 Å, comparing stacking distances of 4 and 5 Å for $d_{C\text{-}C}$= 16-18 Å. Listed are 1PA/2PA wavelengths, oscillator strengths, 2PA cross sections (σ_2), and dominant orbital transition.

Δx = -10 Å (stacking 4 Å)																		
Exc		1PA [nm]	2PA [nm]	Osc. Str.	2PA [GM]	Transition		1PA [nm]	2PA [nm]	Osc. Str.	2PA [GM]	Transition		1PA [nm]	2PA [nm]	Osc. Str.	2PA [GM]	Transition
1		327	655	1.678	0.186	HOMO-1->LUMO (76.8%)		335	670	1.593	0.929	HOMO-1->LUMO (52.4%)		384	768	0.031	1.821	HOMO->LUMO (42.7%)
2		321	643	0.658	0.687	HOMO->LUMO+1 (75.3%)		330	661	0.277	1.380	HOMO->LUMO+1 (61.2%)		340	681	0.978	7.560	HOMO-1->LUMO (36.8%)
3		284	659	0.068	58.554	HOMO->LUMO+2 (33.6%)		284	569	0.052	64.893	HOMO->LUMO+2 (27.6%)		339	678	0.433	1.808	HOMO->LUMO+1 (51.3%)
4		280	560	0.498	67.982	HOMO-1->LUMO+3 (53.3%)		281	563	0.555	38.095	HOMO-1->LUMO+3 (50.4%)		333	666	0.180	2.337	HOMO-2->LUMO+1 (27.3%)
5	16Å	279	558	0.105	53.614	HOMO->LUMO+5 (50.9%)	17Å	280	560	0.305	30.320	HOMO->LUMO+5 (47.3%)	18Å	297	594	0.263	97.998	HOMO-1->LUMO+2 (26.1%)
6		277	554	2.678	9.792	-		275	551	2.677	5.118	HOMO-1->LUMO+2 (23%)		286	573	0.001	8.957	HOMO-38->LUMO (42.5%)
7		262	524	0.005	21.750	HOMO-1->LUMO+10 (45.8%)		262	525	0.007	22.274	HOMO-1->LUMO+11 (20.6%)		284	569	0.208	9.372	HOMO->LUMO+5 (20.7%)
8		260	520	0.011	18.960	HOMO->LUMO+12 (35.2%)		261	523	0.012	16.618	HOMO-1->LUMO+12 (30.6%)		282	565	0.312	37.269	HOMO-1->LUMO+4 (41.4%)
9		256	512	0.002	159.13	-		258	516	0.018	12.046	-		278	557	2.847	17.048	-
10		254	509	0.004	136.62	-		254	508	0.007	84.073	-		270	540	0.043	14.474	HOMO->LUMO (30.8%)
**Δx = -10 Å (stacking 5 Å)**																		
1		326	652	1.872	0.136	HOMO->LUMO (70.5%)		337	675	1.586	1.991	HOMO->LUMO+1 (71%)		342	684	1.553	0.889	HOMO-1->LUMO (43%)
2		321	643	0.499	0.367	HOMO-1->LUMO+1 (64.9%)		333	666	0.308	2.629	HOMO-1->LUMO (62.6%)		338	677	0.003	0.115	HOMO->LUMO+1 (47.2%)
3		285	570	0.034	59.036	HOMO-1->LUMO+2 (25.3%)		289	578	0.154	73.670	HOMO-1->LUMO+2 (43.8%)		290	581	0.025	57.073	HOMO-1->LUMO+2 (21.3%)
4		280	560	0.163	2.770	HOMO-1->LUMO+3 (28.6%)		284	569	0.510	60.596	HOMO->LUMO+3 (51.6%)		285	570	0.158	0.681	HOMO->LUMO+4 (22%)
5		279	559	0.051	126.02	HOMO->LUMO+5 (28.2%)		281	563	0.065	27.752	HOMO-1->LUMO+4 (42.1%)		284	568	0.051	47.821	HOMO-1->LUMO+3 (33.8%)
6	16Å	278	556	3.135	6.249	HOMO->LUMO+4 (26.9%)	17Å	279	558	2.873	6.669	HOMO->LUMO+5 (27.3%)	18Å	280	560	3.675	7.349	HOMO->LUMO+5 (22.9%)
7		261	523	0.007	22.592	HOMO->LUMO+11 (35.9%)		268	536	0.035	9.316	HOMO->LUMO (27%)		262	525	0.013	27.677	HOMO->LUMO (32.9%)
8		260	520	0.007	20.663	HOMO-1->LUMO+12 (32.6%)		262	525	0.006	19.205	HOMO->LUMO+10 (43.5%)		261	522	0.009	67.010	HOMO-1->LUMO+10 (26.9%)
9		255	510	0.003	137.95	HOMO-1->LUMO+6 (20.2%)		261	523	0.020	37.986	HOMO-1->LUMO+11 (43.3%)		260	521	0.005	12.631	HOMO->LUMO+11 (25.8%)
10		254	508	0.002	221.99	-		259	518	0.008	8.229	-		256	513	0.027	380.04	HOMO-1->LUMO+6 (31.8%)

6.4 Plane rotation

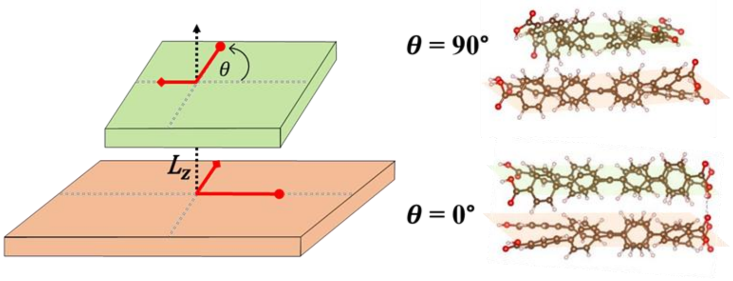

Figure S19. Schematic representation of the plane rotation angle between two H_4TCPE linkers.

6.4.1 Rotation angle: θ= 0° corresponds also to Δy=0

Table S19. TD-DFT excitation summary for the H_4TCPE dimer at ϑ= 0°, comparing stacking distances of 4 and 5 Å for d_{C-C}= 16-18 Å. Listed are 1PA/2PA wavelengths, oscillator strengths, 2PA cross sections (σ_2), and dominant orbital transition.

θ = 0°(stacking 4 Å)																			
Exc		1PA [nm]	2PA [nm]	Osc. Str.	2PA [GM]	Transition		1PA [nm]	2PA [nm]	Osc. Str.	2PA [GM]	Transition		1PA [nm]	2PA [nm]	Osc. Str.	2PA [GM]	Transition
1	16Å	350	700	0.163	3.958	HOMO->LUMO (38.1%) HOMO->LUMO+1 (20.4%)	17Å	353	706	0.102	5.916	HOMO->LUMO (49.7%) HOMO-1->LUMO+1 (25.2%)	18Å	364	728	0.353	4.583	HOMO->LUMO (49.5%) HOMO-1->LUMO (29.4%)
2	16Å	336	673	1.680	2.590	HOMO-1->LUMO (48.1%) HOMO->LUMO+1 (32.2%)	17Å	340	681	1.351	2.475	HOMO-1->LUMO (40.1%) HOMO->LUMO+1 (37.3%)	18Å	338	676	0.817	0.191	HOMO->LUMO+1 (40%) HOMO-1->LUMO+1 (35.7%)
3	16Å	295	591	0.222	34.272	HOMO->LUMO+3 (39.2%)	17Å	297	594	0.234	54.322	HOMO->LUMO+2 (31.2%)	18Å	302	605	0.198	54.256	HOMO->LUMO+2 (29.2%)
4	16Å	291	582	0.054	86.758	HOMO-1->LUMO+2 (26.7%)	17Å	292	585	0.049	80.082	HOMO->LUMO+3 (29.9%)	18Å	298	596	0.692	2.744	HOMO->LUMO+3 (29.4%)
5	16Å	285	570	0.009	97.689	-	17Å	285	570	0.012	59.695	-	18Å	292	585	0.015	4.999	HOMO-1->LUMO (50.4%)
6	16Å	283	567	0.131	17.078	HOMO->LUMO (28.7%)	17Å	284	568	0.060	1.605	HOMO-1->LUMO (27.1%)	18Å	285	570	0.068	39.228	-
7	16Å	280	560	2.622	12.013	-	17Å	280	561	0.566	1.138	HOMO-1->LUMO+1 (40.3%)	18Å	281	562	2.520	6.718	-
8	16Å	277	554	0.010	0.152	HOMO-1->LUMO+1 (64.7%)	17Å	279	558	2.377	10.487	-	18Å	272	545	0.024	32.024	-
9	16Å	271	542	0.012	22.455	HOMO->LUMO+8 (38%)	17Å	270	541	0.011	38.818	HOMO->LUMO+8 (30.1%)	18Å	272	544	0.002	0.868	HOMO-1->LUMO+1 (44.5%)
10	16Å	268	537	0.014	5.527	HOMO-1->LUMO+8 (33.7%)	17Å	268	537	0.036	8.241	HOMO-1->LUMO+8 (32.2%)	18Å	268	537	0.103	111.99	-
**θ = 0°(stacking 5 Å)**																			
1	16Å	341	683	0.249	9.737	HOMO->LUMO (49.5%) HOMO->LUMO+1 (14.0%)	17Å	347	694	0.211	8.045	HOMO->LUMO (47.3%) HOMO->LUMO+1 (17.2%)	18Å	342	685	0.075	7.533	HOMO->LUMO (37.2%) HOMO->LUMO+1 (18.2%)
2	16Å	328	657	1.621	2.743	HOMO-1->LUMO (42.9%) HOMO->LUMO+1 (17.8%)	17Å	335	670	1.324	2.598	HOMO-1->LUMO (46.3%) HOMO->LUMO+1 (18.5%)	18Å	333	667	1.136	1.503	HOMO-1->LUMO (46.3%) HOMO-1->LUMO+1 (24.1%)
3	16Å	292	584	0.307	43.712	HOMO->LUMO+3 (44.7%)	17Å	292	585	0.247	39.737	HOMO->LUMO+3 (38.7%)	18Å	291	583	0.308	37.472	HOMO-1->LUMO+2 (43%)
4	16Å	288	576	0.037	67.53	HOMO-1->LUMO+2 (34.9%)	17Å	290	580	0.051	65.660	HOMO-1->LUMO+2 (35.8%)	18Å	289	579	0.127	31.797	HOMO->LUMO+3 (42.8%)
5	16Å	282	564	0.089	87.026	-	17Å	283	567	0.067	58.602	-	18Å	281	562	0.568	25.635	-
6	16Å	279	558	2.665	8.702	HOMO->LUMO+5 (21.5%)	17Å	280	561	1.762	3.2295	HOMO->LUMO+1 (21.6%)	18Å	277	555	2.688	3.661	HOMO->LUMO+5 (29.7%)
7	16Å	276	553	0.156	1.598	HOMO->LUMO+1 (42%)	17Å	279	558	1.366	3.140	HOMO->LUMO+1 (21.4%)	18Å	275	551	0.095	0.777	HOMO->LUMO (47.1%)
8	16Å	271	542	0.052	2.008	HOMO-1->LUMO+1 (42.2%)	17Å	273	547	0.089	1.281	HOMO-1->LUMO+1 (45.5%)	18Å	268	537	0.019	2.302	HOMO-1->LUMO+1 (56%)
9	16Å	267	535	0.009	31.908	HOMO->LUMO+8 (25.3%)	17Å	267	535	0.006	40.250	-	18Å	264	529	0.004	47.605	-
10	16Å	265	530	0.029	8.291	HOMO-1->LUMO+8 (22.1%)	17Å	265	531	0.034	15.814	-	18Å	263	527	0.032	16.560	-

6.4.2 Rotation angle: θ= 30°

Table S20. TD-DFT excitation summary for the H_4TCPE dimer at ϑ= 30°, comparing stacking distances of 4 and 5 Å for $d_{C\text{-}C}$= 16-18 Å. Listed are 1PA/2PA wavelengths, oscillator strengths, 2PA cross sections (σ_2), and dominant orbital transition.

θ = 30°(stacking 4 Å)

Exc		1PA [nm]	2PA [nm]	Osc. Str.	2PA [GM]	Transition		1PA [nm]	2PA [nm]	Osc. Str.	2PA [GM]	Transition		1PA [nm]	2PA [nm]	Osc. Str.	2PA [GM]	Transition
1		337	675	0.181	0.058	HOMO->LUMO (46.7%)		346	692	0.219	0.094	HOMO-1->LUMO (30.9%)		351	703	0.355	0.091	HOMO-1->LUMO (58.6%)
2		331	662	1.550	0.579	HOMO-1->LUMO (43%)		342	685	1.110	0.519	HOMO-1->LUMO+1 (32.6%)		349	699	0.662	0.429	HOMO->LUMO+1 (42.5%)
3		283	566	0.218	23.447	HOMO->LUMO+2 (29.5%)		285	571	0.032	36.514	HOMO->LUMO+2 (19.2%)		287	575	0.009	19.235	HOMO-1->LUMO+4 (19.5%)
4		281	563	0.133	20.407	HOMO->LUMO+3 (30.7%)		284	568	0.367	1.714	-		287	574	0.110	30.845	HOMO->LUMO+4 (22.7%)
5	16Å	279	559	0.032	106.50	HOMO->LUMO+5 (29.3%)	17Å	283	566	0.035	70.246	HOMO->LUMO+4 (21.9%)	18Å	285	571	0.314	7.513	-
6		277	554	2.719	1.910	HOMO->LUMO+4 (31.4%)		278	557	3.018	3.318	HOMO-1->LUMO+2 (21.4%)		280	560	2.561	5.672	HOMO-1->LUMO+2 (26.2%)
7		270	540	0.022	0.476	HOMO-1->LUMO (45.4%)		275	550	0.010	0.714	HOMO->LUMO+1 (48.1%)		278	556	0.610	0.609	HOMO->LUMO (47.8%)
8		269	539	0.003	0.079	HOMO->LUMO (41.5%)		275	550	0.065	0.111	HOMO-1->LUMO (47.5%)		276	552	0.146	1.5597	HOMO-1->LUMO+1 (56.6%)
9		265	530	0.010	17.797	HOMO->LUMO+8 (28.3%)		266	532	0.008	18.932	HOMO->LUMO+8 (24.3%)		266	532	0.008	10.295	HOMO->LUMO+8 (24.7%)
10		264	529	0.004	16.114	HOMO-1->LUMO+8 (28.3%)		265	531	0.003	13.949	HOMO-1->LUMO+8 (24.5%)		265	531	0.010	9.860	HOMO-1->LUMO+8 (28.1%)

θ = 30°(stacking 5 Å)

Exc		1PA [nm]	2PA [nm]	Osc. Str.	2PA [GM]	Transition		1PA [nm]	2PA [nm]	Osc. Str.	2PA [GM]	Transition		1PA [nm]	2PA [nm]	Osc. Str.	2PA [GM]	Transition
1		334	668	0.188	0.152	HOMO->LUMO (34.1%)		341	682	0.212	0.109	HOMO-1->LUMO (35.8%)		347	694	0.382	0.103	HOMO-1->LUMO (74.9%)
2		328	657	1.570	0.505	HOMO-1->LUMO (45.6%)		337	675	1.179	0.505	HOMO->LUMO+1 (48.5%)		344	688	0.698	0.514	HOMO->LUMO+1 (73.6%)
3		282	565	0.321	5.600	HOMO-1->LUMO+2 (20.3%)		284	569	0.054	33.479	HOMO-1->LUMO+2 (20.1%)		287	574	0.010	27.95	HOMO-3->LUMO (23.6%)
4		282	565	0.029	38.055	HOMO->LUMO+3 (17.4%)		284	568	0.289	11.188	HOMO->LUMO+3 (27.2%)		286	572	0.107	33.315	HOMO->LUMO+5 (30.2%)
5	16Å	280	561	0.065	115.77	HOMO->LUMO+5 (28.4%)	17Å	283	566	0.102	72.568	HOMO->LUMO+5 (30.5%)	18Å	285	570	0.346	6.837	HOMO-1->LUMO+2 (19.8%)
6		278	556	2.888	3.474	HOMO->LUMO+4 (25.7%)		279	558	3.189	3.801	HOMO-1->LUMO+2 (19.1%)		280	560	3.337	4.995	HOMO-1->LUMO+2 (21.6%)
7		267	534	0.001	0.273	HOMO->LUMO (52.1%)		272	544	0.002	0.046	HOMO->LUMO (79.5%)		274	549	0.044	0.321	HOMO->LUMO (85.3%)
8		265	531	0.010	0.376	HOMO-1->LUMO+1 (56.5%)		269	538	0.001	0.572	HOMO-1->LUMO+1 (80.9%)		270	541	0.006	1.683	HOMO-1->LUMO+1 (87.3%)
9		263	527	0.011	25.377	HOMO->LUMO+8 (32.4%)		264	529	0.007	22.178	HOMO-1->LUMO+8 (26%)		264	529	0.004	15.179	HOMO-1->LUMO+8 (20%)
10		263	527	0.001	10.402	HOMO-1->LUMO+8 (34.6%)		264	528	0.002	16.752	HOMO->LUMO+11 (24%)		264	528	0.006	14.178	HOMO->LUMO+11 (21.8%)

6.4.3 Rotation angle: θ= 60°

Table S21. TD-DFT excitation summary for the H_4TCPE dimer at ϑ= 60°, comparing stacking distances of 4 and 5 Å for $d_{C\text{-}C}$= 16-18 Å. Listed are 1PA/2PA wavelengths, oscillator strengths, 2PA cross sections (σ_2), and dominant orbital transition.

θ = 60°(stacking 4 Å)																			
**Exc**		**1PA [nm]**	**2PA [nm]**	**Osc. Str.**	**2PA [GM]**	**Transition**		**1PA [nm]**	**2PA [nm]**	**Osc. Str.**	**2PA [GM]**	**Transition**		**1PA [nm]**	**2PA [nm]**	**Osc. Str.**	**2PA [GM]**	**Transition**
1	**16Å**	338	677	0.961	0.386	HOMO->LUMO (38.9%)	**17Å**	342	684	0.747	0.671	HOMO-1->LUMO (37.4%)	**18Å**	359	718	0.497	0.290	HOMO->LUMO (54%)
2		336	673	0.509	0.573	HOMO-1->LUMO (45.6%)		338	677	0.417	0.443	HOMO->LUMO+1 (56.5%)		352	704	0.381	0.256	HOMO->LUMO+1 (42.8%)
3		286	573	0.030	25.639	HOMO->LUMO+3 (24.4%)		286	572	0.572	18.088	HOMO->LUMO+2 (22.7%)		299	599	0.292	6.026	HOMO->LUMO+2 (35.7%)
4		285	571	1.048	2.840	HOMO->LUMO+2 (20.9%)		282	564	0.154	79.180	HOMO->LUMO+3 (30.9%)		294	588	0.616	4.651	HOMO->LUMO+3 (31.1%)
5		283	566	0.061	126.30	HOMO->LUMO+4 (22.1%)		279	559	0.969	18.200	-		288	577	0.282	57.845	HOMO-1->LUMO+2 (26%)
6		281	563	2.171	11.030	HOMO->LUMO+5 (22.6%)		276	553	1.121	32.780	-		286	573	0.811	13.374	HOMO-1->LUMO (28.7%)
7		271	543	0.154	0.655	HOMO->LUMO (41.7%)		272	545	0.601	2.933	HOMO->LUMO (40.6%)		278	557	1.625	1.169	HOMO->LUMO+6 (24.3%)
8		269	538	0.101	0.274	HOMO-1->LUMO+1 (45.5%)		269	538	0.057	0.734	HOMO-1->LUMO+1 (55.3%)		278	557	0.150	0.080	HOMO-1->LUMO+1 (35.8%)
9		267	534	0.008	3.225	HOMO->LUMO+8 (35.3%)		266	532	0.074	4.965	HOMO->LUMO+8 (25.6%)		274	549	0.016	7.108	-
10		266	532	0.000	28.160	HOMO-1->LUMO+8 (34.4%)		264	529	0.046	40.545	HOMO-1->LUMO+8 (24.4%)		267	534	0.009	22.131	HOMO-1->LUMO+9 (30.1%)
**θ = 60°(stacking 5 Å)**																			
1	**16Å**	338	676	0.881	0.572	HOMO->LUMO (64.9%)	**17Å**	350	701	0.642	4.058	HOMO->LUMO+1 (66.6%)	**18Å**	348	697	0.567	0.306	HOMO->LUMO (64.4%)
2		333	666	0.691	0.148	HOMO-1->LUMO+1 (48.4%)		342	685	0.556	2.973	HOMO-1->LUMO (70.1%)		341	682	0.398	0.509	HOMO-1->LUMO+1 (45.5%)
3		288	577	0.164	40.031	HOMO->LUMO+3 (27%)		291	583	0.621	35.599	HOMO->LUMO+3 (54.9%)		289	578	0.455	14.465	HOMO->LUMO+2 (35.8%)
4		286	573	1.124	10.197	HOMO->LUMO+2 (33%)		289	578	0.596	44.595	HOMO-1->LUMO+2 (42.5%)		283	567	0.439	37.020	-
5		284	568	1.142	65.600	HOMO-1->LUMO+3 (28.3%)		284	569	0.233	27.580	HOMO->LUMO (52.6%)		283	566	0.843	14.578	HOMO->LUMO+3 (19.5%)
6		280	560	1.031	39.212	HOMO-1->LUMO+5 (38.2%)		280	560	0.587	42.736	HOMO-1->LUMO+4 (27.8%)		278	557	1.617	25.350	-
7		269	538	0.091	0.232	HOMO-1->LUMO (61.1%)		278	556	1.385	12.665	HOMO->LUMO+5 (28.1%)		273	546	0.403	2.713	HOMO-1->LUMO (52.4%)
8		267	535	0.055	0.817	HOMO->LUMO+1 (61.9%)		270	541	0.075	2.244	HOMO-1->LUMO+1 (79.5%)		271	543	0.052	1.480	HOMO->LUMO+1 (53.6%)
9		267	534	0.009	8.586	HOMO->LUMO+8 (32.5%)		268	536	0.080	4.098	HOMO->LUMO+8 (22.8%)		267	534	0.031	3.668	HOMO->LUMO+8 (28%)
10		265	530	0.014	20.803	HOMO-1->LUMO+8 (31.7%)		266	533	0.056	28.273	HOMO-1->LUMO+8 (24%)		264	528	0.014	25.791	HOMO-1->LUMO+8 (23.2%)

6.4.4 Rotation angle: θ= 90°

Table S22. TD-DFT excitation summary for the H_4TCPE dimer at ϑ= 90°, comparing stacking distances of 4 and 5 Å for $d_{C\text{-}C}$= 16-18 Å. Listed are 1PA/2PA wavelengths, oscillator strengths, 2PA cross sections (σ_2), and dominant orbital transition.

θ = 90°(stacking 4 Å)																			
**Exc**		**1PA [nm]**	**2PA [nm]**	**Osc. Str.**	**2PA [GM]**	**Transition**		**1PA [nm]**	**2PA [nm]**	**Osc. Str.**	**2PA [GM]**	**Transition**		**1PA [nm]**	**2PA [nm]**	**Osc. Str.**	**2PA [GM]**	**Transition**
1		341	682	0.729	1.709	HOMO->LUMO+1 (55.8%)		351	703	0.600	0.663	HOMO->LUMO+1 (69.8%)		365	731	0.421	0.074	HOMO->LUMO+1 (57.9%)
2		333	667	0.783	5.503	HOMO->LUMO (31.5%)		341	682	0.651	1.788	HOMO-1->LUMO (57.8%)		359	719	0.540	0.635	HOMO->LUMO (47.5%)
3		298	596	0.511	1.901	HOMO->LUMO+2 (34.9%)		295	590	0.249	4.473	-		298	597	0.243	0.459	HOMO->LUMO+2 (26.6%)
4		290	581	0.863	48.149	-		291	583	1.189	2.263	-		295	591	0.549	0.188	HOMO-1->LUMO (30.4%)
5	**16Å**	289	578	1.485	20.458	-	**17Å**	289	579	1.150	50.416	HOMO-1->LUMO+2 (27.6%)	**18Å**	294	588	0.505	7.804	HOMO-1->LUMO+1 (47.9%)
6		284	568	0.435	85.368	HOMO->LUMO+5 (23.9%)		287	574	0.391	72.713	HOMO->LUMO+5 (24.8%)		292	585	0.002	74.167	-
7		277	554	0.072	16.143	HOMO->LUMO (33.3%)		283	567	0.717	4.693	HOMO->LUMO (33.5%)		287	574	1.601	1.607	HOMO->LUMO+4 (23.9%)
8		273	546	0.183	2.386	HOMO-1->LUMO+1 (34.3%)		275	551	0.128	0.881	HOMO-1->LUMO+1 (62.7%)		286	573	1.044	2.079	-
9		271	542	0.027	1.831	-		270	541	0.009	4.518	HOMO->LUMO+8 (38.7%)		271	542	0.009	0.147	HOMO->LUMO+8 (40.4%)
10		268	536	0.046	2.436	HOMO->LUMO+8 (24.2%)		266	533	0.003	30.998	HOMO-1->LUMO+8 (30%)		268	536	0.001	25.386	HOMO-1->LUMO+8 (32.4%)
**θ = 90°(stacking 5 Å)**																			
1		335	670	0.678	1.957	HOMO-1->LUMO (39.7%)		345	691	0.585	0.266	HOMO->LUMO+1 (63.8%)		361	722	0.397	0.087	HOMO->LUMO (34.1%)
2		331	663	0.833	0.606	HOMO->LUMO+1 (38.7%)		339	678	0.698	0.486	HOMO-1->LUMO (44.2%)		355	711	0.590	0.443	HOMO->LUMO+1 (29.6%)
3		293	586	0.494	17.090	HOMO-1->LUMO+2 (29.9%)		292	584	0.395	20.046	HOMO-1->LUMO+2 (27.9%)		294	589	0.175	1.963	HOMO->LUMO+2 (30.5%)
4		289	578	1.249	20.105	HOMO->LUMO+4 (33.8%)		288	577	1.331	3.163	HOMO->LUMO+3 (23.8%)		292	584	0.859	0.971	HOMO-1->LUMO+1 (21.4%)
5	**16Å**	285	570	1.293	2.054	-	**17Å**	285	571	0.864	60.597	HOMO->LUMO+4 (26.9%)	**18Å**	290	581	0.428	4.752	HOMO-1->LUMO (32.7%)
6		283	567	0.217	137.64	HOMO-1->LUMO+3 (22.4%)		284	569	0.730	51.081	HOMO->LUMO+5 (28.1%)		289	579	0.081	75.022	HOMO-1->LUMO+2 (22.2%)
7		278	556	0.443	12.572	HOMO->LUMO (48.3%)		278	557	0.434	1.487	HOMO->LUMO (40.6%)		284	569	1.322	3.131	HOMO->LUMO+4 (25%)
8		267	534	0.018	0.704	HOMO-1->LUMO+8 (26.8%)		274	548	0.130	0.555	HOMO-1->LUMO+1 (54.2%)		283	566	1.108	2.892	HOMO-1->LUMO+4 (20.5%)
9		265	531	0.057	21.183	-		268	536	0.015	2.643	HOMO->LUMO+8 (39.7%)		269	538	0.015	0.414	HOMO->LUMO+8 (41.1%)
10		265	530	0.024	32.502	-		265	531	0.004	37.740	HOMO-1->LUMO+8 (32.8%)		266	533	0.000	33.000	HOMO-1->LUMO+8 (32.5%)

6.4.5 EDD and main orbital transitions: comparison of 4 Å vs 5 Å at θ= 0°.

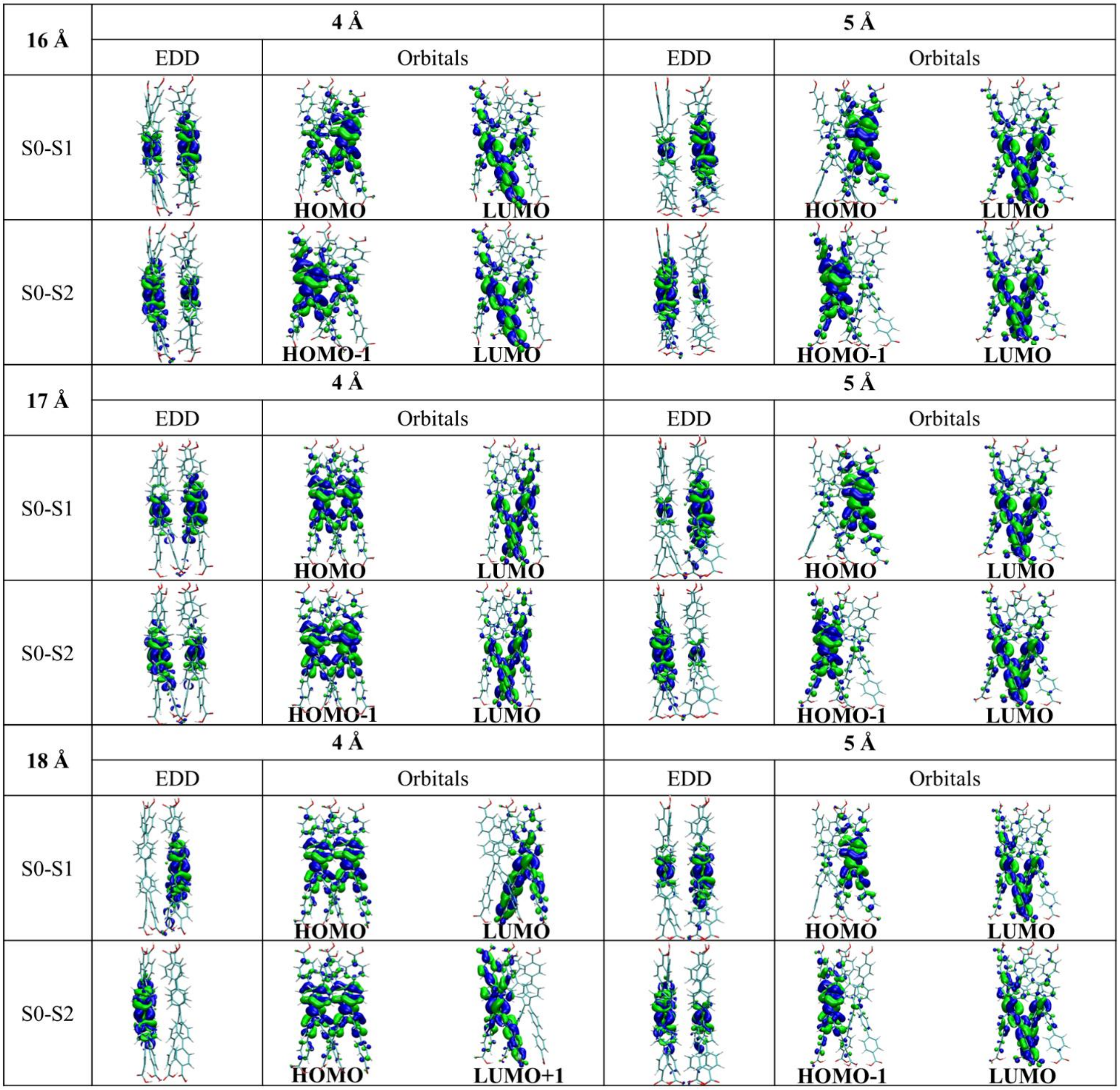

Figure S20. Comparison of stacking distances (4 Å and 5 Å) for d_{C-C}= 16-18 Å at ϑ= 0° rotation displacement. EDD plots and dominant orbital transitions for the first and second excitations.

6.4.6 Polarizability results: θ= 0° (or named Δy = 0)

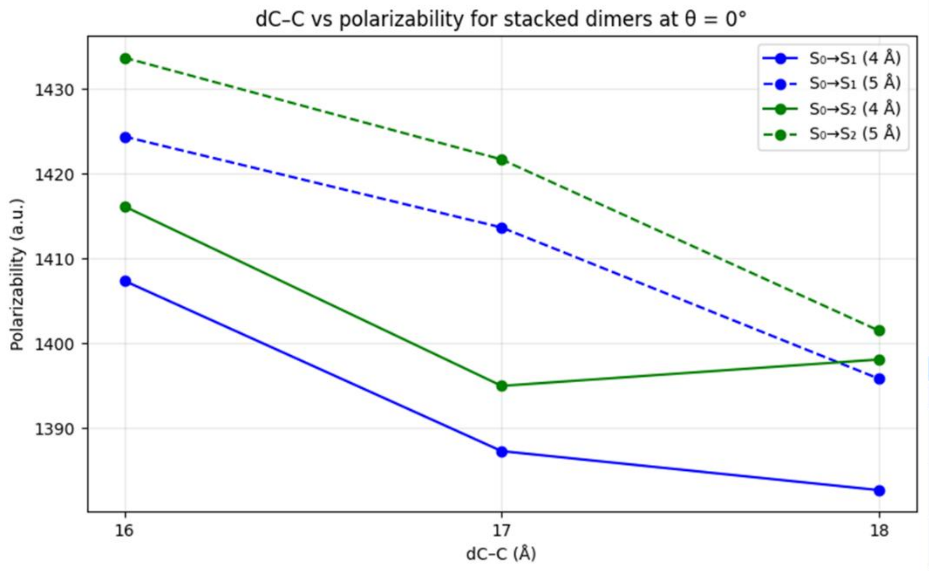

Polarizabilities for θ=0°

dC-C	excitation	4 A	5 A
16	S0-S1	1407.35	1424.36
	S0-S2	1416.13	1433.661
17	S0-S1	1387.27	1413.64
	S0-S2	1394.96	1421.66
18	S0-S1	1382.67	1395.81
	S0-S2	1398.07	1401.476

Figure S21. Isotropic polarizability values across the sampled distances for the first and second excitations of parallel stacking systems at two different initial stacking distances Δz= 4.

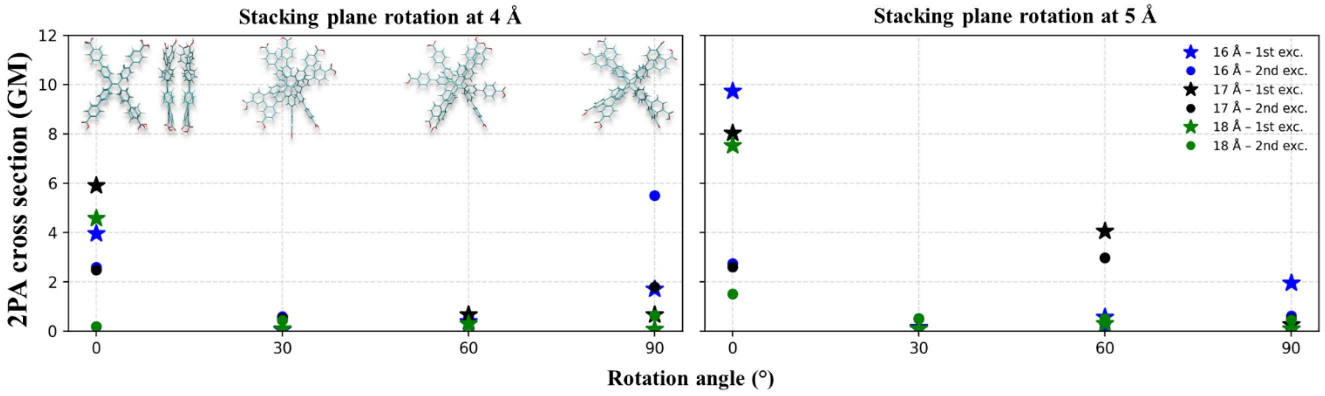

Figure S22. Effect of stacking-plane rotation on the 2PA of H_4TCPE dimers. σ_2 as a function of stacking-plane rotation angle at an initial separation of 4 Å (left) and 5 Å (right). Stars and circles denote the first and second excited-state transitions, respectively. Colors indicate the intramolecular $d_{C\text{-}C}$: 16 Å (blue), 17 Å (black), and 18 Å (green). Representative geometries for selected configurations are shown as insets.

7 References

[1] S. G. Balasubramani, G. P. Chen, S. Coriani, M. Diedenhofen, M. S. Frank, Y. J. Franzke, F. Furche, R. Grotjahn, M. E. Harding, C. Hättig, A. Hellweg, B. Helmich-Paris, C. Holzer, U. Huniar, M. Kaupp, A. Marefat Khah, S. Karbalaei Khani, T. Müller, F. Mack, B. D. Nguyen, S. M. Parker, E. Perlt, D. Rappoport, K. Reiter, S. Roy, M. Rückert, G. Schmitz, M. Sierka, E. Tapavicza, D. P. Tew, C. van Wüllen, V. K. Voora, F. Weigend, A. Wodyński, J. M. Yu. "TURBOMOLE: Modular program suite for *ab initio* quantum-chemical and condensed-matter simulations." *The Journal of Chemical Physics* **2020**, *152*, 184107. DOI: 10.1063/5.0004635

[2] K. Momma, F. Izumi. "VESTA 3 for three-dimensional visualization of crystal, volumetric and morphology data." *Journal of Applied Crystallography* **2011**, *44*, 1272. DOI: 10.1107/S0021889811038970
